# Clerodane diterpenoid glycosides from the tuberous roots of *Paratinospora sagittata*: targeted isolation, structure characterization and immunomodulatory properties

**DOI:** 10.1007/s13659-025-00555-2

**Published:** 2026-02-02

**Authors:** Jun-Sheng Zhang, Rui Ao, Yin-Bo Pan, Xin-Cheng Zhuang, Yi-Ke Yin, Jie Bao, Hua Zhang

**Affiliations:** School of Biological Science and Technology, University of Jinan, Jinan, 250022 China

**Keywords:** *Paratinospora sagittata*, Clerodane, Diterpenoid glycosides, Absolute configuration, Immunomodulatory

## Abstract

Guided by the MS/MS-based molecular networking, eight previously undescribed clerodane diterpenoid glycosides, designated tinospinosides F–M (**1**−**8**), along with 12 known analogues (**9**−**20**), were isolated from the tuberous roots of *Paratinospora sagittata*. Structural elucidation of the undescribed compounds was achieved through comprehensive spectroscopic analyses (NMR, HRESIMS), with their absolute configurations confirmed via single-crystal X-ray diffraction, TD-DFT/ECD computational analyses, and chemical degradation. Immunomodulation evaluation on all the isolates revealed that compounds **6** and **7** exerted significant promoting effect toward NO production in RAW264.7 macrophages. Further study demonstrated that **6** could enhance the release of immune cytokines (e.g., TNF-*α*) and upregulate the protein expression of iNOS and COX-2, which was potentially mediated through the activation of NF-κB signaling pathway.

**Graphical Abstract:**

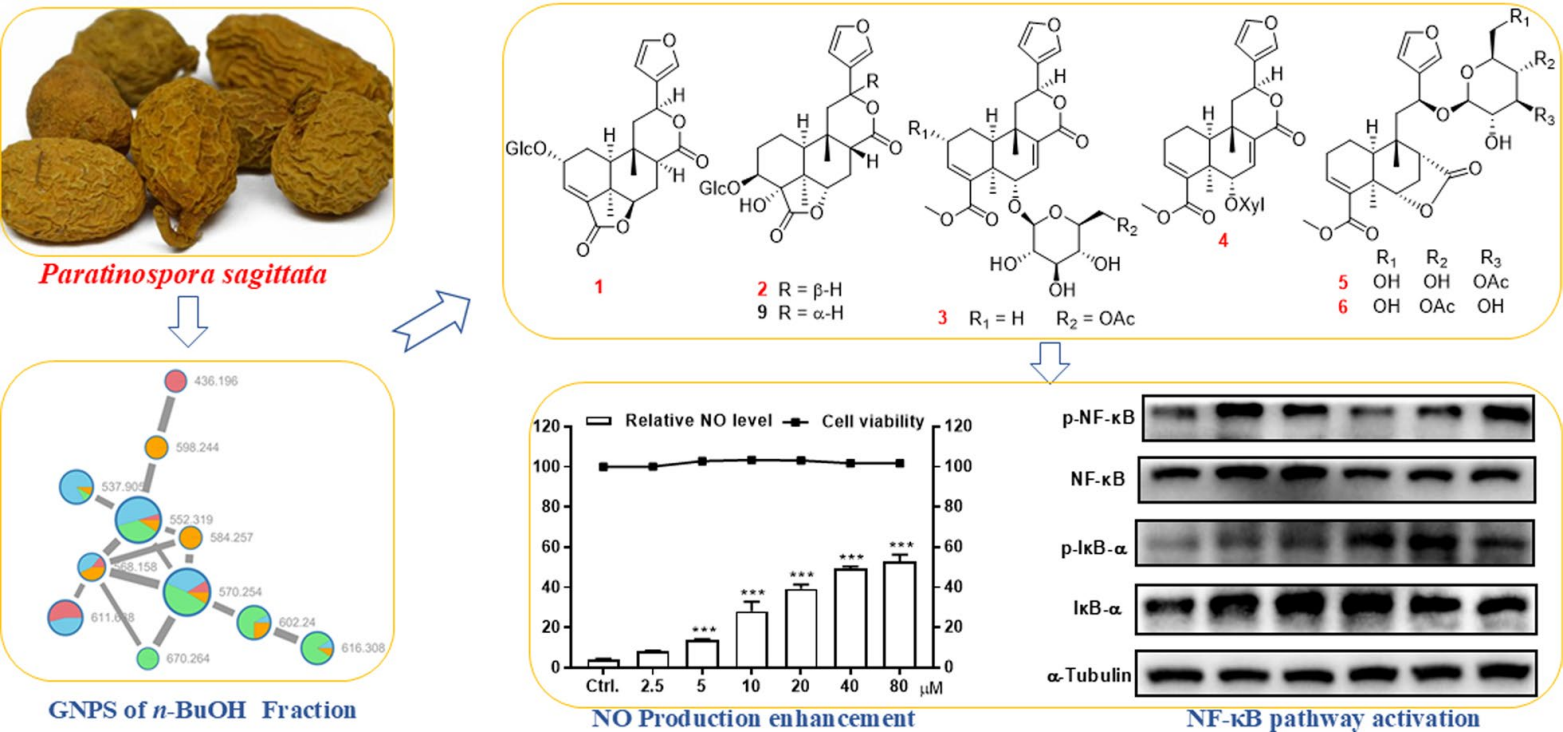

**Supplementary Information:**

The online version contains supplementary material available at 10.1007/s13659-025-00555-2.

## Introduction

The genera *Paratinospora* and *Tinospora*, two closely related genera in the family Menispermaceae, are predominantly found in the tropical and subtropical regions of Asia, Australia, and Africa [1–3]. Before 2025, plants of genus *Paratinospora* (*P. sagittata* and *P. dentata*) belong to the genus *Tinospora*, while Wei Wang et al. formally established the new genus *Paratinospora* based on a combined analysis of plastid and nuclear DNA sequence data [4, 5]. Medicinal plants in the *Paratinospora*/*Tinospora* genera are extensively employed as folk medicine in countries of Southeast Asia as immunomodulatory agents [6–8]. Bioactive constituents in genera *Paratinospora*/*Tinospora* such as alkaloids and diterpenoids also demonstrate significant activities including anti-inflammatory, antimicrobial, antiviral and antioxidant effects [1–3]. *Paratinospora sagittata* (Oliv.) Wei Wang, commonly known as “Jin Guo Lan” in traditional Chinese medicine, holds a prominent position in the Chinese Pharmacopeia [9]. Its rhizomes are extensively utilized for their therapeutic potential in fever reduction, detoxification, alleviation of swelling and pain management [10]. Previous studies on the chemical constituents of this species have primarily focused on the EtOAc soluble part of its EtOH extract, with the main components identified as diterpenoids [11–15]. To the best of our knowledge, the *n*-BuOH soluble part has not been investigated before.

As a continuation of our research on diterpenoids from family Menispermaceae [16–18], the current study employed a targeted isolation using MS/MS-based molecular networking analysis, which revealed characteristic signatures of clerodane diterpenoid glycosides within the *n*-BuOH soluble part of *P. sagittata*. This approach led to the isolation and identification of 20 clerodane diterpenoid glycosides, including eight previously undescribed compounds. The structures of the undescribed compounds were determined through extensive spectroscopic analyses, with their absolute configurations further confirmed by single-crystal X-ray diffraction, TD-DFT/ECD computational method, and chemical methods. The immunomodulatory effects of all isolated compounds were assessed in RAW264.7 cells. This study details the isolation, structure elucidation, and the observed immunomodulatory activities of these compounds.

## Results and discussion

### Molecular networking analysis

**Fig. 1:**
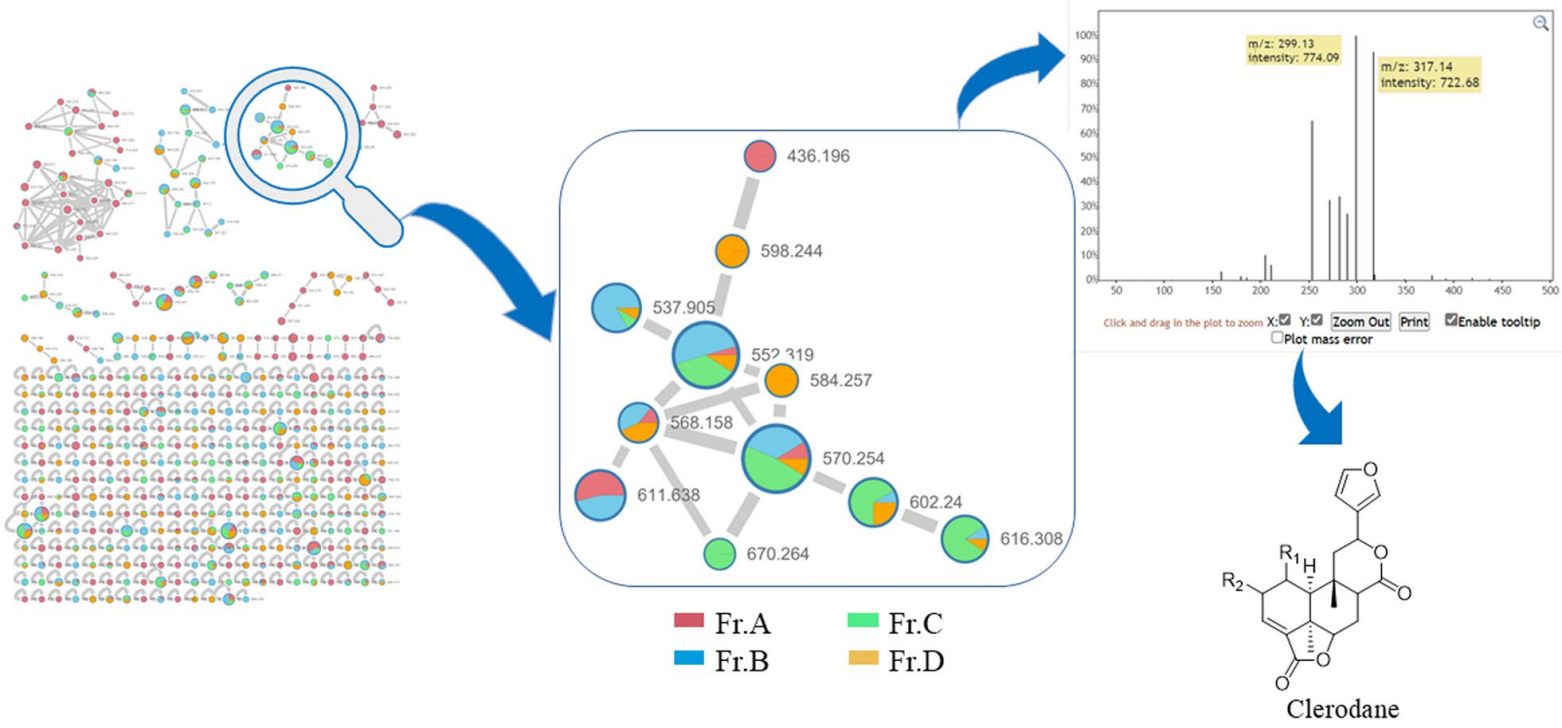
MS/MS-based molecular networking of the *n*-BuOH fraction of *P. sagittate*

**Fig. 2:**
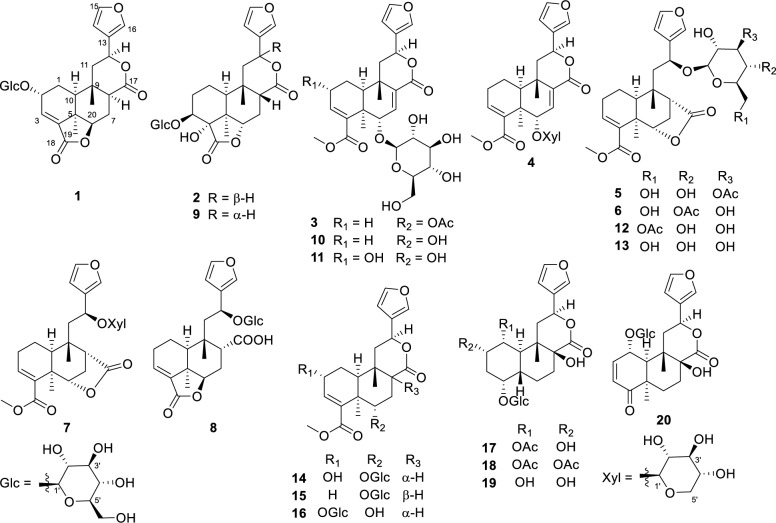
Chemical structures of compounds **1**–**20**

Tuberous roots of *P. sagittata* (30 kg) were powdered and then extracted with 95% EtOH at room temperature to give a residue, which was suspended in H_2_O and then partitioned successively with EtOAc and *n*-BuOH. The *n*-BuOH partition was further separated into four fractions (A−D) by D101 macroporous resin and silica gel column chromatographies. Subsequently, HPLC-HRESIMS/MS data of the four fractions were uploaded to Global Natural Products Social Molecular Networking database to construct a molecular network, which revealed three major clusters and several minor clusters. According to the previous recognition of the MS/MS data of clerodane diterpenoids, the diagnostic ions are the fragment ions at *m*/*z* 377 → *m*/*z* 317 → *m*/*z* 299 [18]. Then, the fragment ions at *m*/*z* 317 → 299 in the MS–MS data of one cluster in the *n*-BuOH fraction suggested the presence of clerodane diterpenoids, which primarily presented in fractions B and C (see Fig. 1). Subsequently, further in-depth investigation led to the isolation of eight undescribed clerodane diterpenoids and 12 known analogues (**1**−**20**, Fig. 2).

### Structure elucidation

**Fig. 3:**
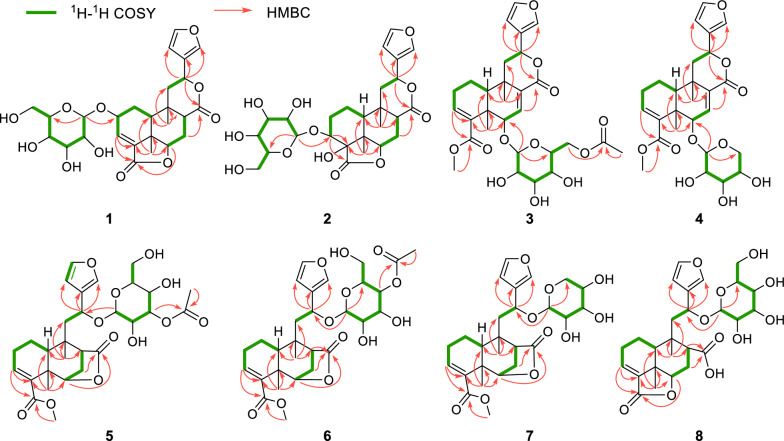
Selected ^1^H−^1^H COSY and HMBC cross-peaks for compounds **1**−**8**

Compound **1**, a colorless gum, had the molecular formula of C_26_H_32_O_11_ established by HRESIMS (11 degrees of unsaturation). 1D NMR and HSQC data revealed signals for two ester carbonyl groups (*δ*_C_ 175.8 and 171.0), a *β*-substituted furan unit [*δ*_H_ 6.53 (1H, dd,* J* = 1.8, 0.7 Hz), 7.52 (1H, t,* J* = 1.7 Hz) and 7.61 (1H, m); *δ*_C_ 109.7, 125.4, 141.5 and 145.2], a trisubstituted double bond [*δ*_H_ 6.84 (1H, d,* J* = 3.6 Hz); *δ*_C_ 136.7 and 137.5], a *β*-glucose moiety [*δ*_H_ 3.18, 3.28, 3.33, 3.35, 3.67, 3.90 and 4.50 (1H, d,* J* = 7.8 Hz); *δ*_C_ 62.8, 71.8, 75.1, 78.0, 78.1 and 104.1] and two methyl groups [*δ*_H_ 0.94 and 1.39 (each 3H, s); *δ*_C_ 22.7 and 30.2]. The gross structure for **1** was accomplished by interpretation of 2D NMR data, especially HMBC and ^1^H−^1^H COSY data (Fig. 3). In the ^1^H−^1^H COSY spectrum, the cross peaks of H-10/H_2_-1/H-2/H-3 suggested the presence of the key fragment (C-10/C-1/C-2/C-3). Then, the HMBC correlations from protons of a tertiary methyl (H_3_-19) to C-4, C-5 and C-10 established the formation of ring A. Moreover, the ^1^H−^1^H COSY correlations of H-6/H_2_-7/H-8 and the HMBC correlations from H_2_-7 and H-10 to C-8 and C-9, together with the HMBC correlations from H_3_-20 to C-8, C-9 and C-10 constructed the six-membered ring B. The ^1^H − ^1^H COSY correlations from H_2_-11 to H-12 together with the HMBC correlations from H-14 and H-16 to C-12 attached the furan ring to C-12. Key HMBC correlation from H-1′ (*δ*_H_ 4.50) to C-2 suggested that the sugar moiety was attached to C-2. As nine of 11 degrees of unsaturation were accounted by the aforementioned furan ring, double bond, carbonyl groups, sugar moiety and the basic 6/6 bicyclic framework, the remaining two degrees of unsaturation required two additional rings in the structure of **1**. Thus, the key HMBC correlation from H-12 to the ester carbonyl carbon at *δ*_C_ 174.6 (C-17) and H-6 to another ester carbonyl carbon at *δ*_C_ 171.0 (C-18) indicated the formation of 12,17-lactone bridge and 6,18-lactone bridge, respectively.

**Fig. 4:**
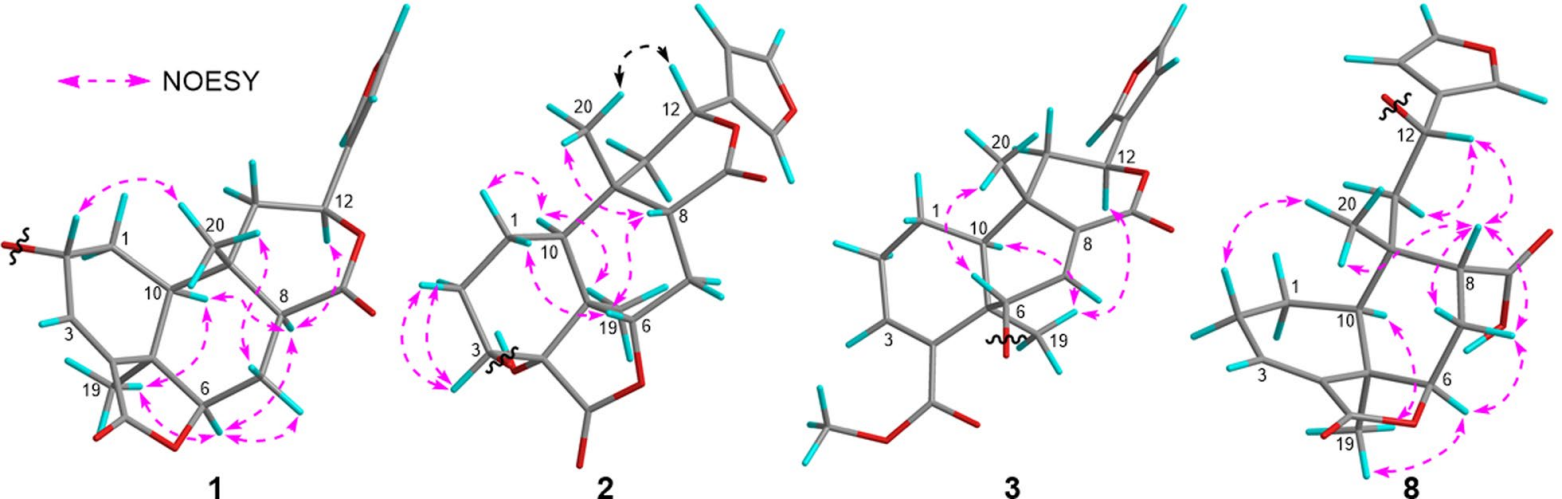
Key NOESY correlations for compounds **1**−**3** and **8**

**Fig. 5:**
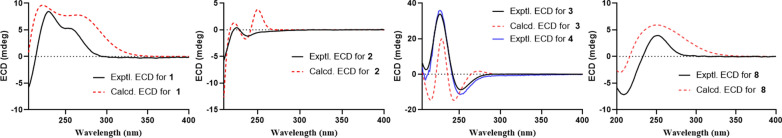
Experimental ECD spectra of **1**−**4** and **8** and calculated ECD spectra of **1**−**3** and **8**

The relative configuration of **1** was mainly based on NOESY spectrum (Fig. 4). NOESY correlations of H_3_-19/H-6 and H-10, H-6/H-8 and H-7 at *δ*_H_ 2.47, H-8/H-10 and H-12, indicated their spatial proximity and a random determination of the *α*-orientation of these protons. Conversely, NOESY cross peaks between the H_3_-20 with H-2 and H-7 at *δ*_H_ 1.43, revealed the *β*-orientation for CH_3_-20 and H-2. The *β*-configuration of the anomeric proton was established by analyzing the coupling constant of the anomeric proton (*J* = 7.8 Hz). Subsequent acid hydrolysis confirmed that the retention time of the obtained product matched exactly with that of the standard d-glucose. Finally, the experimental ECD spectrum of **1** matched the calculated ECD spectrum of the (2*R*,5*R*,6*R*,8*S*,9*S*,10*S*,12*S*)-isomer (Fig. 5). Thus, the structure of **1** was fully characterized and was given the trivial name tinospinoside F, following tinospinosides A−E reported from the same species [19, 20].

HRESIMS analysis of compound **2** revealed a quasi-molecular ion at *m*/*z* 556.2393 [M + NH_4_]^+^ (calcd for C_26_H_38_O_12_N^+^, 556.2389), indicative of a molecular formula of C_26_H_34_O_12_ and isomeric with borapetoside A (**9**) [21]. The 1D NMR data of **2** showed high similarity to those of **9** with the major differences being attributed to the chemical shift of H-12 (*δ*_H_ 5.63 in **2**; *δ*_H_ 5.90 in **9**), indicating that **2** was the 12-epimer of **9**. Detailed 2D NMR data analyses further secured the planar structure of **2**, which was the same as that of **9**. The relative configuration of **2** was established mainly by NOESY experiments (Fig. 4) and 1D NMR data. The chiral centers in rings A and B were assigned to be the same as those in **9** based on the highly matched 1D NMR data. Key NOESY correlations of H_3_-20/H-12 and H-8 supported the orientation of H-12 as *β*. The absolute configuration of **2** was determined by comparison its experimental ECD curve with its theoretical one. Thus, the structure of compound **2** (tinospinoside G) was assigned.

Compound **3**, a colorless gum, possessed a molecular formula of C_29_H_32_O_12_ by analysis of its HRESIMS data (*m*/*z* 577.2279 [M + H]^+^, calcd for C_29_H_33_O_12_^+^, 577.2280). The 1D NMR data and HSQC spectrum exhibited signals for an acetyl group [*δ*_H_ 2.17 (3H, s); *δ*_C_ 172.0 and 20.8], a *β*-substituted furan unit [*δ*_H_ 7.46 (1H, brs), 7.42 (1H, brs) and 6.42 (1H, brs); *δ*_C_ 143.7, 139.8, 123.7 and 108.6], two ester carbonyl groups (*δ*_C_ 171.6 and 168.7), two trisubstituted double bonds [*δ*_H_ 6.99 (1H, s), 6.49 (1H, t, *J* = 3.4 Hz); *δ*_C_ 138.4, 136.7, 136.7 and 136.2] and two methyl groups [*δ*_H_ 1.37 (3H, s) and 1.17 (3H, s); *δ*_C_ 27.9 and 22.3]. The aforementioned NMR data of **3** exhibited high resemblance with those of **10** (borapetoside F [22]), except for the additional signals of an acetyl group. In the HMBC spectrum, the cross-peak from H_2_-6′ (*δ*_H_ 4.39 and 4.31) to the carbonyl of acetyl group (*δ*_C_ 172.0) suggested that the 6′-OH in **10** was acetylated in **3**. Detailed 2D NMR correlations including ^1^H-^1^H COSY and HMBC analysis supported that planar structure of **3** as depicted. The relative configuration of **3** was assigned the same as that of **10** by their highly-matched 1D NMR data. As shown in Fig. 5, the experimental ECD curve of **3** matched the calculated ECD spectrum of the (5*R*,6*S*,9*S*,10*S*,12*S*)-isomer. Thus, the structure of **3** was fully characterized and given the trivial name tinospinoside H.

Similar with compound **3**, the NMR data of compound **4** (tinospinoside I) also showed high resemblance with those of **10**. Extensive comparison of the 1D NMR data of **4** with those of **10** revealed that the glucopyranose unit in **10** was replaced by a xylopyranose unit in **4**. Further 2D NMR analysis supported the planar structure of **4** as shown in Fig. 3. The xylopyranose unit was determined as d-configuration by HPLC comparison with those of the standard ones. While the absolute configuration of the aglycone of **4** was the same as that of **3** by their highly-matched ECD curves.

**Fig. 6:**
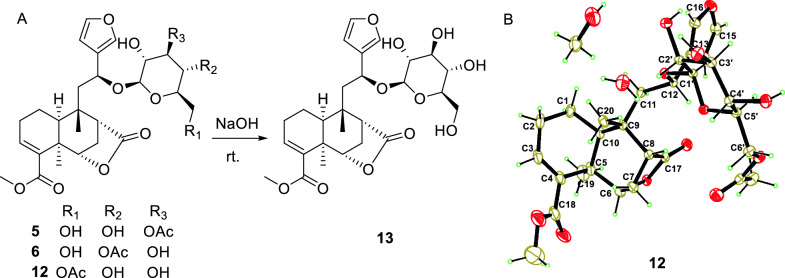
**A** Chemical transformation from **5**, **6** and **12** to **13**;** B** Ortep drawing of **12**

Compounds **5** and **6** were assigned the same molecular formula C_29_H_38_O_12_ by HRESIMS analyses and their 1D NMR data showed high resemblance. With the aid of full 2D NMR experiments, the 1D NMR data of **5** and **6** were completely assigned and exhibited high similarity to those of tinopanoid T (**12**) [23], the co-isolated analogue in current research. A detailed comparison revealed that the key structural difference among these analogues was the site of acetylation. Whereas compound **12** is acetylated at the 6′-*O* position, compounds **5** and **6** are acetylated at the3′-*O* and 4′-*O* positions, respectively. These structural assignments were unambiguously supported by the chemical shift comparisons of H-3′, H-4′ and H-6′ in the three structures, as well as their HMBC correlations to the corresponding acetyl carbonyl carbons. The configurations of **5** and **6** were determined to be identical with those of **12** based on their consistent NMR coupling patterns, NOESY data and ECD curves. Their structures were further secured by chemical correlation with **12**. The alkaline hydrolysis of **5**, **6** and **12** generated the same product **13** (Fig. 6), which was verified by comparison of their NMR data. The absolute configuration of **12** was determined by single crystal X-ray diffraction [CCDC number: 2376200; Frack parameter =  − 0.01 (10)]. Hence, the structures of **5** and **6** were established as depicted and given the trivial names tinospinosides J and K, respectively.

Compound **7** was isolated as a colorless gum and had the molecular formula of C_26_H_34_O_10_ as determined by HRESIMS. The 1D NMR data of **7** bore a resemblance to those of **13** [24], except for the replacement of the glucopyranose unit in **13** by a xylopyranose unit in **7**. This assignment was further confirmed by the ^1^H − ^1^H COSY correlations of H-1′/H-2′/H-3′/H-4′/H_2_-5′, together with the HMBC correlations of H-1′/C-12. The relative configuration of the aglycone of **7** was assigned to be the same as that of **13** by comparing their highly matched 1D NMR data. Especially, the configuration of H-12 was determined by the empirical rule established by Gao et al. [25], the coupling constants between H-12 and H-11 (*J* = 9.9, 3.3 Hz) suggested the relative configuration of H-12 was S*. The absolute configuration of **7** was determined by ECD comparison with those of **5** and **6**, nearly identical cotton effects revealed that they have the same absolute configuration in the aglycone part. Thus, compound **7** was assigned as depicted and named tinospinoside L.

Compound **8**, a colorless gum, had the molecular formula C_26_H_34_O_11_ as determined by the HRESIMS ion at *m*/*z* 545.1993 [M + Na]^+^ (calcd for C_26_H_34_O_11_Na^+^, 545.1993). The 1D NMR data together with HSQC spectrum showed signals for a *β*-substituted furan ring [*δ*_H_ 7.48 (1H, d, *J* = 1.5 Hz), 7.42 (1H, dd, *J* = 1.5, 0.9 Hz) and 6.44 (1H, d, *J* = 0.9 Hz); *δ*_C_ 144.5, 141.5, 127.9 and 110.0], a *β*-glucopyranosyl moiety [*δ*_H_ 4.15 (1H, d, *J* = 7.7 Hz), 3.88 (1H, dd, *J* = 11.8, 2.3 Hz), 3.64 (1H, dd, *J* = 11.8, 6.3 Hz), 3.23 (1H, m), 3.20 (1H, m), 3.16 (1H, m) and 3.13 (1H, m); *δ*_C_ 100.6, 78.0, 77.8, 75.3, 72.0 and 63.2], a trisubstituted double bond [*δ*_H_ 6.87 (1H, t, *J* = 3.6 Hz); *δ*_C_ 138.6 and 135.1] and two methyl groups [*δ*_H_ 1.24 (3H, s) and 1.19 (3H, s); *δ*_C_ 30.0 and 22.2]. The planar structure of **8** was mainly achieved by ^1^H-^1^H COSY and HMBC correlations. Analysis of the ^1^H-^1^H COSY spectrum revealed key correlations between H-10/H_2_-1/H_2_-2/H-3 and H-6/H_2_-7/H-8, suggesting the presence of two crucial fragments (C-10/C-1/C-2/C-3 and C-6/C-7/C-8). HMBC correlations from H_3_-19 to C-4, C-5, C-6 and C-10, and from H_3_-20 to C-8, C-9, C-10 and C-11 aided to establish the 6/6 fused rings of rings A and B. The ^1^H-^1^H COSY correlation between H_2_-11 and H-12, along with HMBC correlations from H-12 to C-13, C-14 and C-16, revealed that the furan ring was attached to C-12. A key HMBC correlation between H-6 and the ester carbonyl at *δ*_C_ 172.3 (C-18) indicated the formation of an intramolecular lactone bridge between C-18 and C-6. Furthermore, a crucial HMBC signal between H-8 and the carboxyl group at *δ*_C_ 177.9 (C-17) defined the attachment position of the carboxyl group. Finally, the HMBC correlation between H-12 (*δ*_H_ 5.25) and the anomeric carbon (*δ*_C_ 100.6) confirmed the linkage of the glucose unit to C-12. The relative configuration of **8** was mainly determined by NOESY correlations and ^1^H-^1^H coupling constants. Key NOESY correlations of H_3_-19/H-6, H-10 and H-6/H-7 at *δ*_H_ 2.27 suggested that these protons were cofacial, leading to a tentative assignment of an *α*-orientation for these protons. While the NOESY correlations from H_3_-20 to H-8 and H-7 at *δ*_H_ 1.59, together with the small coupling constants between H-8 and H_2_-7 (*J* = 4.4, 3.4 Hz) suggested that these protons were in *β*-orientation. The coupling constant between H_2_-11 and H-12, interpreted similarly to the case of **7**, suggested an S* configuration at H-12. The absolute configuration of **8** was established as shown by comparing the ECD curve with the calculated one. Thus, compound** 8** was assigned as depicted and named tinospinoside M.

The known compounds borapetoside A (**9**) [21], borapetoside F (**10**) [22], (2*R*,5*R*,6*S*,9*S*,10*S*,12*S*)-15,16-epoxy-2-hydroxy-6-*O*-(*β*-D-glucopyranosyl)-cleroda-3,7,13(16),14-tetraen-17,12-olid-18-oic acid methyl ester (**11**) [21], tinopanoid T (**12**) [23], rumphioside I (**13**) [24], borapetoside B (**14**) [21], borapetoside C (**15**) [21], tinospinoside C (**16**) [26], tinosineside A (**17**) [27], tinosineside B (**18**) [16], 1-deacetyltinosposide A (**19**) [27], tinosinenoside B (**20**) [28] were determined by comparing their NMR data with those in the literature.

### Immunomodulatory activity

**Fig. 7:**
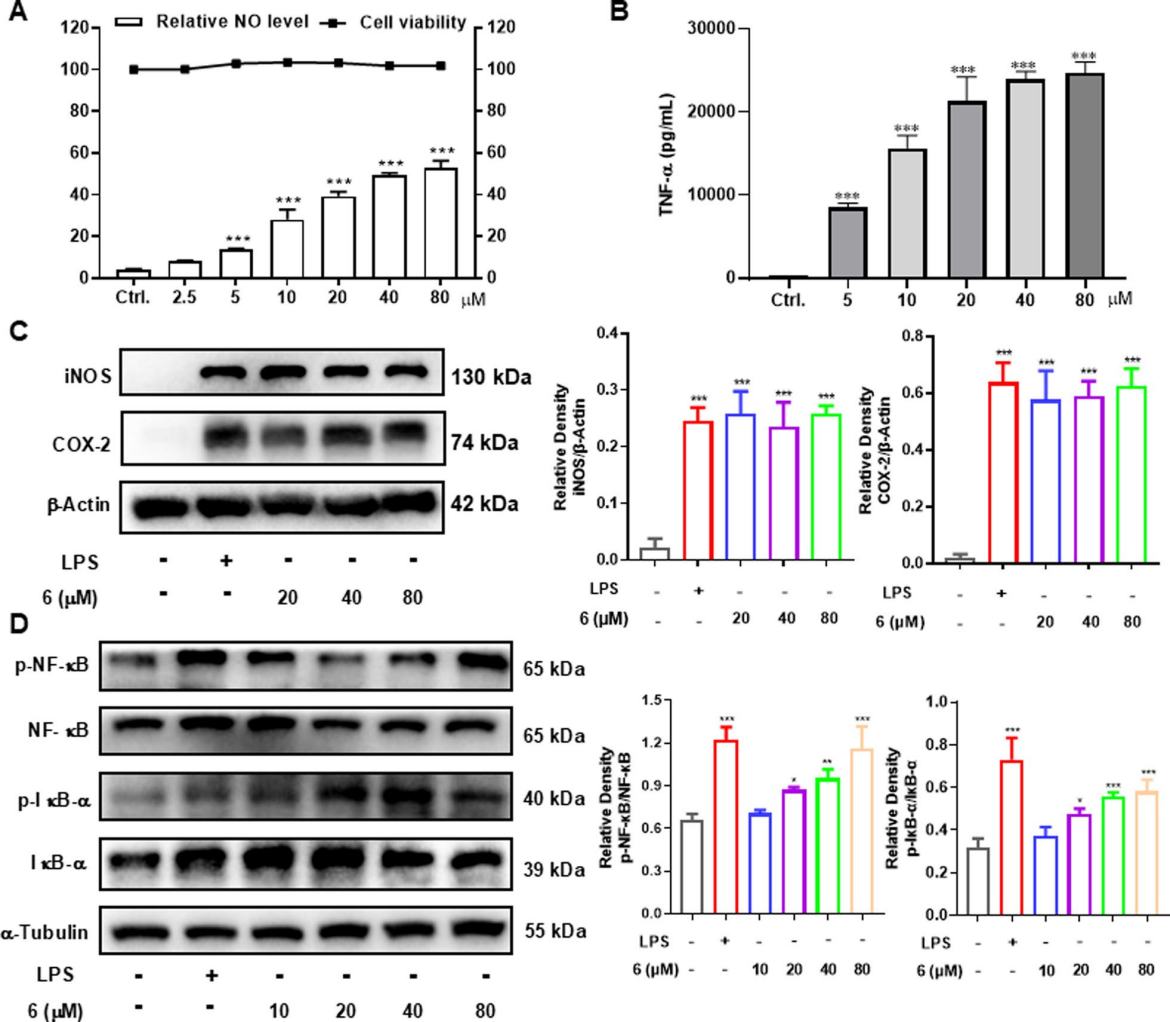
Macrophage immunomodulatory activities of **6**. **A** Relative NO level in the culture supernatant of RAW264.7 cells and cell viability in RAW264.7 cells. **B** Effect of compound **6** on production of TNF-*α* in the RAW264.7 cells. **C** Compound **6** increased the protein levels of iNOS and COX-2 in RAW264.7 cells. **D** Compound **6** increased the protein levels of p-NF-κB and p-IκB-*α* in RAW264.7 cells. The data represent the mean ± SD from three independent experiments. *p < 0.05, **p < 0.01 and ***p < 0.001 compared with the control group

Macrophages, a crucial type of immune cell originating from monocytes, are fundamentally important players in the body’s immune response. Immune cells secrete cytokines (e.g., NO, TNF-*α*) to stimulate the immune response, making these molecules useful quantifiers of cellular activation [29, 30]. In current study, all the isolates were first evaluated for their impact on the cell growth of RAW264.7 macrophages but no significant effect was found on cell survival. Further study on the culture supernatant revealed that several diterpenoids could enhance the NO production in RAW264.7 cells, with LPS (200 ng/mL) used as the comparable drug (Table 1). Selected for further study due to its high activity, compound **6** demonstrated the ability to dose-dependently increase the release of NO (Fig. 7A) and TNF-*α* (Fig. 7B). iNOS, an inducible enzyme that catalyzes NO production from L-arginine, plays a crucial role in the immune system [31], while COX-2 and NO often act synergistically during immune responses [32]. As shown in Fig. 7C, compound **6** effectively upregulated the protein expression of these two enzymes in RAW264.7 cells. NF-κB signaling plays a key role in expressing proteins related to inflammation and immunity, and it has a close relationship with the genes encoding iNOS and COX-2 [33, 34]. Therefore, western blot analysis was employed to determine whether the immunomodulation effect involves the activation of the NF-κB signaling pathway. Results showed that the phosphorylation of NF-κB and IκB-*α* could be activated by **6** in a dose-dependent manner, while the total amounts of NF-κB and IκB-*α* were not obviously altered (Fig. 7D). Thus, the immunomodulatory activity of **6** might related to the activation of NF-κB signaling pathway.

**Table 1:** The enhancement of NO production of compounds **1** − **20**

No	Relative NO level (%)		No	Relative NO level (%) No	
	40 μmol/L	20 μmol/L		40 μmol/L	20 μmol/L
**1**	5.0 ± 0.2	6.1 ± 1.4	**11**	7.4 ± 1.7	10.0 ± 1.0
**2**	14.2 ± 0.9	12.6 ± 0.6	**12**	2.6 ± 2.1	3.7 ± 0.2
**3**	6.3 ± 1.5	10.4 ± 1.4	**13**	5.5 ± 0.6	4.2 ± 0.9
**4**	3.8 ± 0.7	3.3 ± 0.2	**14**	5.6 ± 1.9	10.6 ± 2.1
**5**	12.8 ± 0.1	10.6 ± 0.1	**15**	6.0 ± 2.6	10.2 ± 1.9
**6**	47.2 ± 2.2	38.5 ± 0.6	**16**	10.0 ± 0.7	7.1 ± 1.1
**7**	24.8 ± 1.0	16.2 ± 0.9	**17**	11.2 ± 1.2	11.5 ± 2.3
**8**	1.1 ± 1.5	-0.5 ± 1.6	**18**	12.3 ± 1.6	8.1 ± 0.7
**9**	7.2 ± 2.9	9.9 ± 0.6	**19**	7.6 ± 1.1	8.3 ± 1.0
**10**	1.4 ± 0.3	5.4 ± 0.9	**20**	3.4 ± 1.4	5.5 ± 2.4

Results are expressed as average SD (n = 3)

## Experimental

### Plant materials

The tuberous roots of *Paratinospora sagittata* (Oliv.) Wei Wang were collected in Aug 2018 in Jinghong, Yunnan province, China. A voucher specimen has been deposited at School of Biological Science and Technology, University of Jinan (Accession number: npmc-047).

### Extraction and isolation

The tuberous roots of *P. sagittata* (30 kg) were air-dried and powdered, then extracted with 95% EtOH at room temperature for three consecutive weeks. The EtOH extract was dissolved in water and partitioned with equal volumes of EtOAc and *n*-BuOH using a separating funnel. The *n*-BuOH fraction (760 g) was subjected to chromatography on a D101 macroporous resin column using a gradient of EtOH-water (30%, 50% and 90%) to yield three fractions. The 30% EtOH eluent was further purified by silica gel column chromatography (CC), eluting with a gradient of PE-EtOAc–MeOH (3:1:0 to 0:1:5), resulting in four sub-fractions (A-D). Directed by MS/MS-based molecular networking, diterpenoid-containing fractions B (20 g) and C (56 g) were selected for separation. Fraction B was subjected to silica gel CC and eluted with CH_2_Cl_2_- MeOH (100:1 to 5:1) to obtain three subfractions (B1 − B3). Subfraction B2 was separated by RP-C_18_ CC (MeOH-H_2_O, 2:8 to 8:2) and Sephadex LH-20 CC (MeOH), yielding B2a−B2c. Subsequent semi-preparative HPLC of B2a (61% MeOH-H_2_O) gave **15** (2.3 mg, t_R_ = 11 min) and **10** (3.6 mg, t_R_ = 15.5 min); B2b (59% MeOH-H_2_O) gave **13** (3.7 mg, t_R_ = 18 min), **6** (3.0 mg, t_R_ = 21 min), **5** (8.0 mg, t_R_ = 22.5 min) and **12** (22.3 mg, t_R_ = 29.5 min); B2c (55% MeOH–H₂O) gave **4** (1.2 mg, t_R_ = 12 min), **7** (2.6 mg, t_R_ = 15.5 min) and **3** (3.4 mg, t_R_ = 18 min). Fraction C was subjected to silica gel CC and eluted with PE-EtOAc–MeOH (5:1:0 to 0:1:6) into C1−C3. Subfraction C2 was separated by silica gel CC (CH_2_Cl_2_-MeOH, 100:1 to 5:1) to obtain C2a−C2d. C2b was subjected to RP-C_18_ CC (MeOH-H_2_O, 3:7 to 7:3), yielding C2b1−C2b4. C2b2 purification via Sephadex LH-20 CC (MeOH) and semi-preparative HPLC (48% MeOH-H_2_O) afforded **20** (7.8 mg, t_R_ = 13 min), **16** (3.0 mg, t_R_ = 16 min) and **1** (1.5 mg, t_R_ = 18 min). C2b3 was purified by Sephadex LH-20 CC (MeOH), RP-C_18_ CC (MeOH–H₂O, 3:7 to 10:0), and semi-preparative HPLC (43% MeOH-H_2_O) to yield **14** (3 mg, t_R_ = 8 min), **11** (4.0 mg, t_R_ = 9.5 min) and **9** (6.0 mg, t_R_ = 10.5 min). C2c was purified by Sephadex LH-20 CC (MeOH) and RP-C_18_ CC (MeOH-H_2_O, 3:7 to 10:0), yielding C2c1−C2c5. Semi-preparative HPLC of C2c3 (38% MeCN-H_2_O) gave **2** (1.7 mg, t_R_ = 10.5 min) and **17** (6.0 mg, t_R_ = 11.5 min), while C2c4 (28% MeCN-H_2_O) gave **18** (1.5 mg, t_R_ = 15.5 min), **8** (3.6 mg, t_R_ = 18.5 min) and **19** (0.9 mg, t_R_ = 22.5 min).

#### Tinospinoside F (1)

Colorless gum; $[\alpha]^{25}_{\rm{D}}$ − 63 (*c* 0.34, MeOH); UV (MeOH) *λ*_max_ (log *ε*) 211 (3.88); ECD (*c* 6.5 × 10^–4^ M, MeOH) *λ*_max_ (Δ*ε*)229 (+ 3.91) nm, 260 (+ 2.30) nm; ^1^H and ^13^C NMR data see Tables 2 and 3; HRESIMS (pos.) *m*/*z* 543.1836 [M + Na]^+^ (calcd for C_26_H_32_O_11_Na^+^, 543.1837).

**Table 2:** ^1^H NMR data for compounds **1**−**8** at 600 MHz

No	**1**^*a*^	**2**^*a*^	**3**^*b*^	**4**^*b*^	**5**^*a*^	**6**^*b*^	**7**^*b*^	**8**^*a*^
1	2.41, dd (15.2, 7.7)	1.83, m	1.95, m	1.95, m	1.95, m	1.91, m (2H)	1.92, m (2H)	2.01, m (2H)
	2.17, ddd (15.2, 8.7, 6.6)	1.66, m	1.59, m	1.59, m	1.74, m			
2	4.76, ddd (8.7, 7.7, 3.6)	2.10, dq (14.0, 3.2)	2.35, m	2.36, m	2.47, m	2.41, m	2.40, m	2.41, m (2H)
		1.73, m	2.23, m	2.24, m	2.34, m	2.30, m	2.31, m	
3	6.84, d (3.6)	4.02, dd (3.2, 2.2)	6.49, t (3.4)	6.56, t (3.2)	7.03, t (3.8)	7.01, t (3.5)	7.00, t (3.9)	6.87, t (3.6)
6	4.56, dd (10.6, 7.3)	5.02, dd (12.7, 3.8)	5.29, s	4.45, s	5.41, d (6.1)	5.47, d (6.0)	5.45, d (6.1)	4.68, dd (11.1, 6.9)
7	2.47, ddd (14.0, 7.3, 2.3)	2.36, m	6.99, s	6.90, s	1.99, m	2.19, m	2.15, m	2.27, ddd (14.2, 6.9, 3.4)
	1.43, dd (14.0, 2.7)	2.20, q (12.4)			2.17, m	1.97, d (12.6)	1.96, d (12.5)	1.59, ddd (14.2, 11.1, 4.4)
8	2.90, dd (12.8, 2.3)	2.80, ddd (12.4, 5.1, 1.7)			2.90, m	2.75, brd (5.5)	2.69, brd (5.5)	3.00, dd (4.4, 3.4)
10	2.35, dd (1.6, 6.6)	1.75, d (5.1)	1.80, m	1.80, dd (9.8, 3.5)	1.40, dd (5.0, 2.4)	1.49, m	1.47, d (4.1)	2.13, m
11	2.25, dd (14.6, 4.4)	2.71, m	2.35, dd (14.5, 3.5)	2.36, m	2.26, dd (14.9, 9.8)	2.21, dd (14.8, 10.1)	1.67, dd (15.0, 2.9)	2.34, dd (15.0, 9.8)
	1.95, dd (14.6, 12.2)	1.83, m	2.02, dd (14.5, 11.7)	2.03, m	1.58, dd (14.9, 2.6)	1.67, dd (14.8, 1.6)	2.17, m	1.56, d (15.0)
12	5.52, dd (12.2, 4.4)	5.63, dd (11.9, 4.0)	5.17, dd (11.7, 3.5)	5.17, dd (11.6, 3.3)	5.35, dd (9.8, 2.6)	5.33, dd (10.1, 1.6)	5.25, dd (9.9, 3.0)	5.25, dd (9.8, 1.9)
14	6.53, dd (1.8, 0.7)	6.57, m	6.42, brs	6.42, brs	6.53, brd (1.9)	6.49, brs	6.48, brs	6.44, d (0.9)
15	7.52, t (1.7)	7.52, t (1.8)	7.42, brs	7.42, brs	7.44, t (1.8)	7.40, brs	7.40, m	7.42, dd (1.5, 0.9)
16	7.61, m	7.62, m	7.46, brs	7.46, brs	7.55, d (1.5)	7.49, brs	7.47, m	7.48, d (1.5)
19	1.39, s	1.26, s	1.37, s	1.18, s	1.32, s	1.32, s	1.32, s	1.24, s
20	0.94, s	1.22, s	1.17, s	1.38, s	1.32, s	1.28, s	1.25, s	1.19, s
1′	4.50, d (7.8)	4.30, d (7.8)	4.41, d (7.3)	4.35, d (7.3)	4.28, d (7.8)	4.32, d (7.7)	4.20, d (7.3)	4.15, d (7.7)
2′	3.18, dd (9.2, 7.8)	3.15, dd (8.8, 7.6)	3.38, m	3.38, dd (8.7,7.3)	3.30, m	3.43, t (8.5)	3.31, t (8.1)	3.16, m
3′	3.35, m	3.31, m	3.56, m	3.50, dd (8.7, 8.3)	4.81, m	3.65, t (9.2)	3.42, m	3.23, m
4′	3.28, m	3.29, m	3.37, m	3.73, m	3.38, m	4.83, t (9.5)	3.66, m	3.20, m
5′	3.33, m	3.23, m	3.56, m	4.02, dd (11.5, 4.9)	3.24, m	3.33, m	3.95, dd (11.6, 5.2)	3.13, m
				3.28, dd (11.5, 10.2)			3.17, m	
6′	3.90, dd (11.9, 2.2)	3.85, dd (11.9, 2.3)	4.39, brd (12.0)		3.87, dd (11.8, 2.1)	3.71, m		3.88, dd (11.8, 2.3)
	3.67, dd (11.9, 5.9)	3.68, dd (11.9, 5.4)	4.31, dd (12.0, 6.7)		3.64, dd (11.8, 5.9)	3.56, dd (12.4, 3.8)		3.64, dd (11.8, 6.3)
OCH_3_			3.74, s	3.75, s	3.72, s	3.73, s	3.73, s	
OAc			2.17, s		2.09, s	2.13, s		

^*a*^in CD_3_OD

^*b*^in CDCl_3_

**Table 3:** ^13^C NMR data for compounds **1**−**8** at 150 MHz

No	**1**^*a*^	**2**^*a*^	**3**^*b*^	**4**^*b*^	**5**^*a*^	**6**^*b*^	**7**^*b*^	**8**^*a*^
1	27.1	20.2	20.4	20.4	17.4	16.4	16.6	18.4
2	74.2	28.0	24.8	24.9	25.2	24.2	24.3	25.8
3	136.7	79.9	136.7	137.2	143.9	142.4	142.5	138.6
4	137.5	82.0	138.4	138.1	135.5	134.2	134.5	135.1
5	41.1	46.8	41.6	41.7	40.4	39.2	39.4	40.2
6	85.8	77.2	82.7	82.1	84.5	82.9	82.9	85.5
7	27.9	27.6	136.2	137.4	30.3	29.5	29.7	29.7
8	43.3	47.9	136.7	135.9	47.9	46.5	46.6	47.9
9	37.7	37.5	37.0	37.0	40.6	39.3	39.4	39.7
10	53.8	48.0	47.8	47.9	47.2	45.6	45.7	43.8
11	46.4	40.9	45.1	45.1	48.0	46.9	46.8	48.8
12	71.6	73.9	70.6	70.6	69.5	69.0	68.7	68.8
13	125.4	126.8	123.7	123.7	127.6	125.8	125.9	127.9
14	109.7	109.5	108.6	108.6	110.3	108.8	108.9	110.0
15	145.2	145.2	143.7	143.7	144.5	143.7	143.8	144.5
16	141.5	141.4	139.8	139.8	141.6	140.1	140.3	141.5
17	175.8	175.1	168.7	168.8	180.6	178.5	178.3	177.9
18	171.0	180.6	171.6	171.1	168.3	166.8	167.0	172.3
19	30.2	20.6	22.3	28.1	27.6	27.1	27.3	30.0
20	22.7	24.4	27.9	21.9	22.0	21.8	21.8	22.2
1′	104.1	105.0	105.0	105.5	100.6	98.7	99.4	100.6
2′	75.1	75.1	70.0	73.7	73.6	74.4	73.8	75.3
3′	78.1	77.8	76.1	76.4	79.1	74.4	76.5	78.0
4′	71.8	71.4	74.0	69.4	70.2	70.9	70.0	72.0
5′	78.0	78.0	73.8	65.5	77.7	74.1	65.5	77.8
6′	62.8	62.6	63.7		62.7	61.4		63.2
OMe			52.4	52.3	52.1	51.7	51.8	
OAc			172.0		172.7	171.6		
			20.8		21.1	20.9		

^*a*^in CD_3_OD

^*b*^in CDCl_3_

#### Tinospinoside G (2)

Colorless gum; $[\alpha]^{25}_{\rm{D}}$ + 26 (*c* 0.09, MeOH); UV (MeOH) *λ*_max_ (log *ε*) 209 (4.14); ECD (*c* 17 × 10^–4^ M, MeOH) *λ*_max_ (Δ*ε*) 224 (+ 0.10) nm, 238 (− 0.21) nm, 251 (− 0.08) nm; ^1^H and ^13^C NMR data see Tables 2 and 3; HRESIMS (pos.) *m*/*z* 556.2393 [M + NH_4_]^+^ (calcd for C_26_H_38_O_12_N^+^, 556.2389).

#### Tinospinoside H (3)

Colorless gum; $[\alpha]^{25}_{\rm{D}}$ + 186 (*c* 0.20, MeOH); UV (MeOH) *λ*_max_ (log *ε*) 207 (3.74); ECD (*c* 2.6 × 10^–4^ M, MeOH) *λ*_max_ (Δ*ε*) 210 (+ 3.03) nm, 226 (+ 39.40) nm, 252 (− 10.14) nm; ^1^H and ^13^C NMR data see Tables 2 and 3; HRESIMS (pos.) *m*/*z* 577.2279 [M + H]^+^ (calcd for C_29_H_33_O_12_^+^, 577.2280).

#### Tinospinoside I (4)

Yellow gum; $[\alpha]^{25}_{\rm{D}}$ + 122 (*c* 0.60, MeOH); UV (MeOH) *λ*_max_ (log *ε*) 212 (4.25); ECD (*c* 3.0 × 10^–4^ M, MeOH) *λ*_max_ (Δ*ε*) 209 (− 3.20) nm, 227 (+ 36.60) nm, 251 (− 11.66) nm; ^1^H and ^13^C NMR data see Tables 2 and 3; HRESIMS (pos.) *m*/*z* 527.1889 [M + Na]^+^ (calcd for C_26_H_32_O_10_Na^+^, 527.1888).

#### Tinospinoside J (5)

Yellow gum; $[\alpha]^{25}_{\rm{D}}$ − 40 (*c* 0.55, MeOH); UV (MeOH) *λ*_max_ (log *ε*) 214 (3.76); ECD (*c* 4.8 × 10^–4^ M, MeOH) *λ*_max_ (Δ*ε*) 218 (+ 4.01), 247 (− 3.79) nm; ^1^H and ^13^C NMR data see Tables 2 and 3; HRESIMS (pos.) *m*/*z* 601.2253 [M + Na]^+^ (calcd for C_29_H_38_O_12_Na^+^, 601.2255).

#### Tinospinoside K (6)

Yellow gum; $[\alpha]^{25}_{\rm{D}}$ − 50 (*c* 0.25, MeOH); UV (MeOH) *λ*_max_ (log *ε*) 215 (3.92); ECD (*c* 3.5 × 10^–4^ M, MeOH) *λ*_max_ (Δ*ε*) 221 (+ 5.27) nm, 246 (− 5.00) nm; ^1^H and ^13^C NMR data see Tables 2 and 3; HRESIMS (pos.) *m*/*z* 601.2258 [M + Na]^+^ (calcd for C_29_H_38_O_12_Na^+^, 601.2255).

#### Tinospinoside L (7)

Colorless gum; $[\alpha]^{25}_{\rm{D}}$ − 20 (*c* 0.10, MeOH); UV (MeOH) *λ*_max_ (log *ε*) 211 (4.10); ECD (*c* 0.97 × 10^–4^ M, MeOH) *λ*_max_ (Δ*ε*) 207 (− 0.67) nm, 222 (+ 6.93) nm, 250(− 5.74) nm; ^1^H and ^13^C NMR data see Tables 2 and 3; HRESIMS (pos.) *m*/*z* 529.2041 [M + Na]^+^ (calcd for C_26_H_34_O_10_Na^+^, 529.2044).

#### Tinospinoside M (8)

White gum; $[\alpha]^{25}_{\rm{D}}$ + 82 (*c* 0.69, MeOH); UV (MeOH) *λ*_max_ (log *ε*) 210 (3.95); ECD (*c* 3.2 × 10^–4^ M, MeOH) *λ*_max_ (Δ*ε*) 209 (− 6.71) nm, 252 (+ 3.69) nm; ^1^H and ^13^C NMR data see Tables 2 and 3; HRESIMS (pos.) *m*/*z* 545.1993 [M + Na]^+^ (calcd for C_26_H_34_O_11_Na^+^, 545.1993).

### LC–MS/MS and molecular networking analysis

Details were the same as we formerly described [18].

### Absolute configuration determination of the sugar unit in 1−8

Compounds **1**−**8** bear a *β*-glucose/xylose moiety whose absolute configurations were determined by chemical derivatization as we reported before [35], and compounds **1** and **7** were chosen as the model molecule.

### ECD Calculations

The ECD calculations of compounds **1**−**3** and **8** were performed according to the previously described method [36].

### NO production and cell viability assays

RAW264.7 macrophages (5 × 10^4^ cells) were cultured at 37 °C in 96-well flat plates, in the presence or absence of various concentrations of compound **6** for 24 h, in a humidified and 5% CO_2_-containing incubator. The nitrite accumulation in the culture medium was measured using Griess reagent at 540 nm on the microplate reader. The relative NO level was calculated according to the following formula: [1- (A_sample_ − A_blank_)/(A_LPS_ − A_blank_)] × 100%. While the viability of the cultured cells was measured by CCK-8 method. 10 *μ*L CCK-8 was added to each well at the final 2–4 h of culture. To the end of the culture, we measured the OD values with microplate reader at 450 nm.

### Measurement of immune cytokine

TNF-*α* concentrations in culture medium of RAW264.7 macrophages pretreated with presence or absence of various concentrations of compound **6** for 24 h, were measured using commercial ELISA kits following manufacturer protocols. The concentration of TNF-*α* was calculated from 450 nm OD values using standard curves.

### Western blot analysis

Cells in 6-well plates were treated with varying concentrations of compound **6** for 24 h and LPS (200 ng/mL) is used as a reference standard in current experiment, then lysed in extraction buffer containing protease inhibitors; protein concentrations were determined by BCA assay. Equal protein amounts underwent 10% SDS-PAGE followed by PVDF membrane transfer. After blocking with 5% skim milk, membranes were incubated overnight at 4 °C with primary antibodies (iNOS, COX-2, phospho-NF-κB, NF-κB, phospho-IκB-α, IκB-α), washed thrice with TBST, incubated 1 h at room temperature with HRP-conjugated secondary antibody (1:3000), washed again thrice with TBST, and finally detected using ECL on a Chemi-Doc XRS system.

## Conclusion

Plants of the genus *Paratinospora* have long been used in Asian traditional medicine to treat immune-related diseases. This study focused on the previously unexplored *n*-BuOH partition of *P. sagittata* extract. Guided by MS/MS-based molecular networking, we isolated and identified eight previously undescribed clerodane diterpenoid glycosides and 12 known analogues. Comprehensive spectroscopic analysis elucidated the novel structures, with absolute configurations confirmed by single-crystal X-ray diffraction, TD-DFT/ECD calculations, and chemical derivatization. Selected diterpenoids modulated macrophage activity by enhancing NO production, potentially through NF-κB pathway activation. The current study has confirmed that the clerodane diterpenoids, which are the main chemical constituents of the *n*-BuOH soluble part of its EtOH extract of *P. sagittata*, exhibit immunomodulatory activity. These findings demonstrate immunomodulatory potential of *P. sagittata*, aligning with the traditional uses of *Paratinospora* species, suggesting that clerodanes may be the pharmacologically active substances responsible for the immune-regulating effects of *Paratinospora* species. However, due to the limited number of compounds investigated thus far, the structure–activity relationship (SAR) regarding how this structural class promotes macrophage function remains unclear. Further studies with a larger sample size are needed to fully elucidate the SAR.

## Supplementary Information

Additional file 1: Original spectroscopic data including NMR and HRESIMS spectra for compounds 1–8.

## Data Availability

The data supporting the findings of this study were available on request from the corresponding author, upon reasonable request.
