# Supplementary material for: Clerodane diterpenoid glycosides from the tuberous roots of Paratinospora sagittata: targeted isolation, structure characterization and immunomodulatory properties
Source: Nat Prod Bioprospect. 2026 Feb 2;16(1):23. doi: 10.1007/s13659-025-00555-2 (PMC12862036; doi:10.1007/s13659-025-00555-2)
Supplement: Supplementary file 1 — Additional file 1: Original spectroscopic data including NMR and HRESIMS spectra for compounds 1–8. [file 13659_2025_555_MOESM1_ESM.docx]

**Supporting information for**

Clerodane diterpenoid glycosides from the tuberous roots of *Paratinospora sagittata*: targeted isolation, structure characterization and immunomodulatory properties

Jun-Sheng Zhang, Rui Ao, Yin-Bo Pan, Xin-Cheng Zhuang, Yi-Ke Yin, Jie-Bao, Hua Zhang*

* Correspondence: Prof. Hua Zhang; Email: [bio_zhangh@ujn.edu.cn](mailto:bio_zhangh@ujn.edu.cn)

**Table of Contents**

[Figure S1. The ^1^H NMR spectrum of 1 in CD_3_OD. 1](#_Toc208502456)

[Figure S2. The ^13^C NMR spectrum of 1 in CD_3_OD. 1](#_Toc208502457)

[Figure S3. The ^1^H-^1^H COSY spectrum of 1 in CD_3_OD 2](#_Toc208502458)

[Figure S4. The HSQC spectrum of 1 in CD_3_OD. 2](#_Toc208502459)

[Figure S5. The HMBC spectrum of 1 in CD_3_OD. 3](#_Toc208502460)

[Figure S6. The NOESY spectrum of 1 in CD_3_OD. 3](#_Toc208502461)

[Figure S7. The (+)-HR-ESIMS spectrum of 1. 4](#_Toc208502462)

[Figure S8. The ^1^H NMR spectrum of 2 in CD_3_OD. 4](#_Toc208502463)

[Figure S9. The ^13^C NMR spectrum of 2 in CD_3_OD. 5](#_Toc208502464)

[Figure S10. The ^1^H-^1^H COSY spectrum of 2 in CD_3_OD. 5](#_Toc208502465)

[Figure S11. The HSQC spectrum of 2 in CD_3_OD. 6](#_Toc208502466)

[Figure S12. The HMBC spectrum of 2 in CD_3_OD. 6](#_Toc208502467)

[Figure S13. The NOESY spectrum of 2 in CD_3_OD 7](#_Toc208502468)

[Figure S14. The (+)-HR-ESIMS spectrum of 2. 7](#_Toc208502469)

[Figure S15. The ^1^H NMR spectrum of 3 in CDCl_3_. 8](#_Toc208502470)

[Figure S16. The ^13^C NMR spectrum of 3 in CDCl_3_. 8](#_Toc208502471)

[Figure S17. The ^1^H-^1^H COSY spectrum of 3 in CDCl_3_. 9](#_Toc208502472)

[Figure S18. The HSQC spectrum of 3 in CDCl_3_. 9](#_Toc208502473)

[Figure S19. The HMBC spectrum of 3 in 10](#_Toc208502474)

[Figure S20. The NOESY spectrum of 3 in CDCl_3_. 10](#_Toc208502475)

[Figure S21. The (+)-HR-ESIMS spectrum of 3. 11](#_Toc208502476)

[Figure S22. The ^1^H NMR spectrum of 4 in CDCl_3_. 11](#_Toc208502477)

[Figure S23. The ^13^C NMR spectrum of 4 in CDCl_3_. 12](#_Toc208502478)

[Figure S24. The ^1^H-^1^H COSY spectrum of 4 in CDCl_3_. 12](#_Toc208502479)

[Figure S25. The HSQC spectrum of 4 in CDCl_3_. 13](#_Toc208502480)

[Figure S26. The HMBC spectrum of 4 in CDCl_3_. 13](#_Toc208502481)

[Figure S27. The NOESY spectrum of 4 in CDCl_3_. 14](#_Toc208502482)

[Figure S28. The (–)-HR-ESIMS spectrum of 4. 14](#_Toc208502483)

[Figure S29. The ^1^H NMR spectrum of 5 in CD_3_OD. 15](#_Toc208502484)

[Figure S30. The ^13^C NMR spectrum of 5 in CD_3_OD. 15](#_Toc208502485)

[Figure S31. The ^1^H-^1^H COSY spectrum of 5 in CD_3_OD. 16](#_Toc208502486)

[Figure S32. The HSQC spectrum of 5 in CD_3_OD. 16](#_Toc208502487)

[Figure S33. The HMBC spectrum 5 in CD_3_OD. 17](#_Toc208502488)

[Figure S34. The NOESY spectrum of 5 in CD_3_OD. 17](#_Toc208502489)

[Figure S35. The (+)-HR-ESIMS spectrum of 5. 18](#_Toc208502490)

[Figure S36. The ^1^H NMR spectrum of 6 in CDCl_3_. 18](#_Toc208502491)

[Figure S37. The ^13^C NMR spectrum of 6 in CDCl_3_. 19](#_Toc208502492)

[Figure S38. The ^1^H-^1^H COSY spectrum of 6 in CDCl_3_. 19](#_Toc208502493)

[Figure S39. The HSQC spectrum of 6 in CDCl_3_. 20](#_Toc208502494)

[Figure S40. The HMBC spectrum 6 in CDCl_3_. 20](#_Toc208502495)

[Figure S41. The NOESY spectrum of 6 in CDCl_3_. 21](#_Toc208502496)

[Figure S42. The (+)-HR-ESIMS spectrum of 6. 21](#_Toc208502497)

[Figure S43. The ^1^H NMR spectrum of 7 in CDCl_3_. 22](#_Toc208502498)

[Figure S44. The ^13^C NMR spectrum of 7 in CDCl_3_. 22](#_Toc208502499)

[Figure S45. The ^1^H-^1^H COSY spectrum of 7 in CDCl_3_. 23](#_Toc208502500)

[Figure S46. The HSQC spectrum of 7 in CDCl_3_. 23](#_Toc208502501)

[Figure S47. The HMBC spectrum 7 in CDCl_3_. 24](#_Toc208502502)

[Figure S48. The NOESY spectrum of 7 in CDCl_3_. 24](#_Toc208502503)

[Figure S49. The (+)-HR-ESIMS spectrum of 7. 25](#_Toc208502504)

[Figure S50. The ^1^H NMR spectrum of 8 in CD_3_OD. 25](#_Toc208502505)

[Figure S51. The ^13^C NMR spectrum of 8 in CD_3_OD. 26](#_Toc208502506)

[Figure S52. The ^1^H-^1^H COSY spectrum of 8 in CD_3_OD. 26](#_Toc208502507)

[Figure S53. The HSQC spectrum of 8 in CD_3_OD. 27](#_Toc208502508)

[Figure S54. The HMBC spectrum 8 in CD_3_OD. 27](#_Toc208502509)

[Figure S55. The NOESY spectrum of 8 in CD3OD. 28](#_Toc208502510)

[Figure S56. The (+)-HR-ESIMS spectrum of 8. 28](#_Toc208502511)

[Figure S57 Subnetwork of tandem MS/MS molecular working for crude extracts of *n*-BuOH part of *T. sadittata*. 29](#_Toc208502512)

[General experimental procedures 30](#_Toc208502513)

[Table S1 Details of crystal data of 12 31](#_Toc208502514)

Figure S1. The ^1^H NMR spectrum of 1 in CD_3_OD.


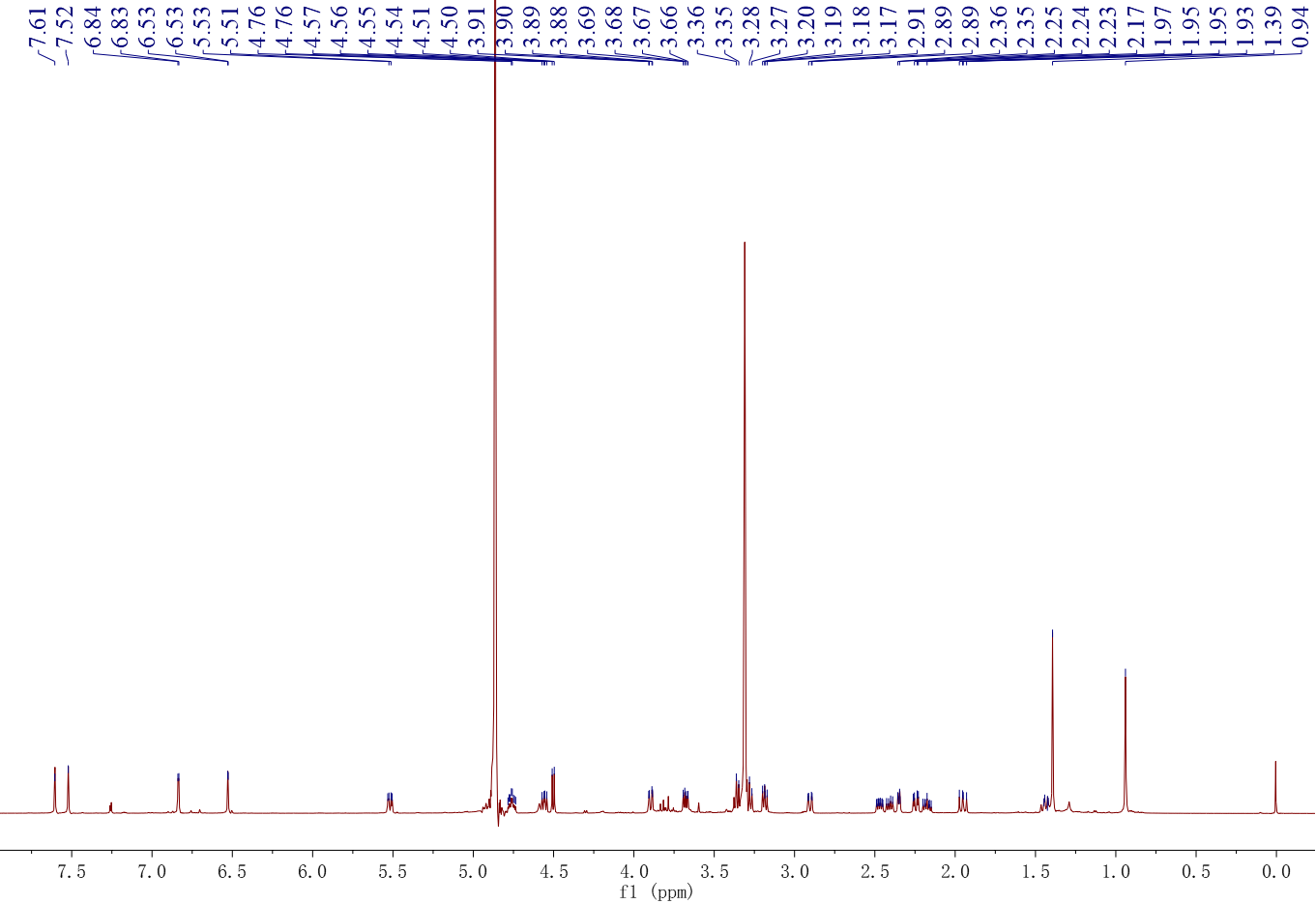


Figure S2. The ^13^C NMR spectrum of 1 in CD_3_OD.


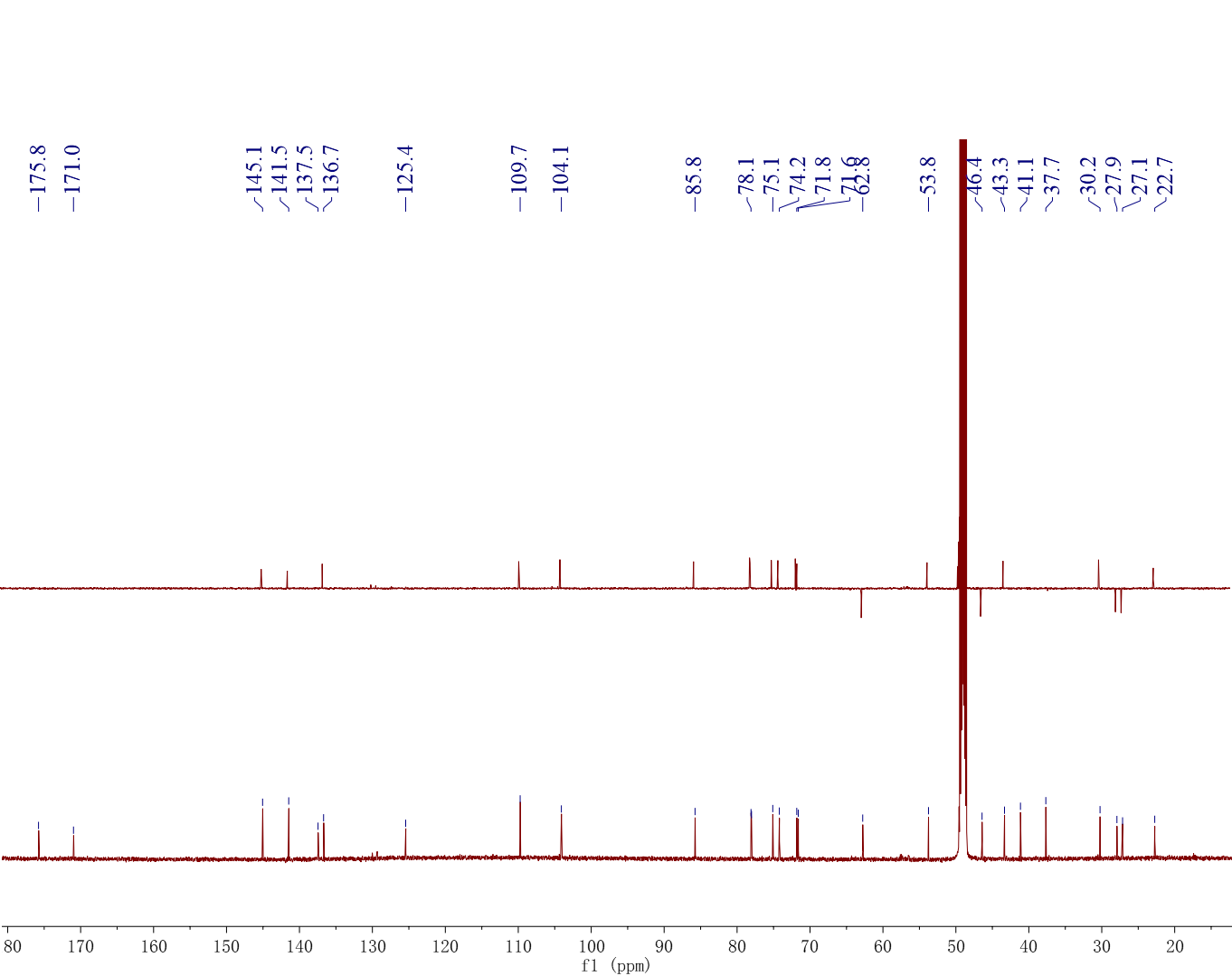


Figure S3. The ^1^H-^1^H COSY spectrum of 1 in CD_3_OD.


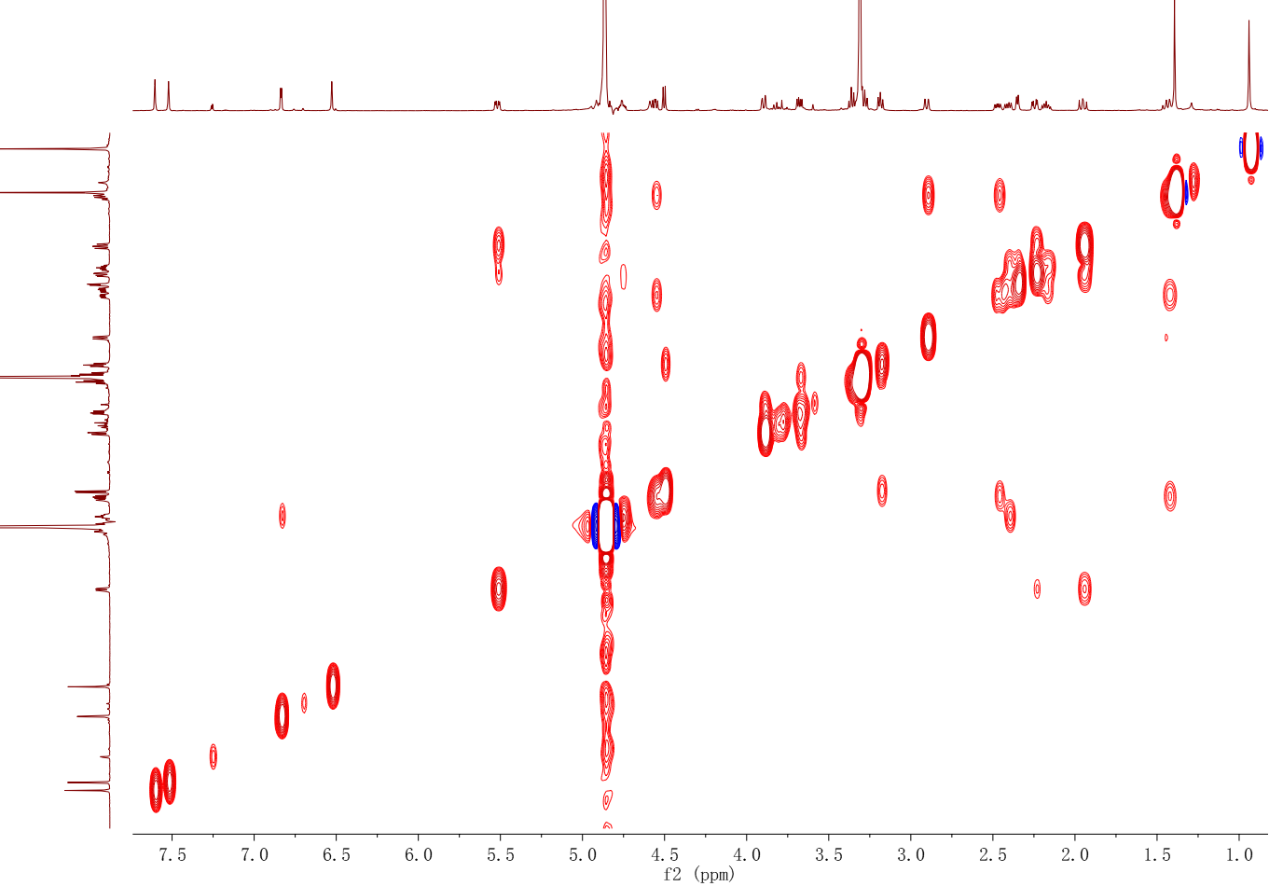


Figure S4. The HSQC spectrum of 1 in CD_3_OD.


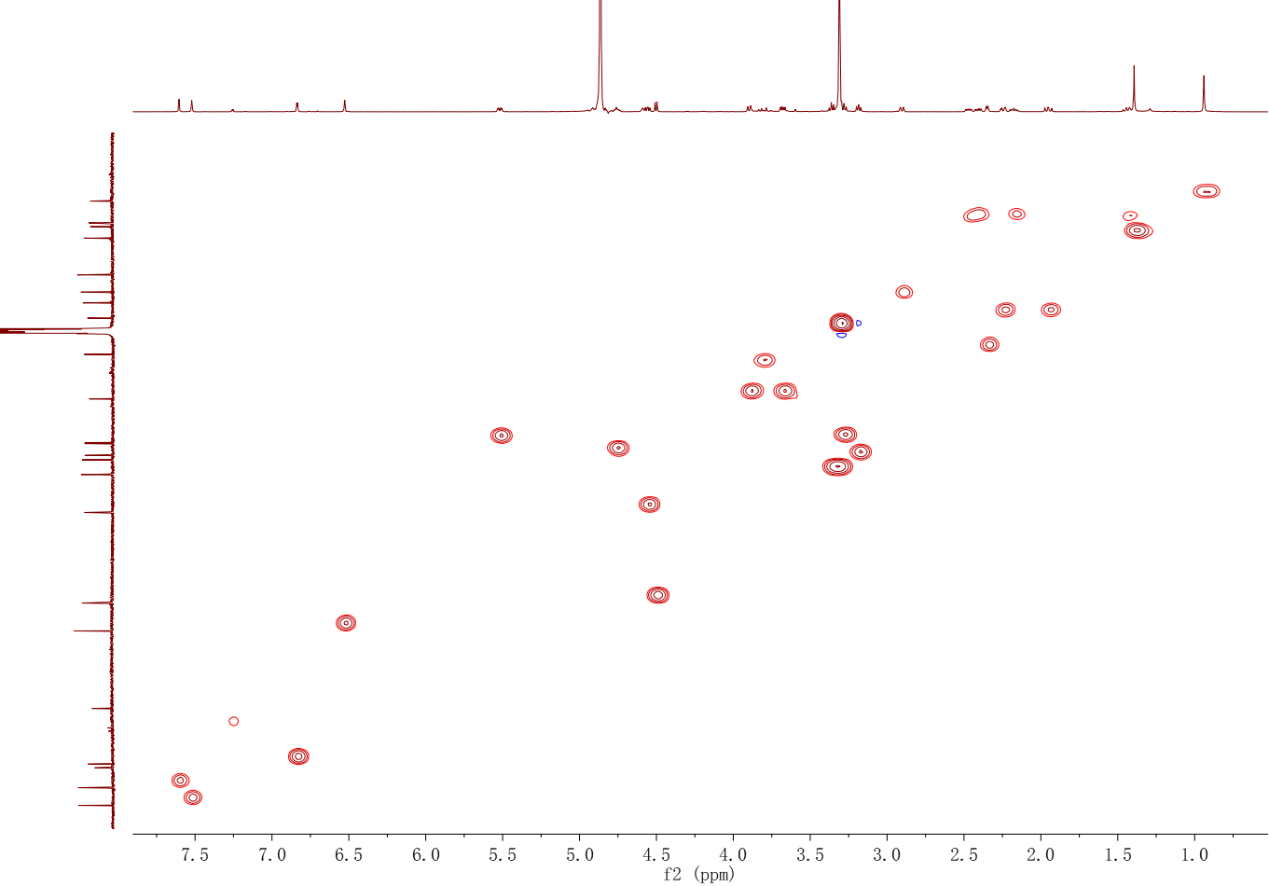


Figure S5. The HMBC spectrum of 1 in CD_3_OD.


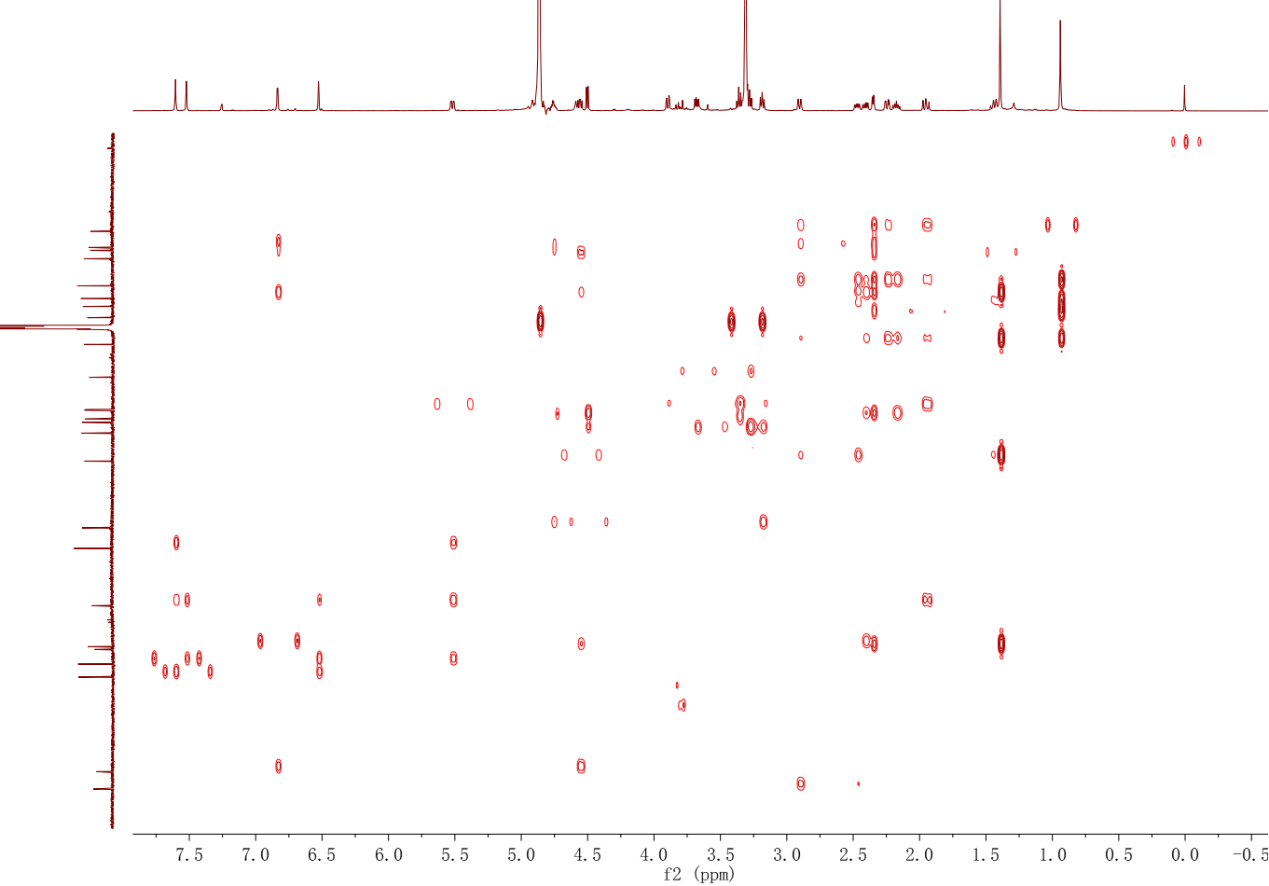


Figure S6. The NOESY spectrum of 1 in CD_3_OD.


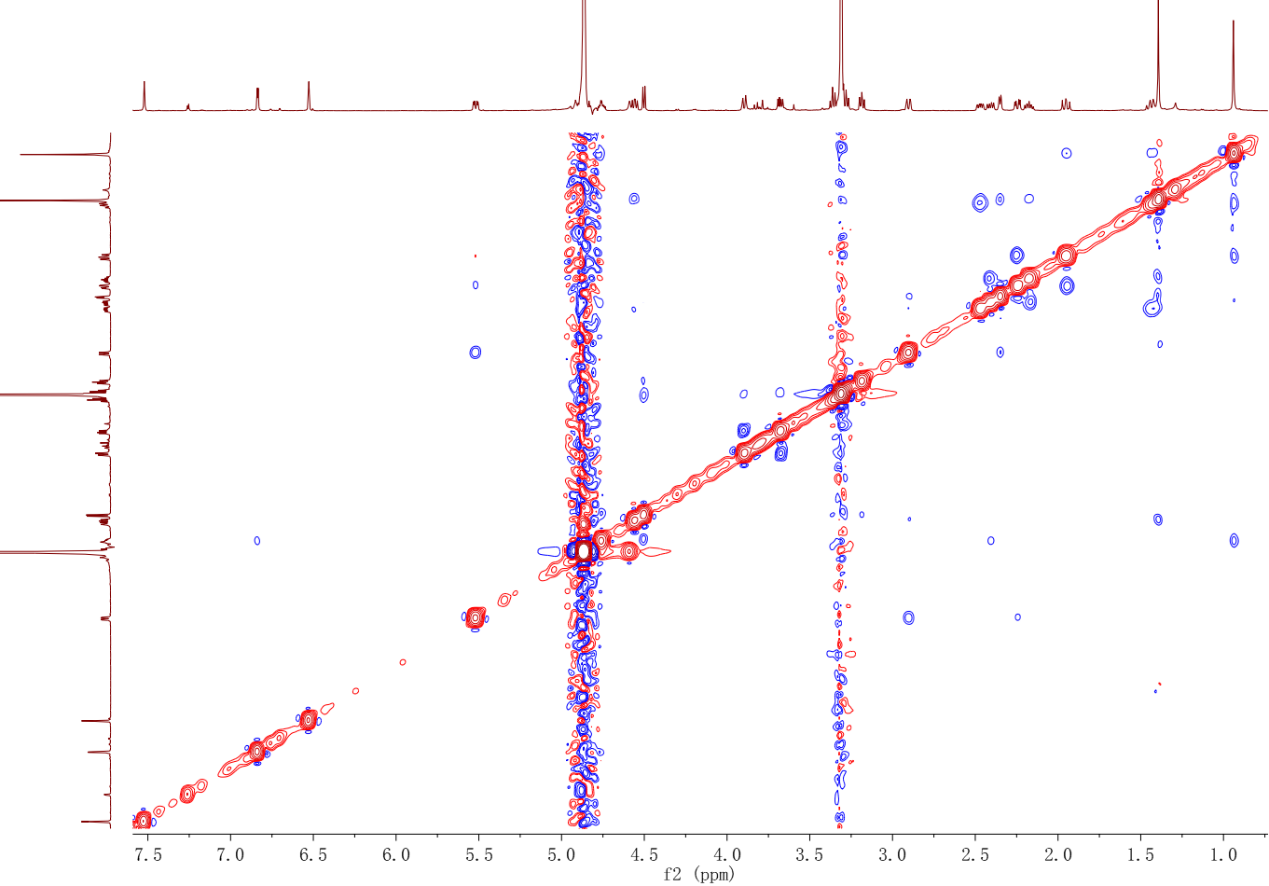


Figure S7. The (+)-HR-ESIMS spectrum of 1.

Figure S8. The ^1^H NMR spectrum of 2 in CD_3_OD.


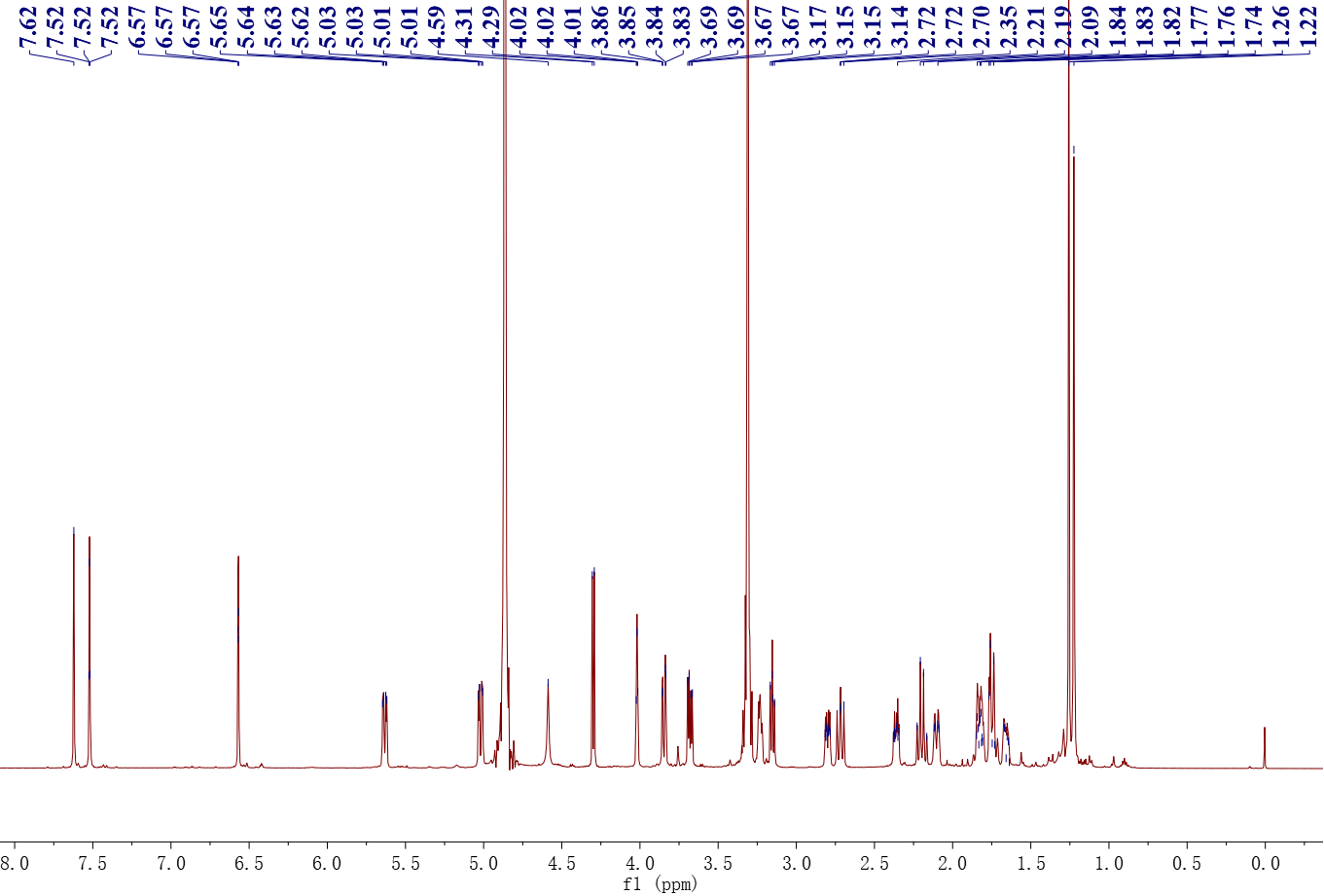


Figure S9. The ^13^C NMR spectrum of 2 in CD_3_OD.


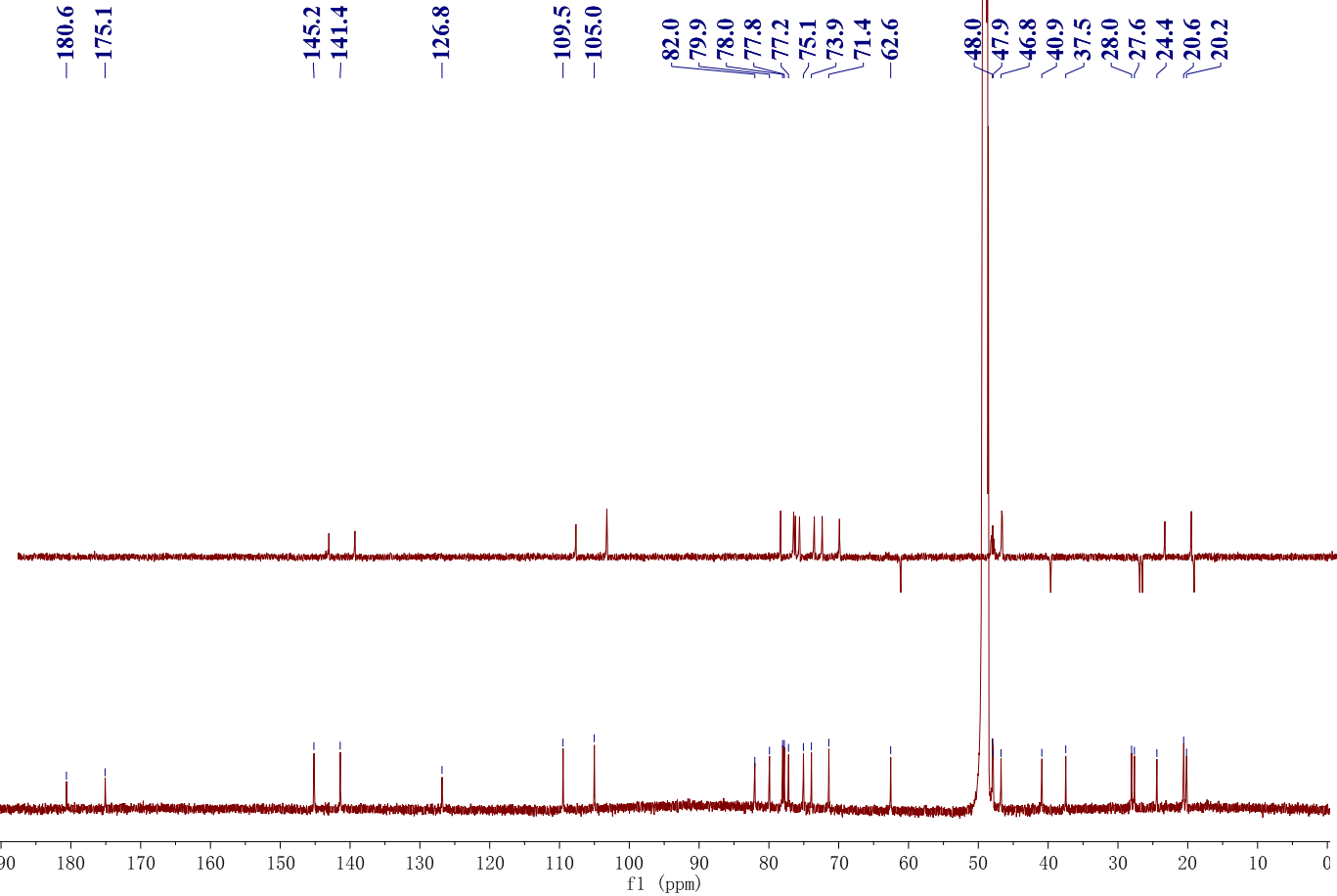


Figure S10. The ^1^H-^1^H COSY spectrum of 2 in CD_3_OD.


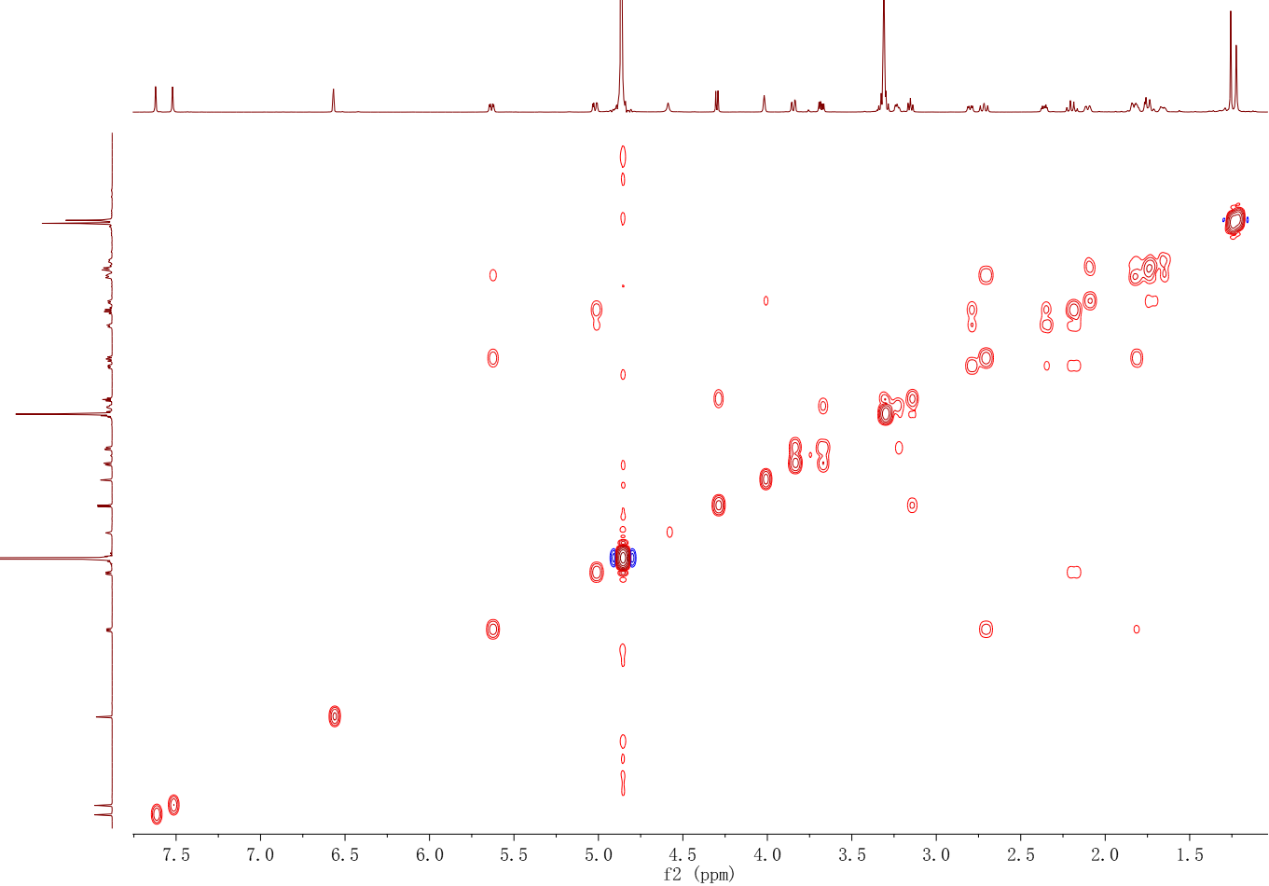


Figure S11. The HSQC spectrum of 2 in CD_3_OD.


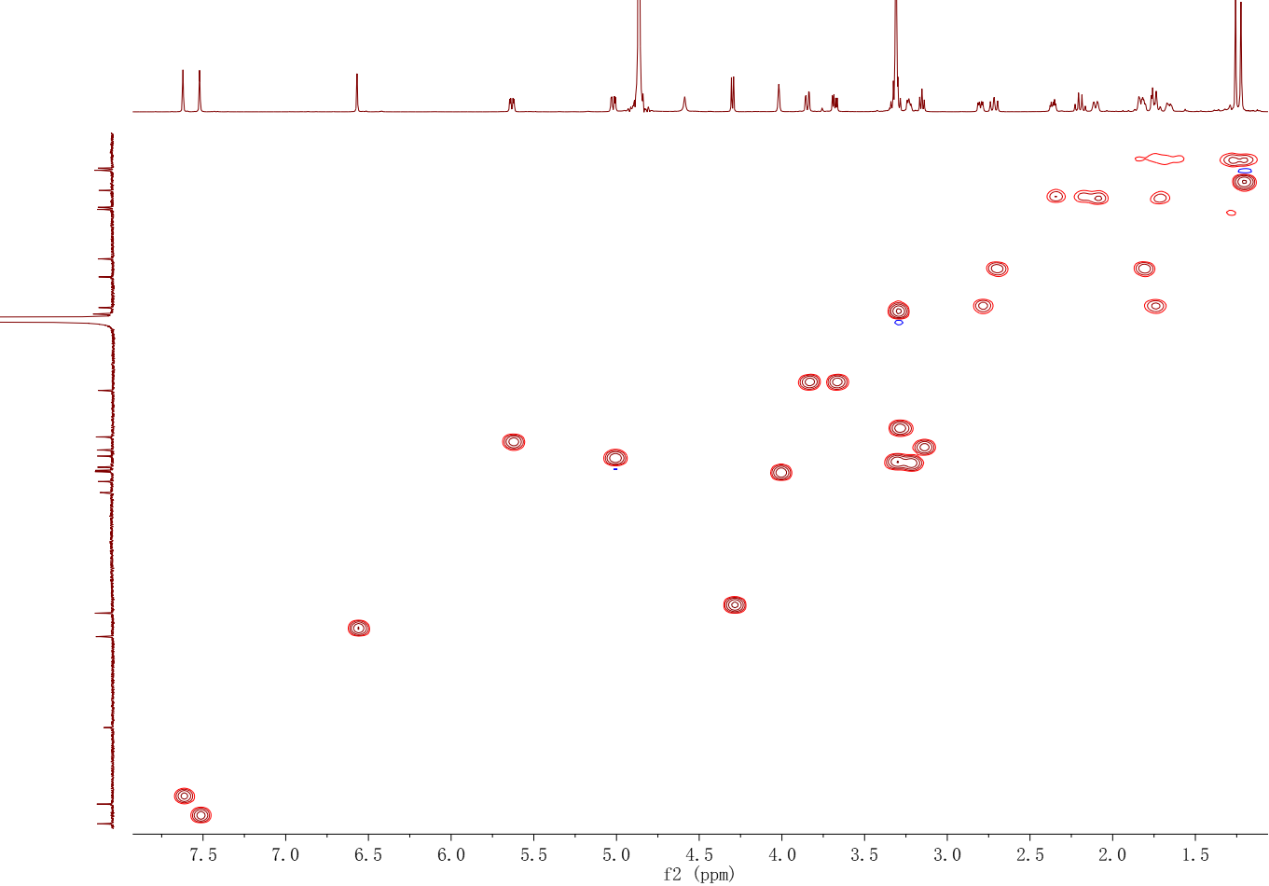


Figure S12. The HMBC spectrum of 2 in CD_3_OD.


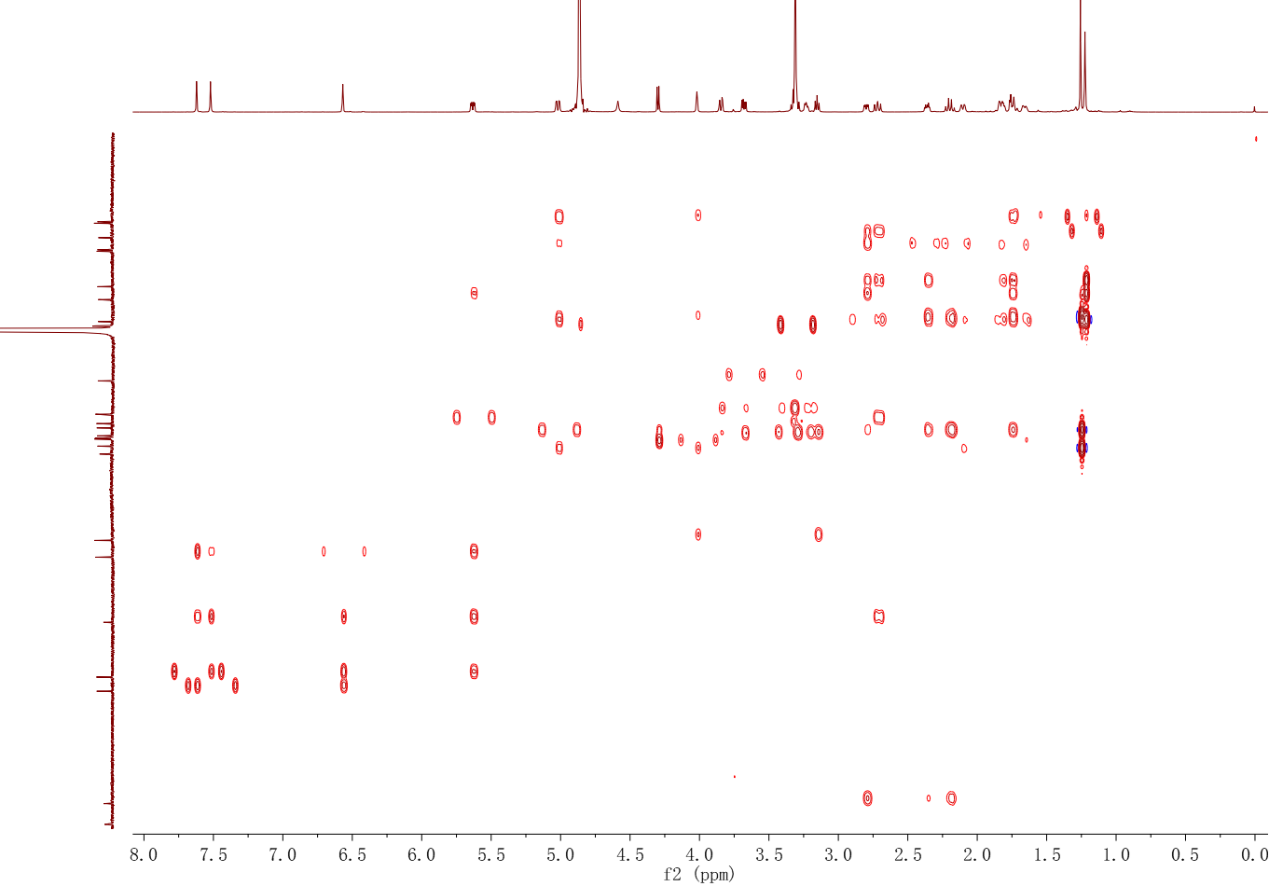


Figure S13. The NOESY spectrum of 2 in CD_3_OD


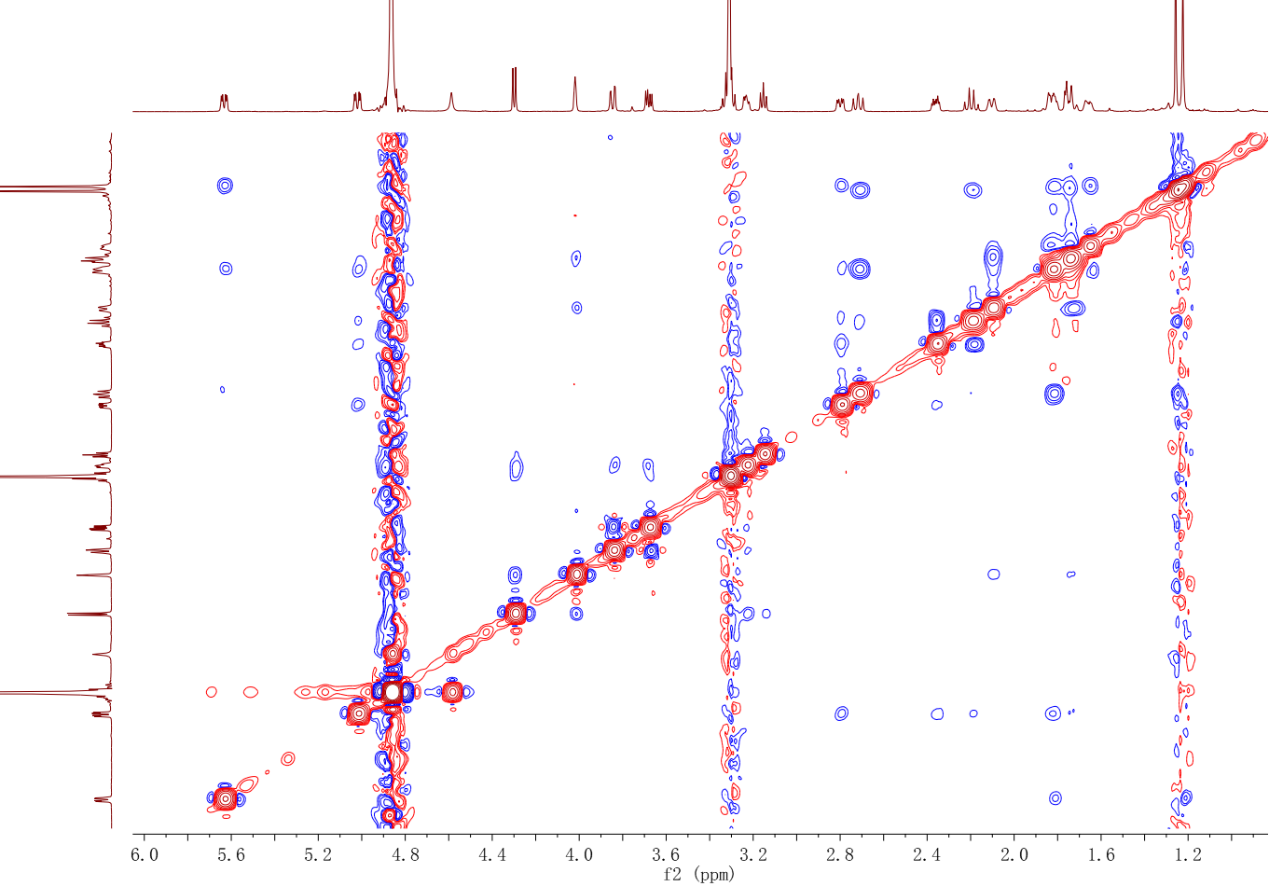


Figure S14. The (+)-HR-ESIMS spectrum of 2.

Figure S15. The ^1^H NMR spectrum of 3 in CDCl_3_.


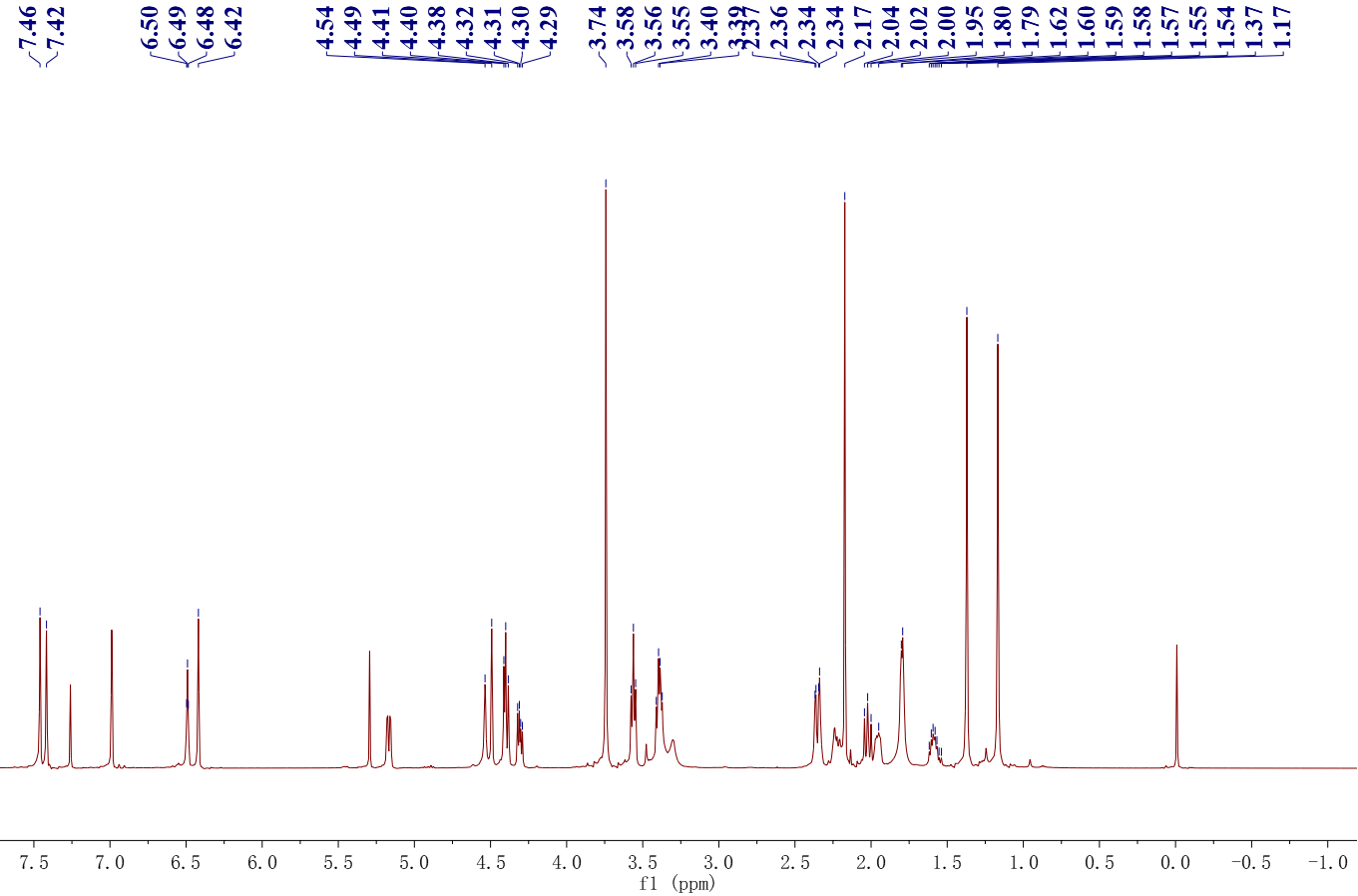


Figure S16. The ^13^C NMR spectrum of 3 in CDCl_3_.


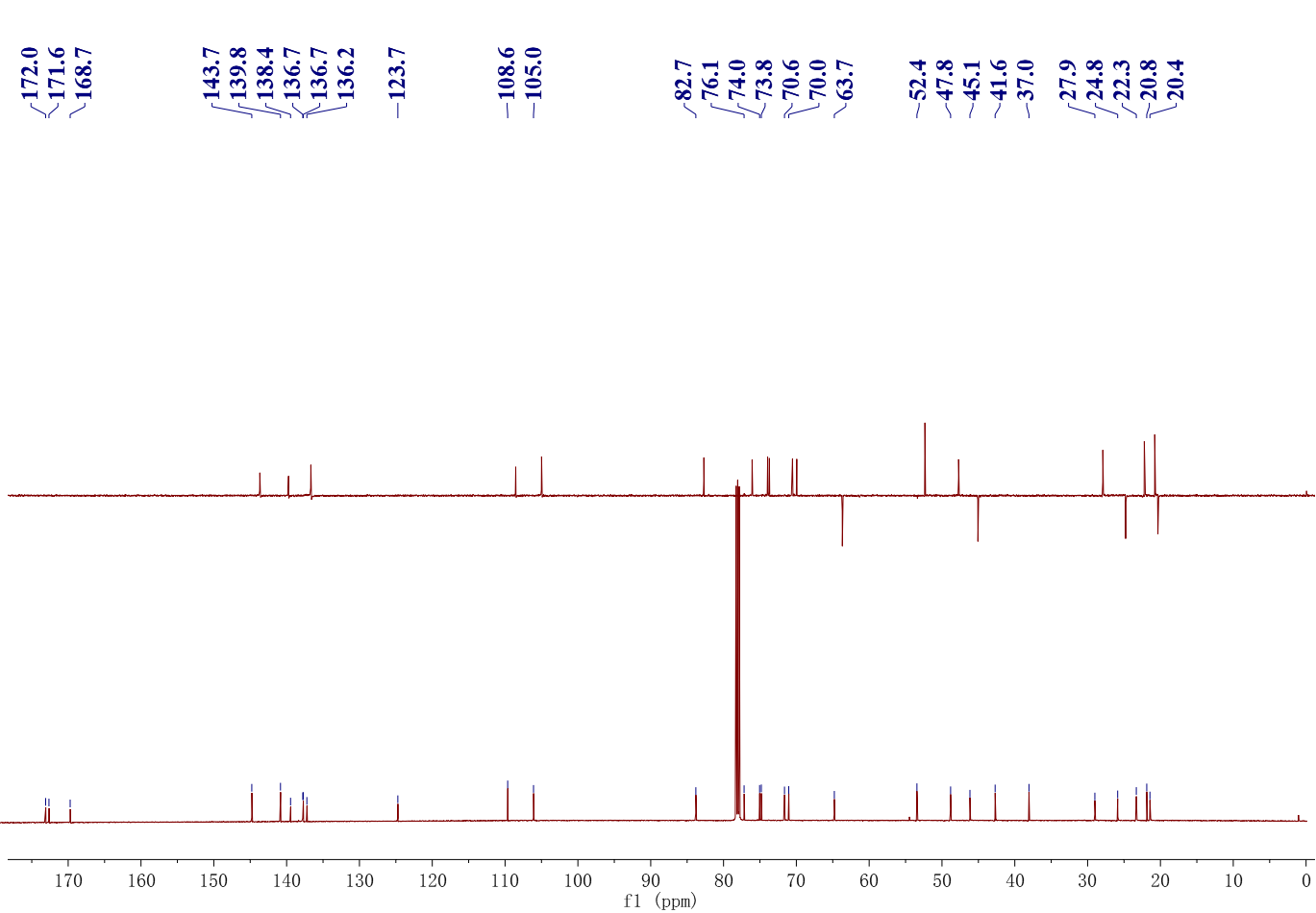


Figure S17. The ^1^H-^1^H COSY spectrum of 3 in CDCl_3_.

Figure S18. The HSQC spectrum of 3 in CDCl_3_.


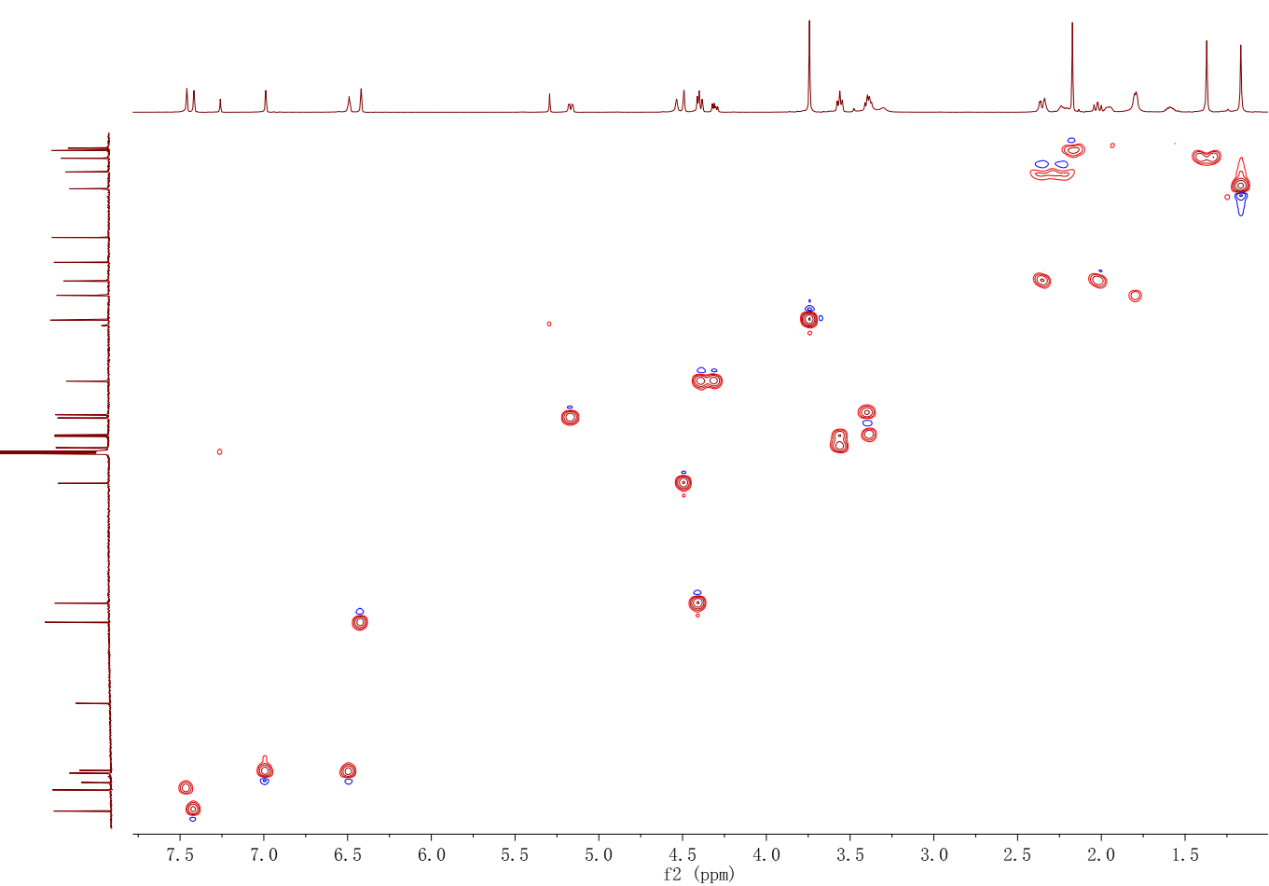


Figure S19. The HMBC spectrum of 3 in **CDCl_3_**.


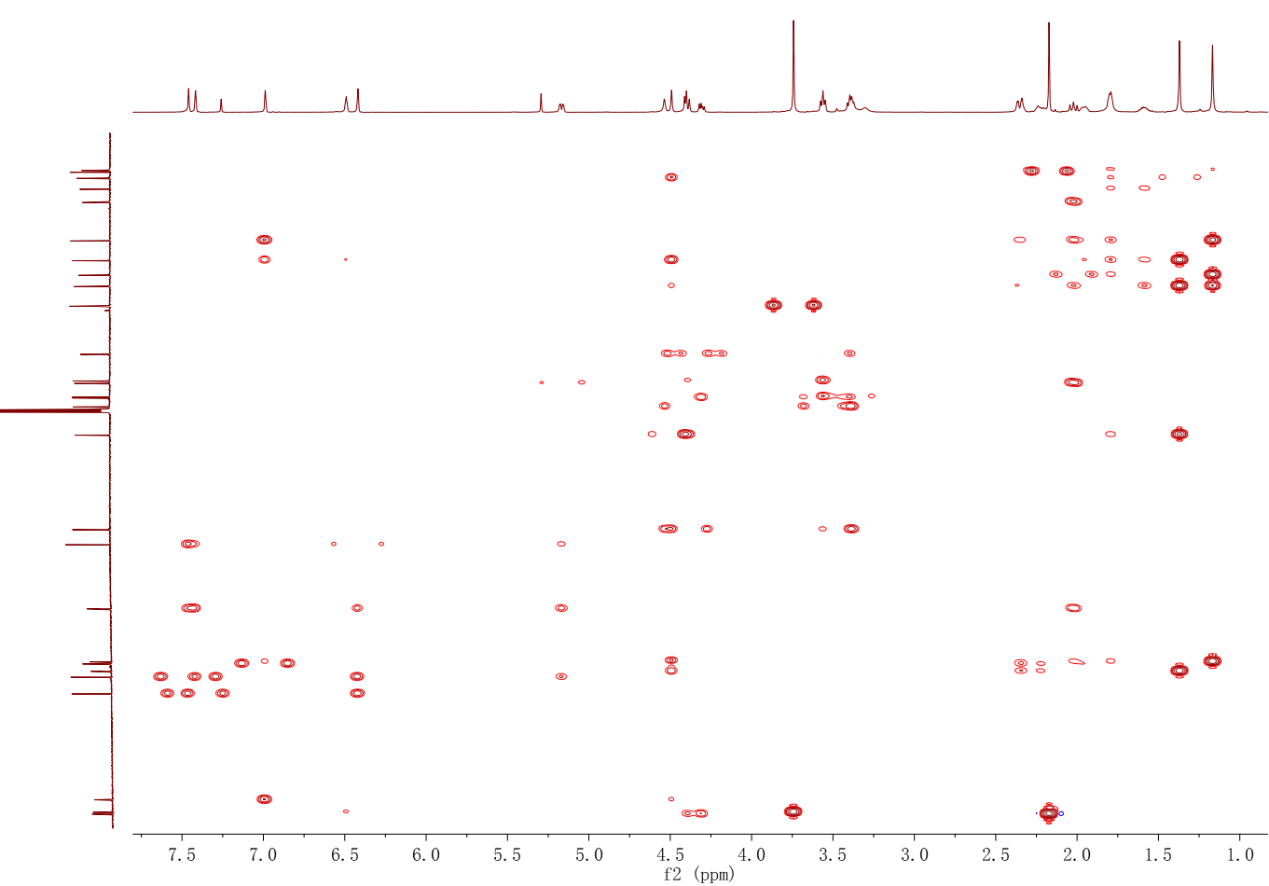


Figure S20. The NOESY spectrum of 3 in CDCl_3_.


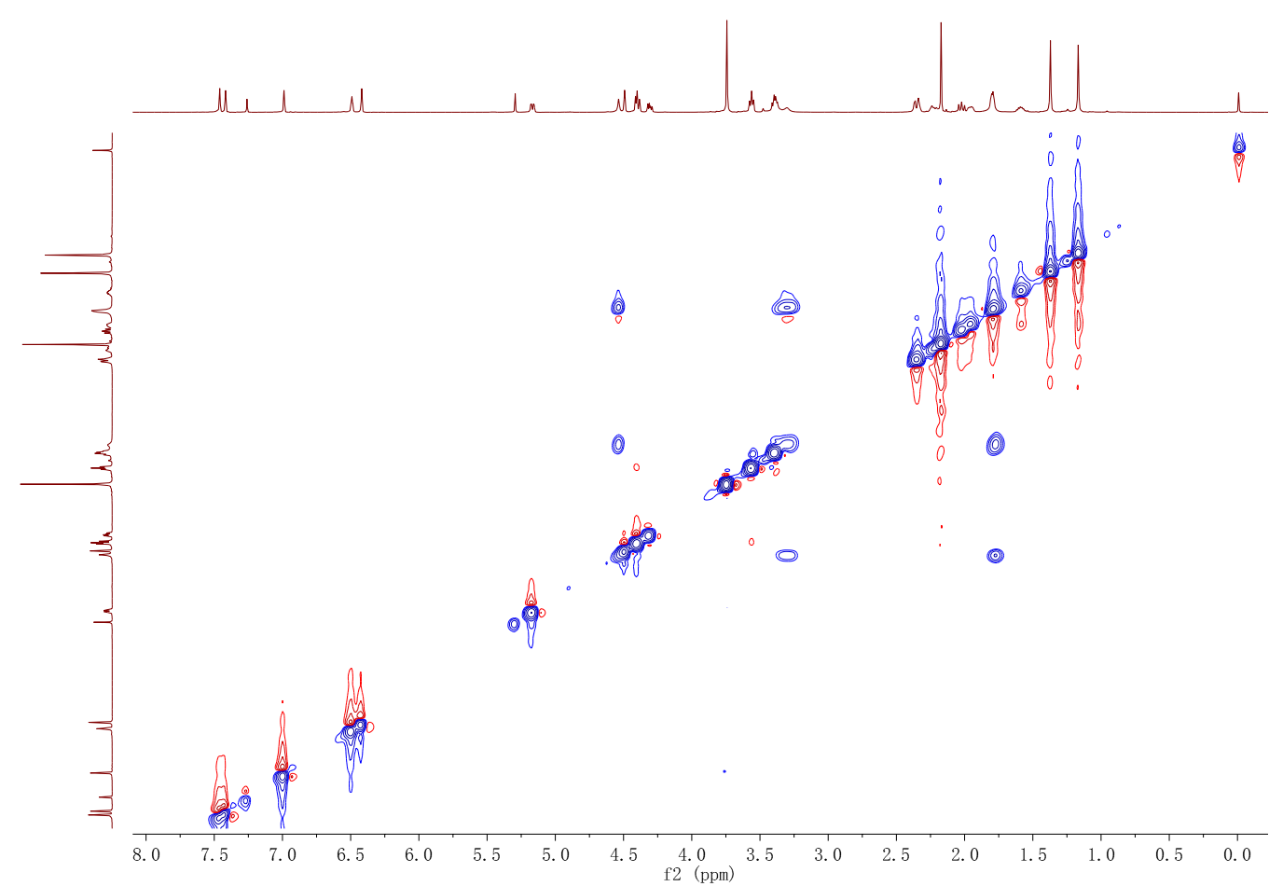


Figure S21. The (+)-HR-ESIMS spectrum of 3.

Figure S22. The ^1^H NMR spectrum of 4 in CDCl_3_.


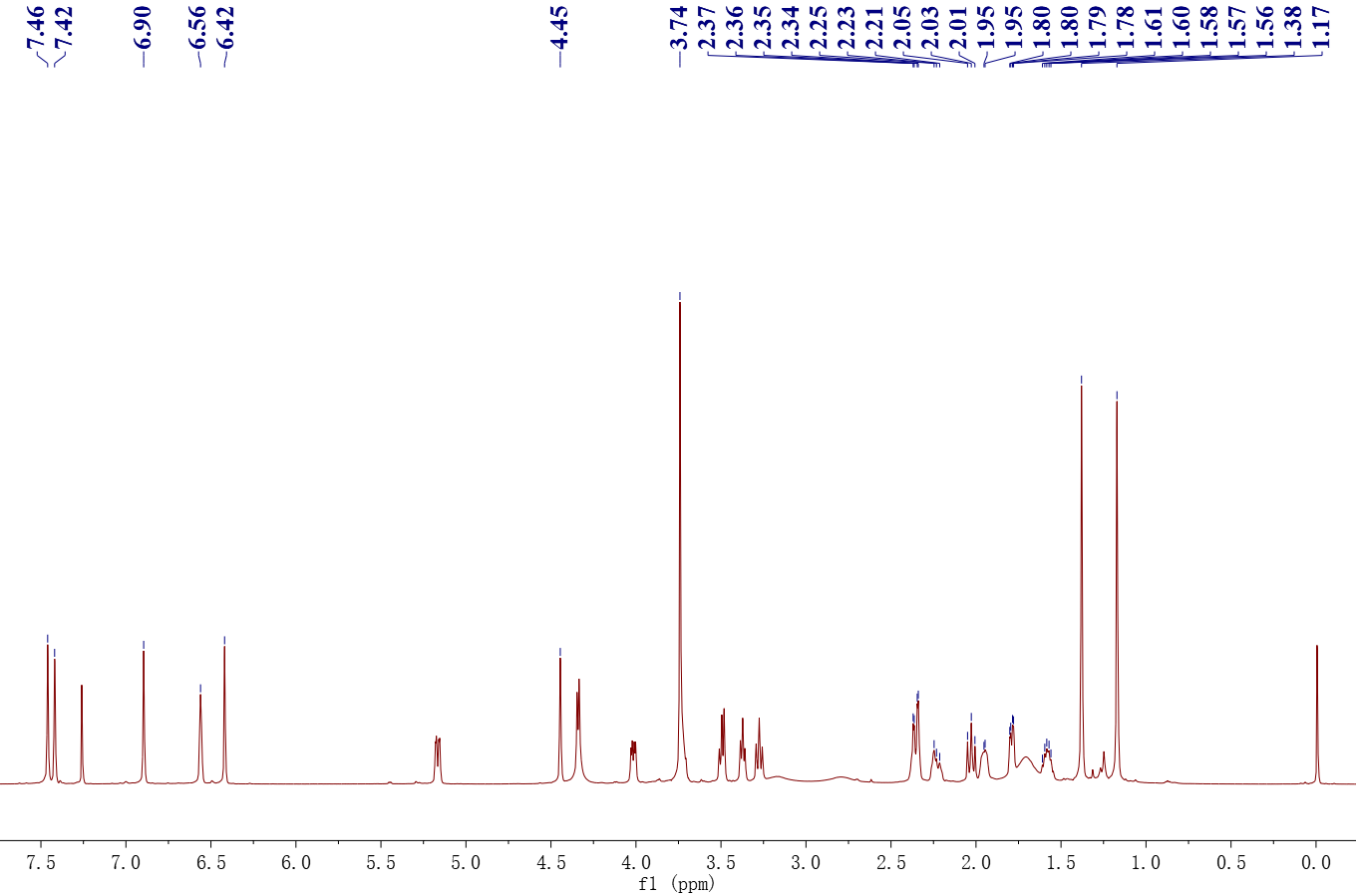


Figure S23. The ^13^C NMR spectrum of 4 in CDCl_3_.


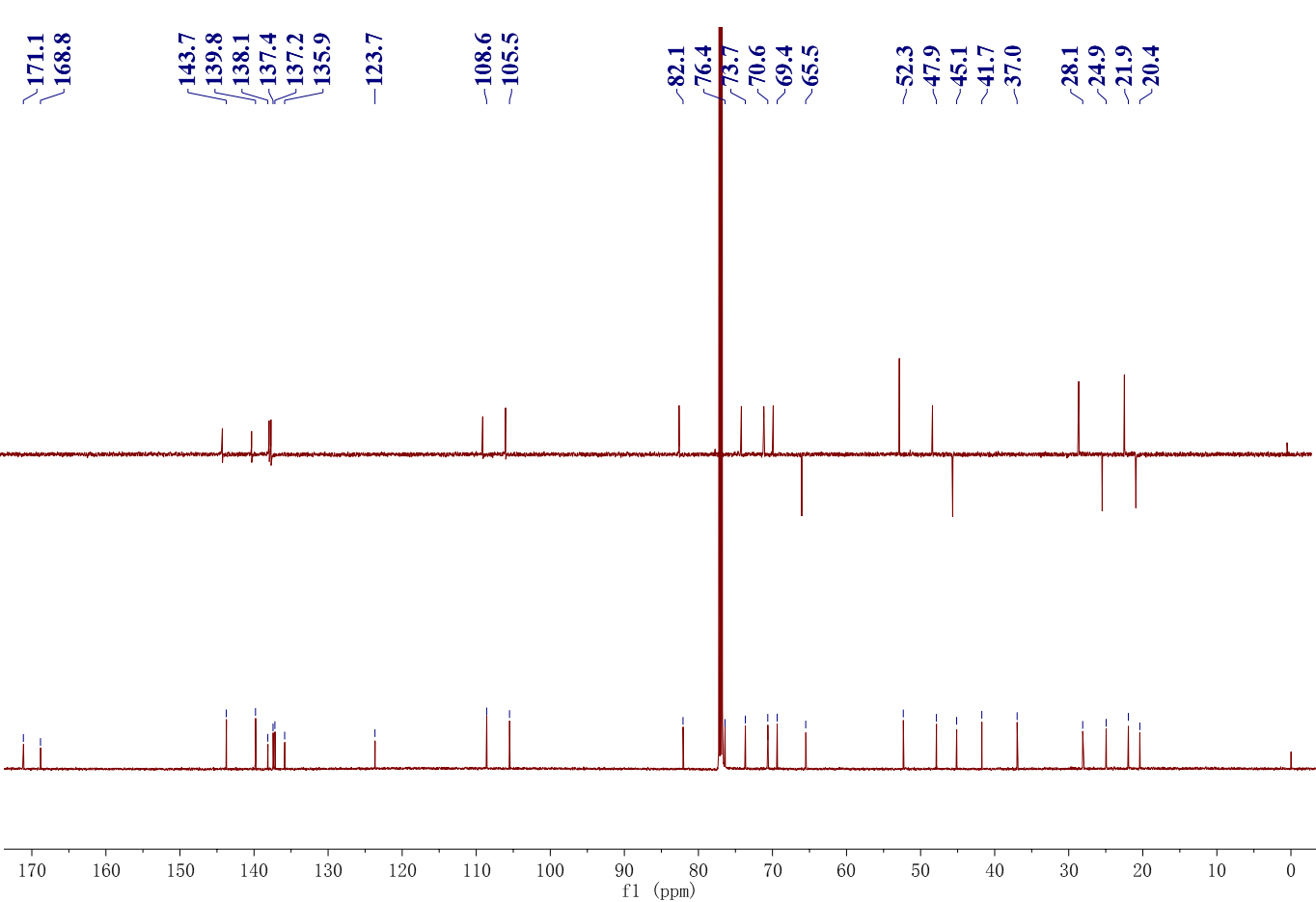


Figure S24. The ^1^H-^1^H COSY spectrum of 4 in CDCl_3_.


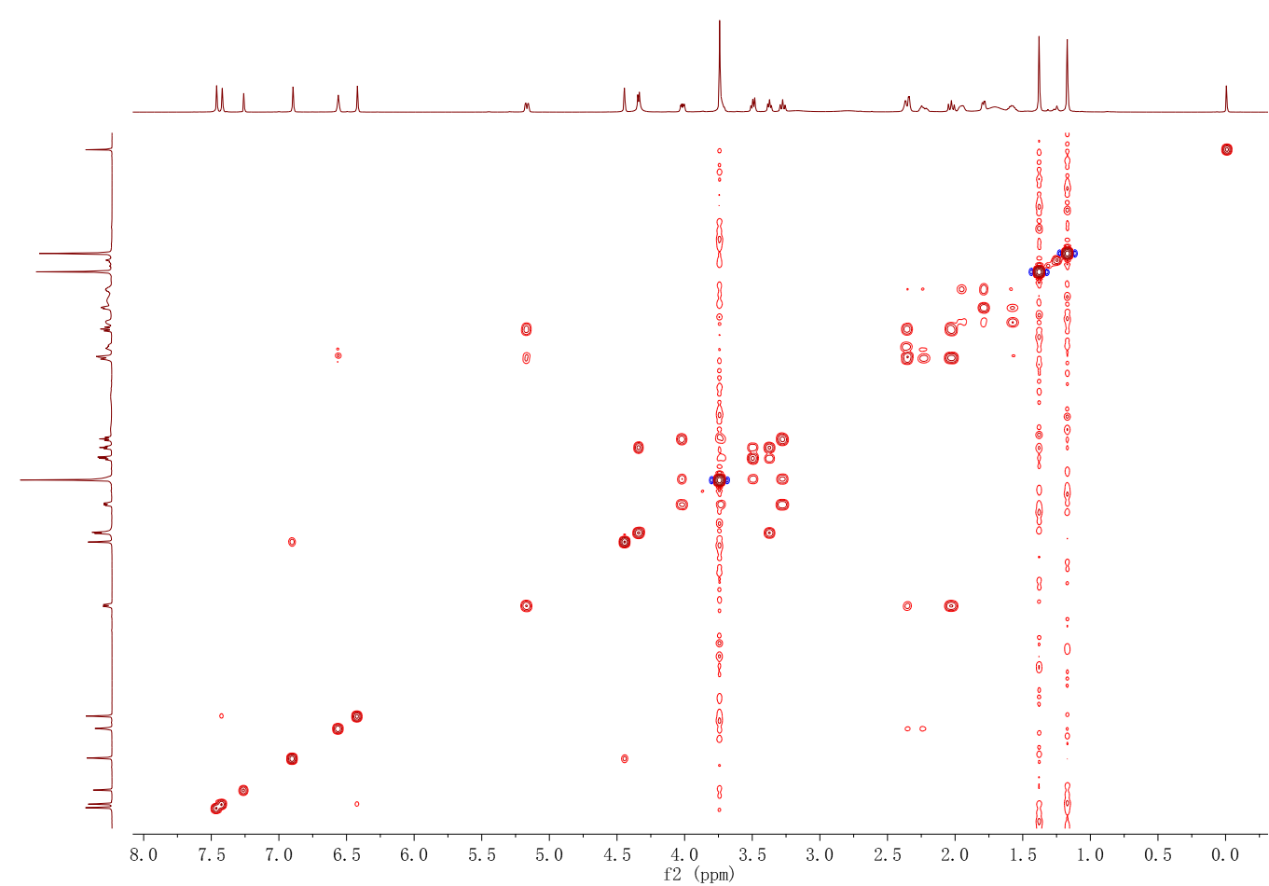


Figure S25. The HSQC spectrum of 4 in CDCl_3_.


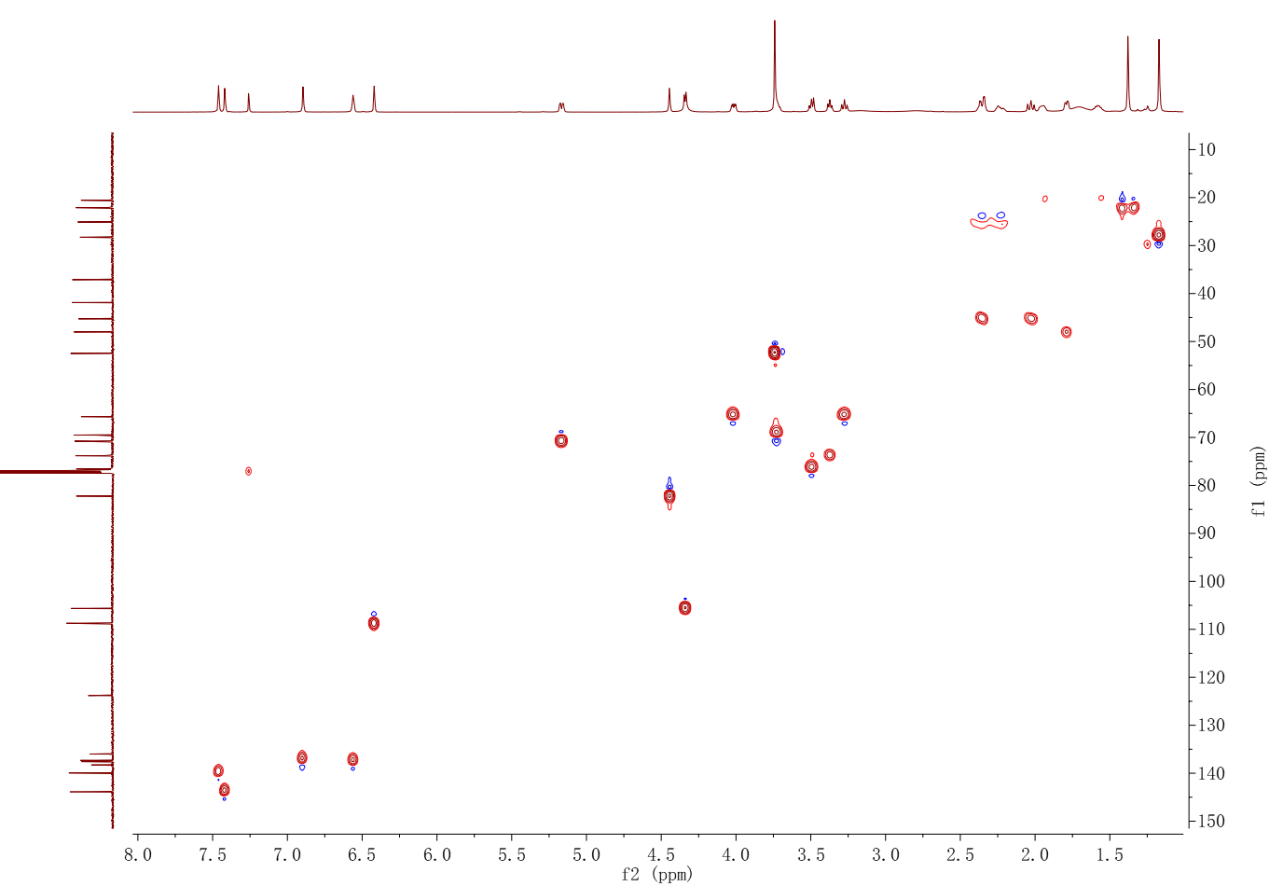


Figure S26. The HMBC spectrum of 4 in CDCl_3_.


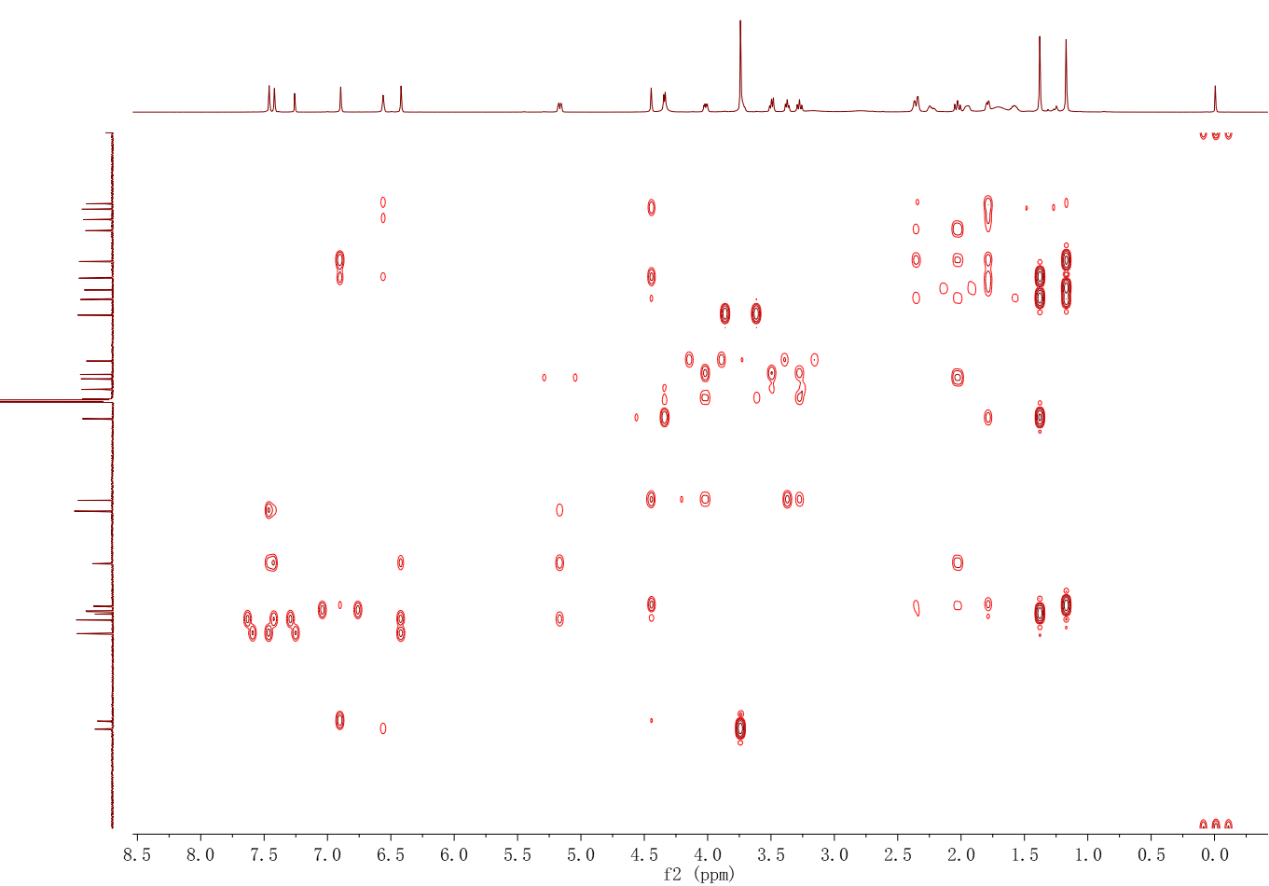


Figure S27. The NOESY spectrum of 4 in CDCl_3_.


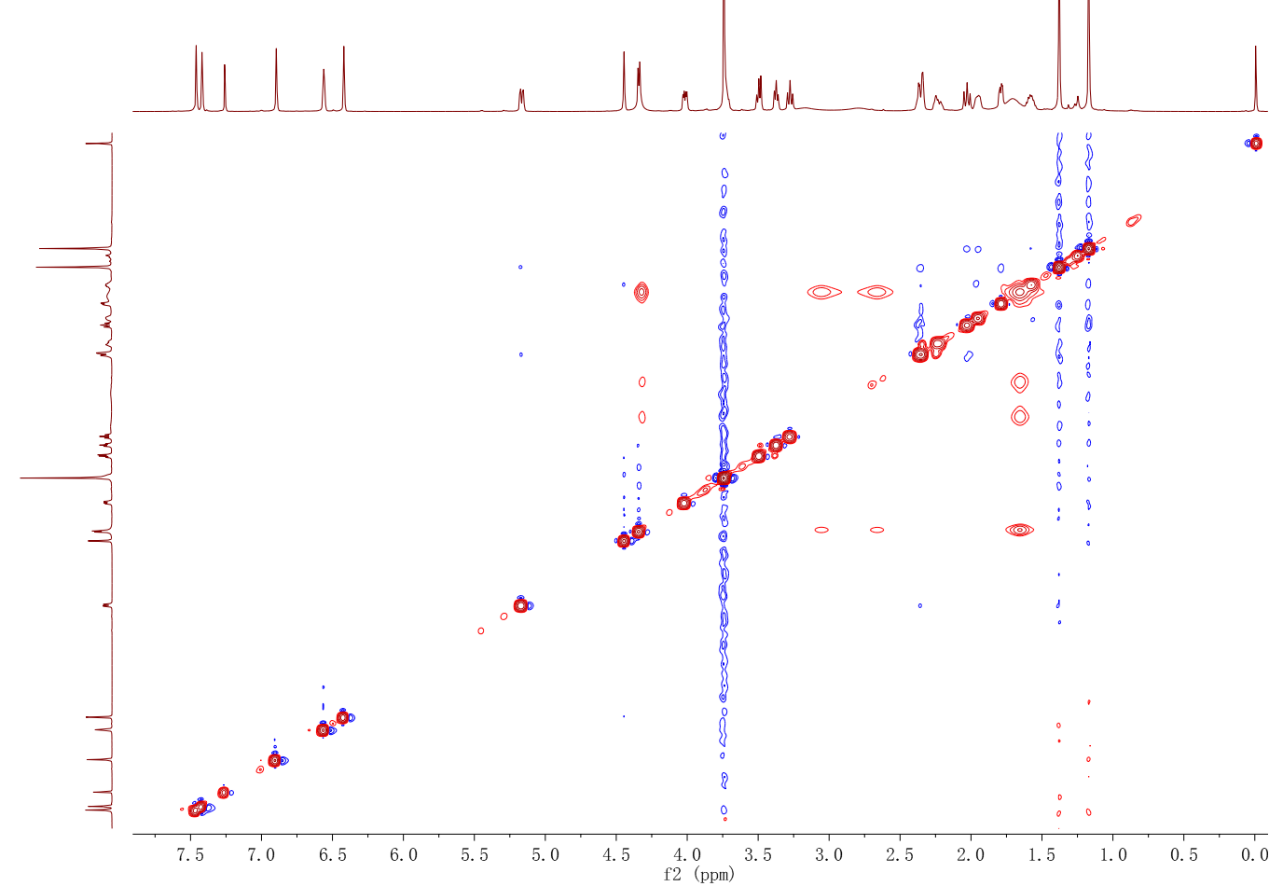


Figure S28. The (–)-HR-ESIMS spectrum of 4.

Figure S29. The ^1^H NMR spectrum of 5 in CD_3_OD.


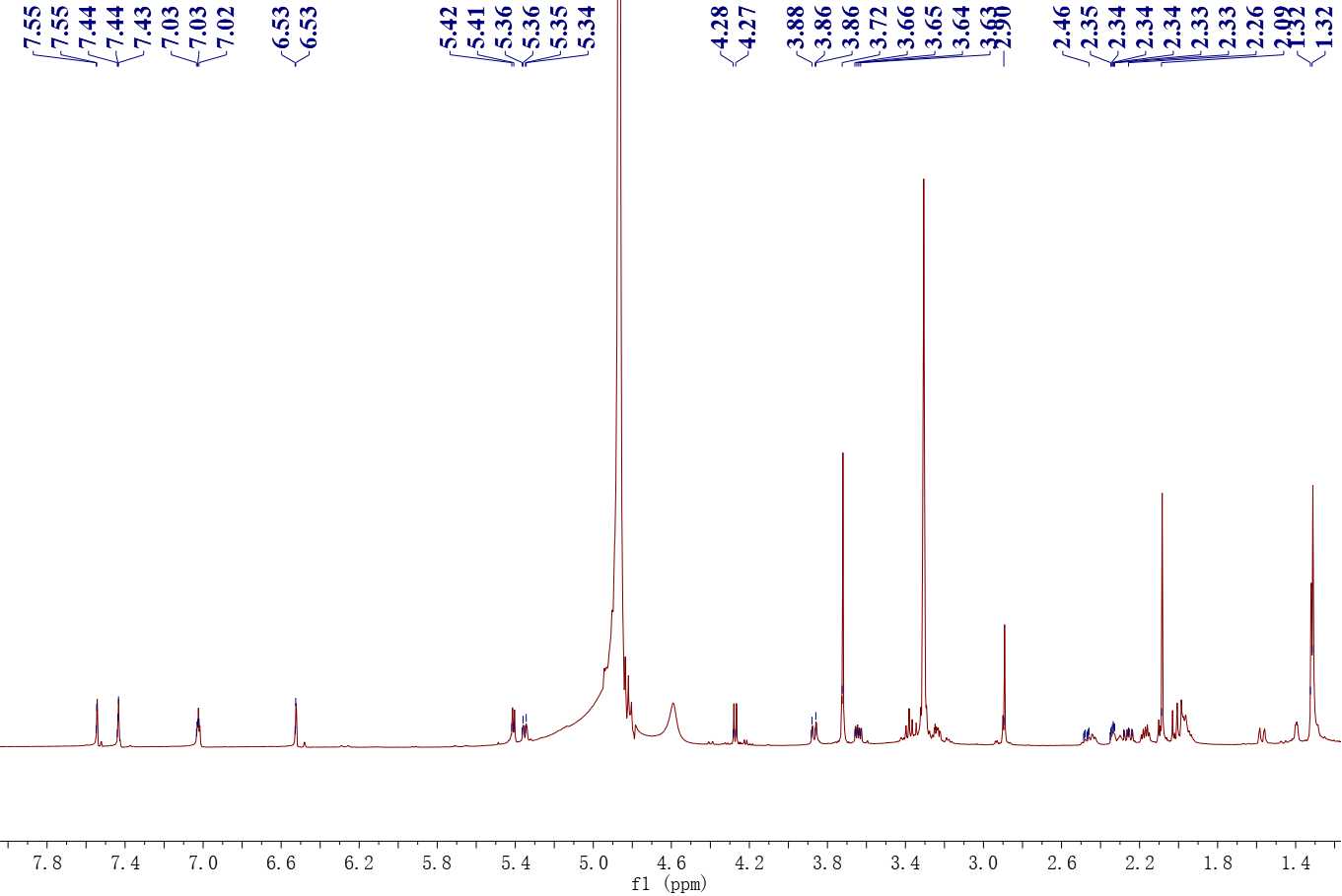


Figure S30. The ^13^C NMR spectrum of 5 in CD_3_OD.


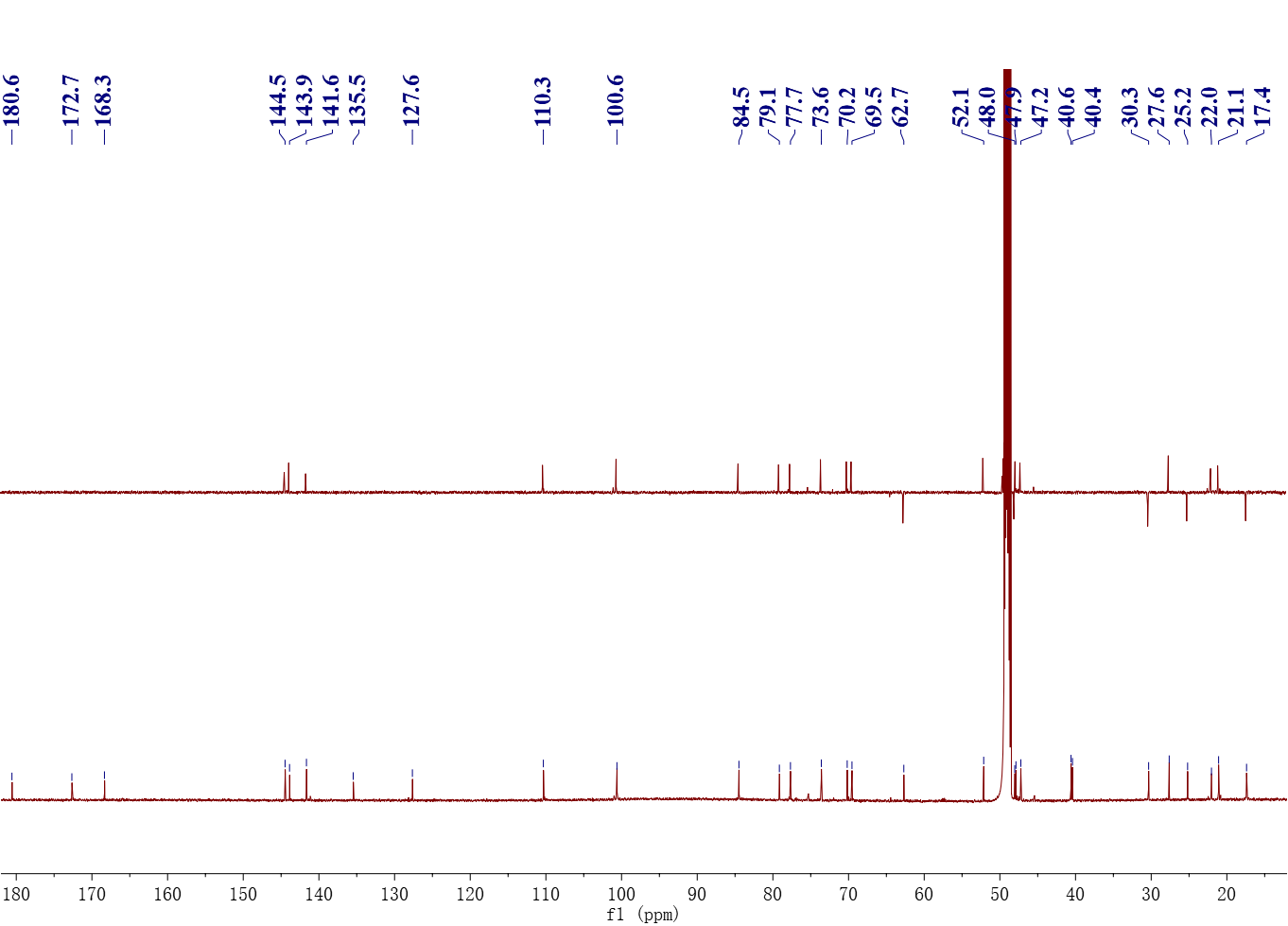


Figure S31. The ^1^H-^1^H COSY spectrum of 5 in CD_3_OD.


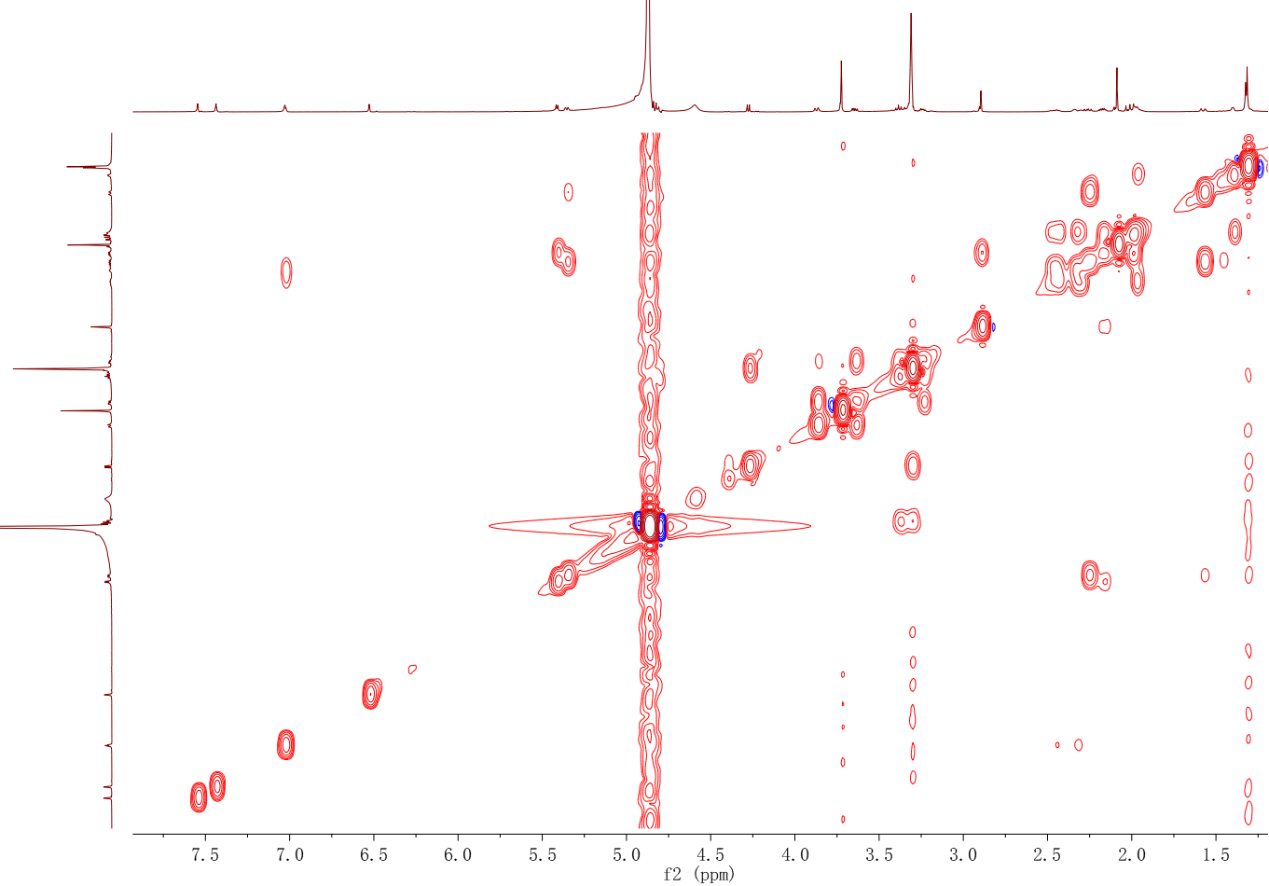


Figure S32. The HSQC spectrum of 5 in CD_3_OD.


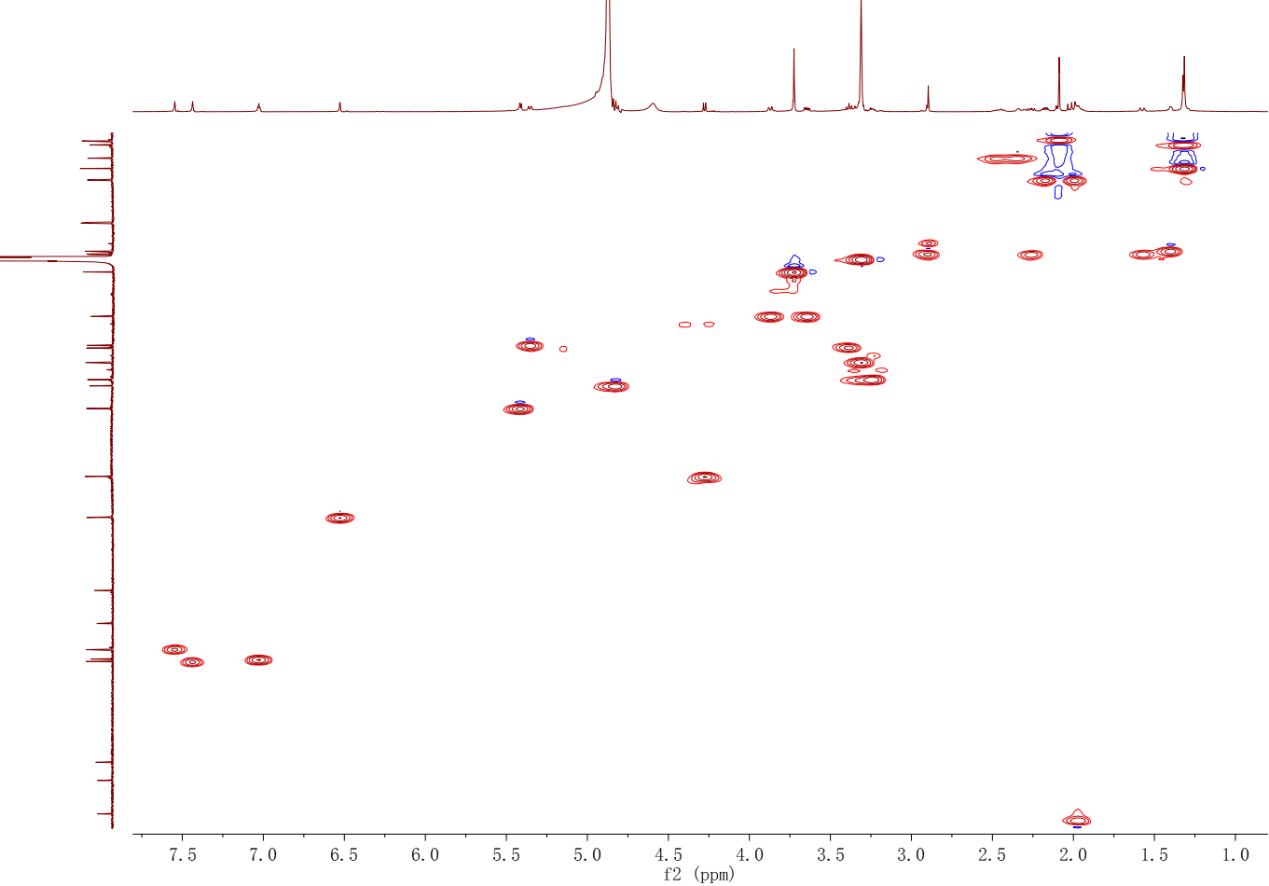


Figure S33. The HMBC spectrum 5 in CD_3_OD.


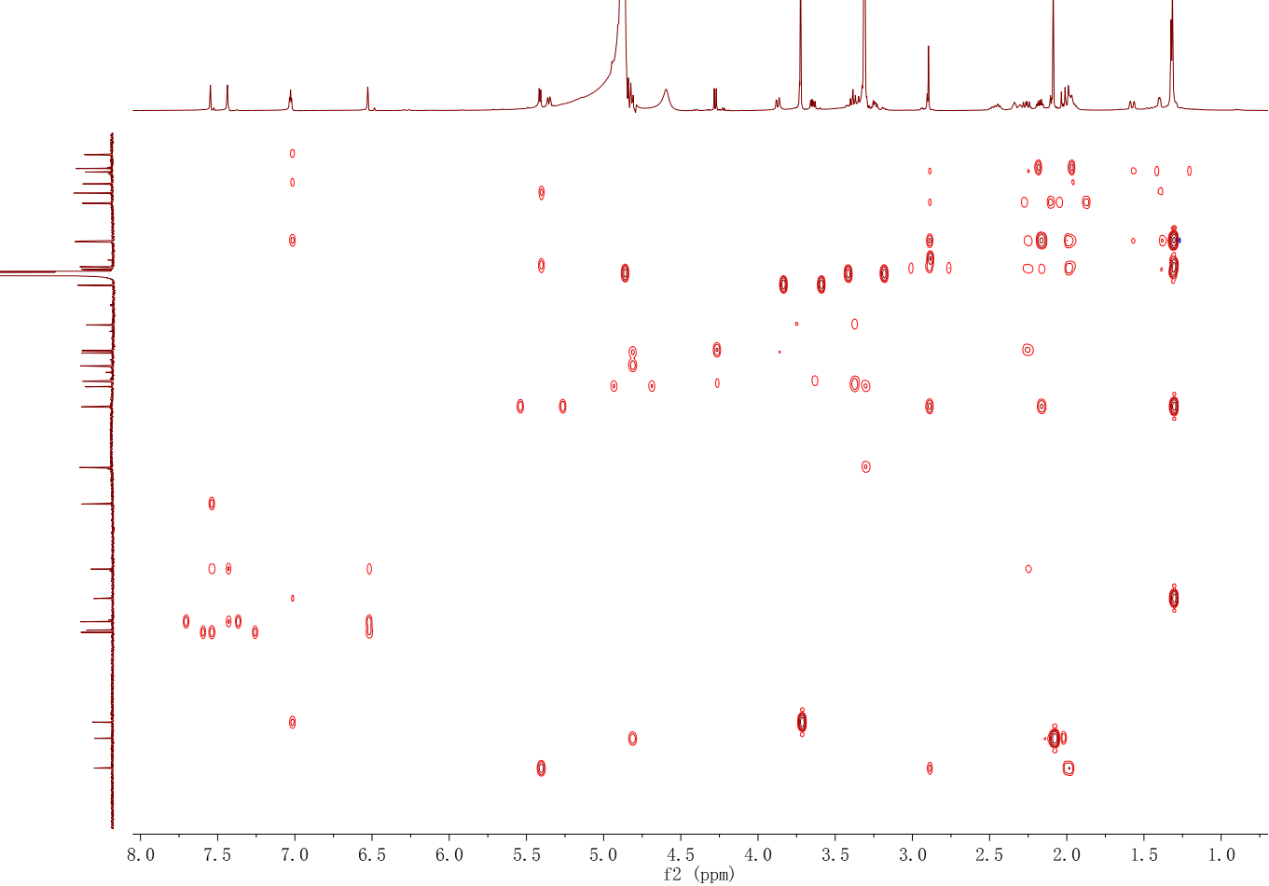


Figure S34. The NOESY spectrum of 5 in CD_3_OD.


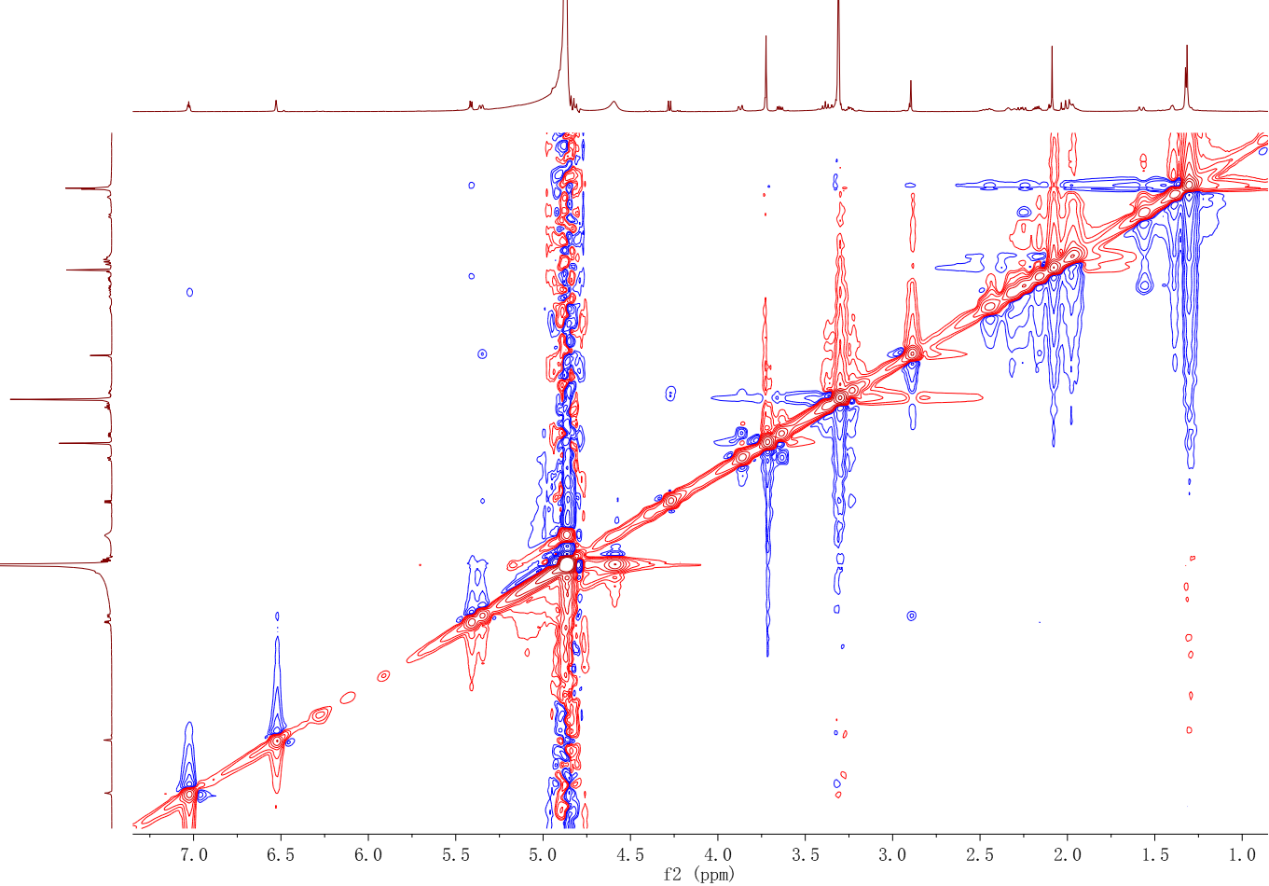


Figure S35. The (+)-HR-ESIMS spectrum of 5.

Figure S36. The ^1^H NMR spectrum of 6 in CDCl_3_.


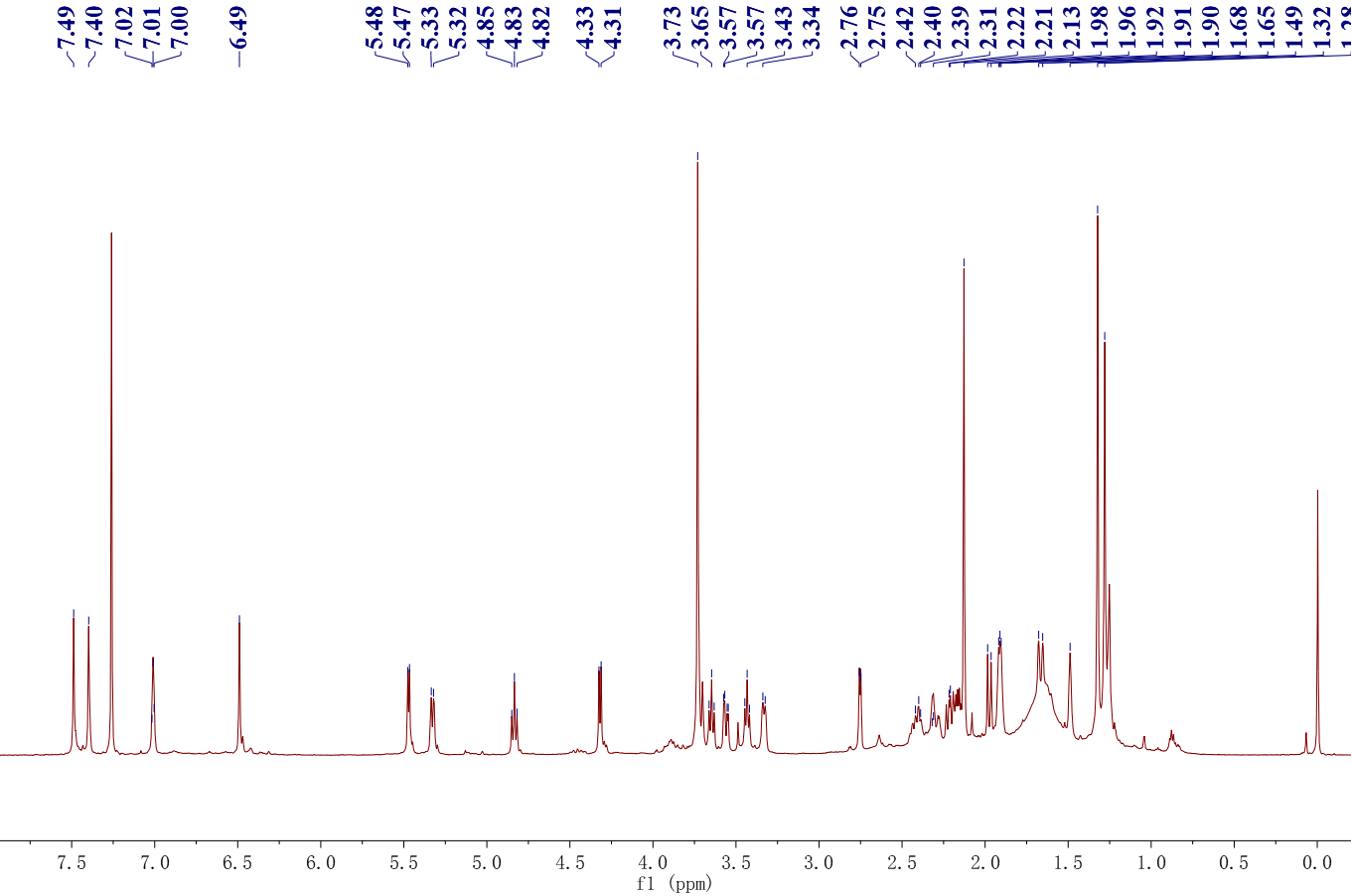


Figure S37. The ^13^C NMR spectrum of 6 in CDCl_3_.


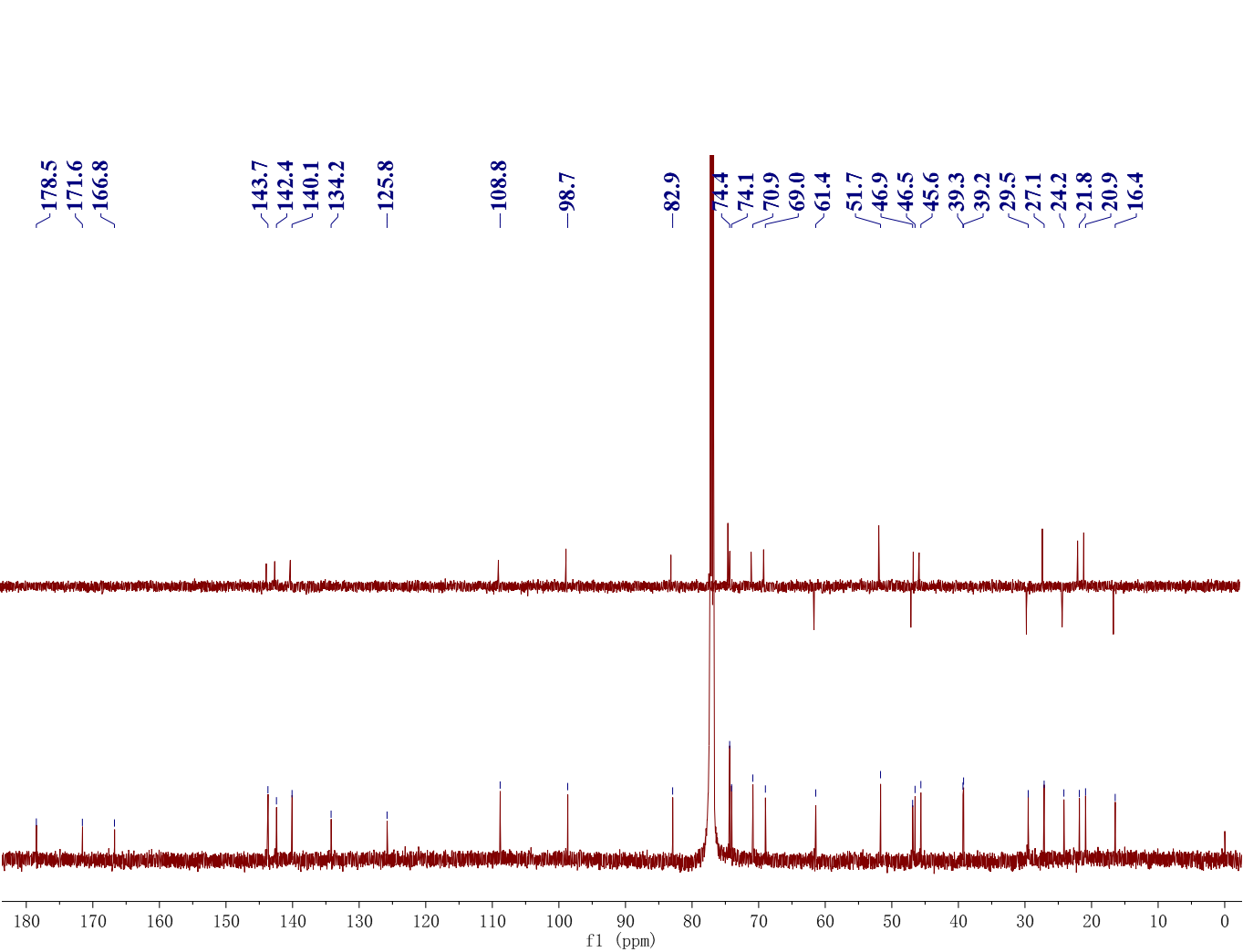


Figure S38. The ^1^H-^1^H COSY spectrum of 6 in CDCl_3_.


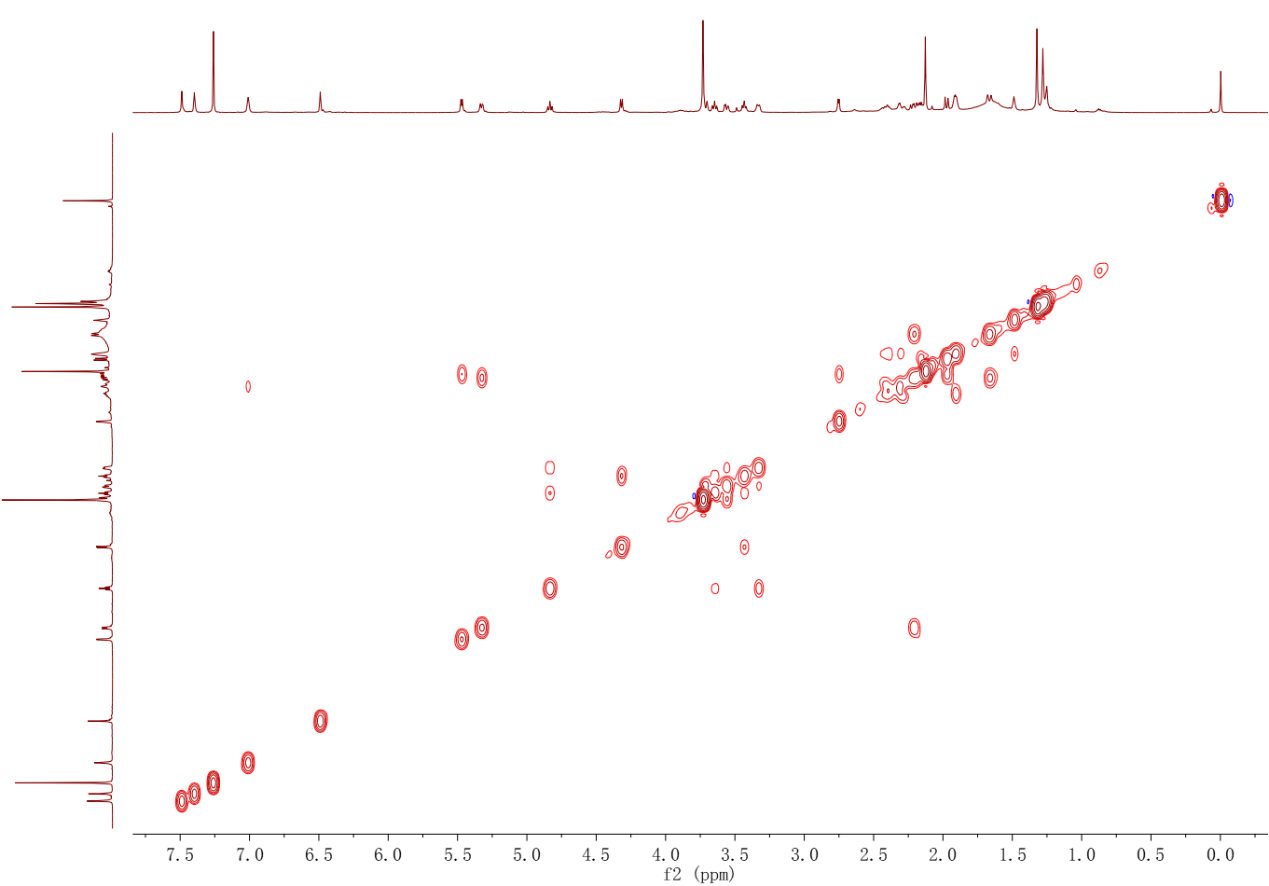


Figure S39. The HSQC spectrum of 6 in CDCl_3_.


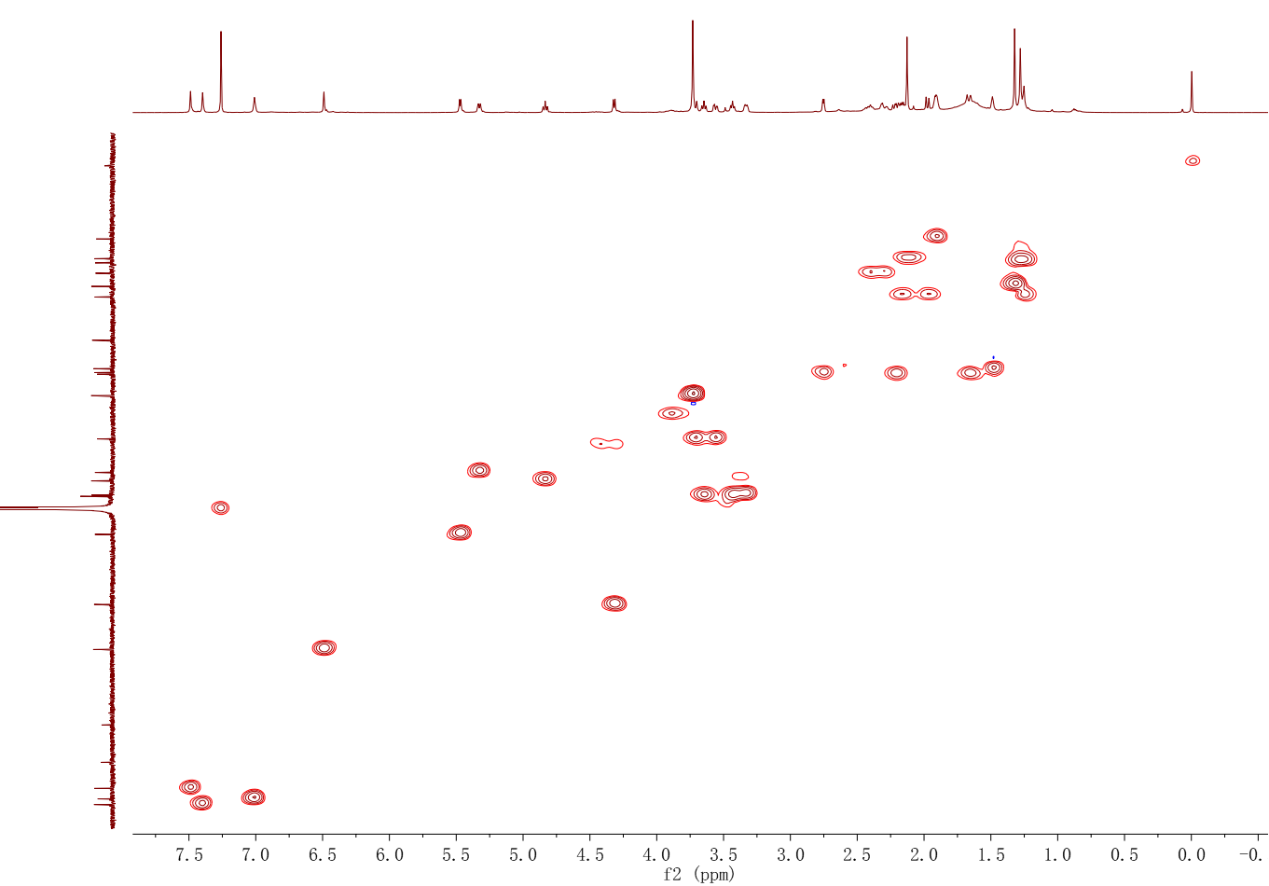


Figure S40. The HMBC spectrum 6 in CDCl_3_.


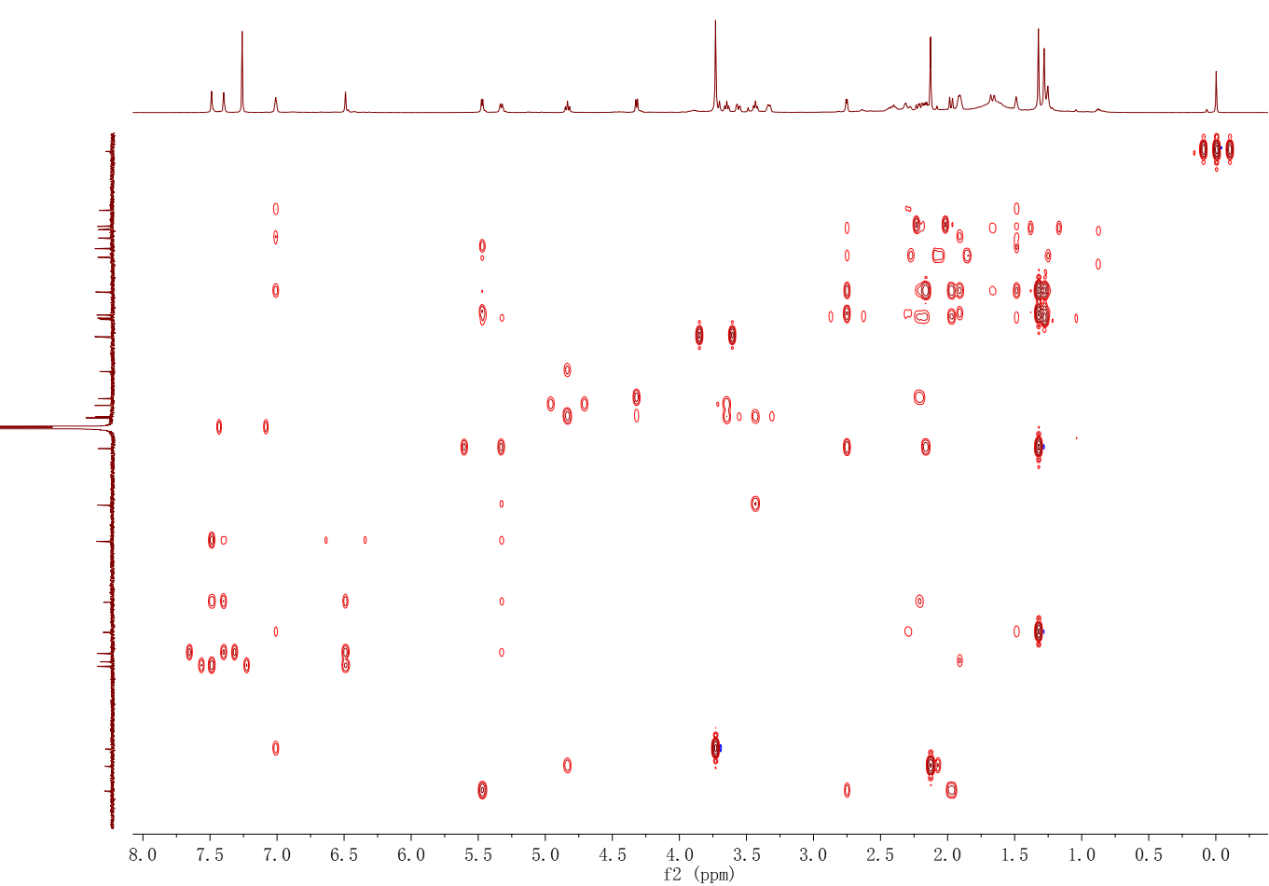


Figure S41. The NOESY spectrum of 6 in CDCl_3_.


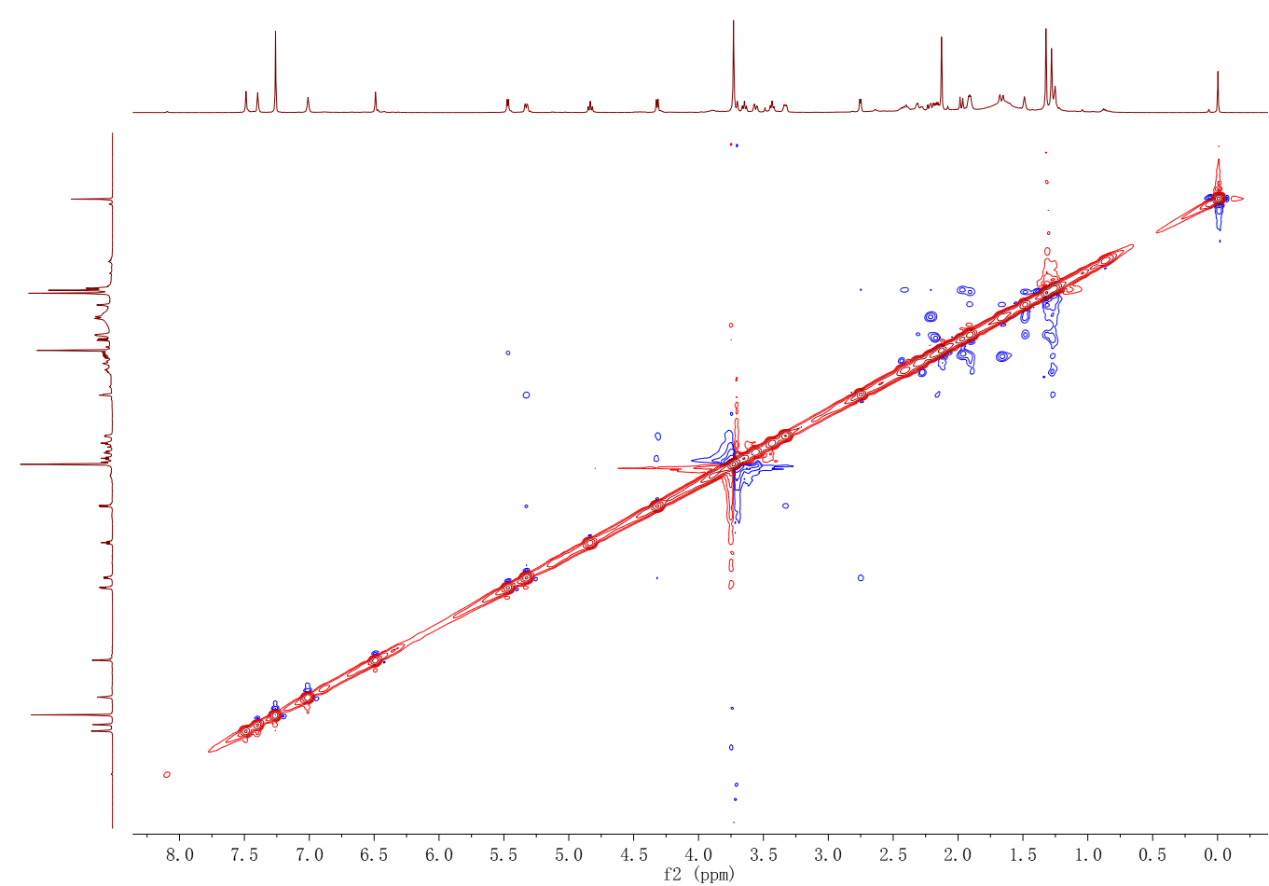


Figure S42. The (+)-HR-ESIMS spectrum of 6.

Figure S43. The ^1^H NMR spectrum of 7 in CDCl_3_.


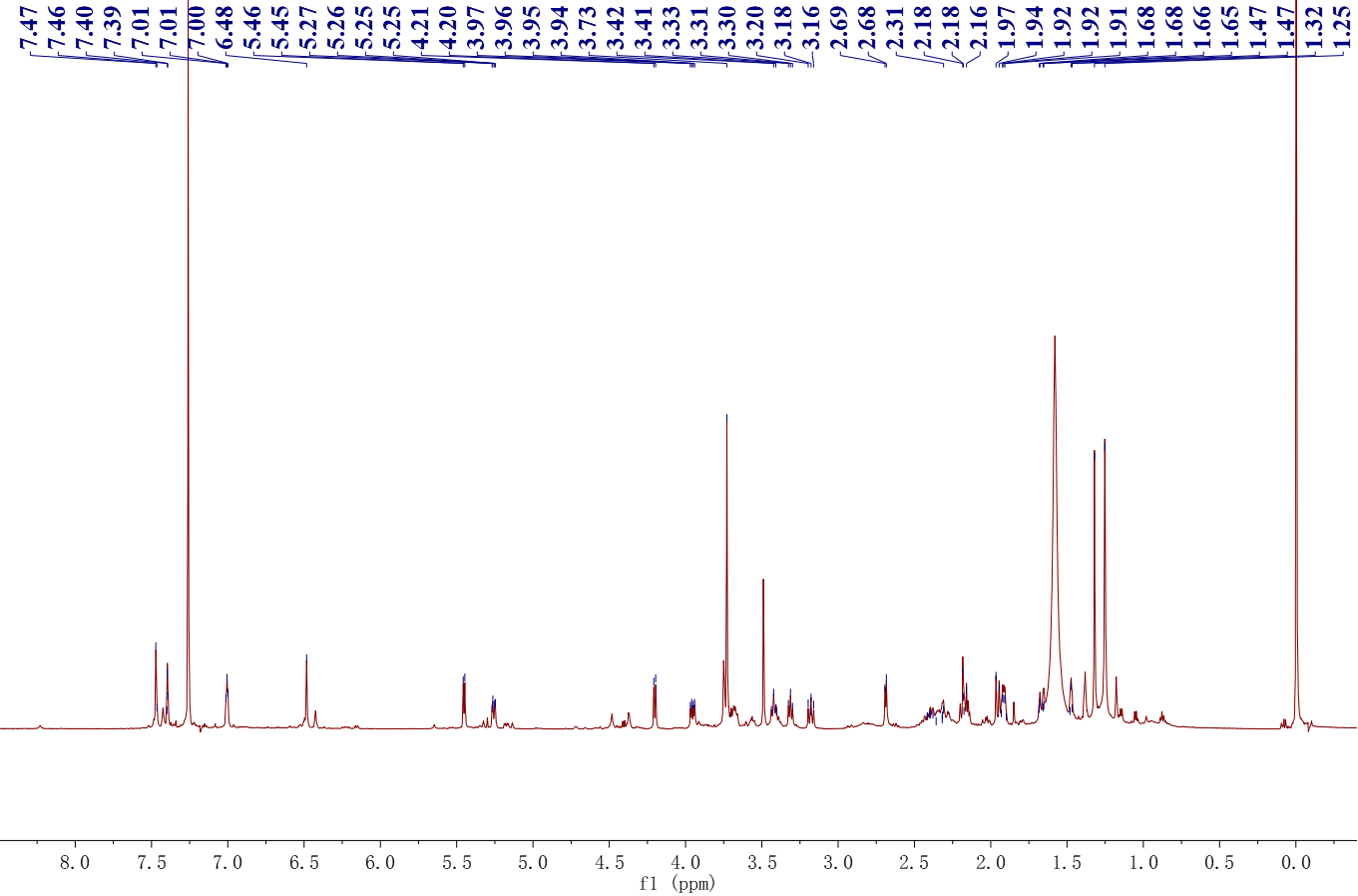


Figure S44. The ^13^C NMR spectrum of 7 in CDCl_3_.


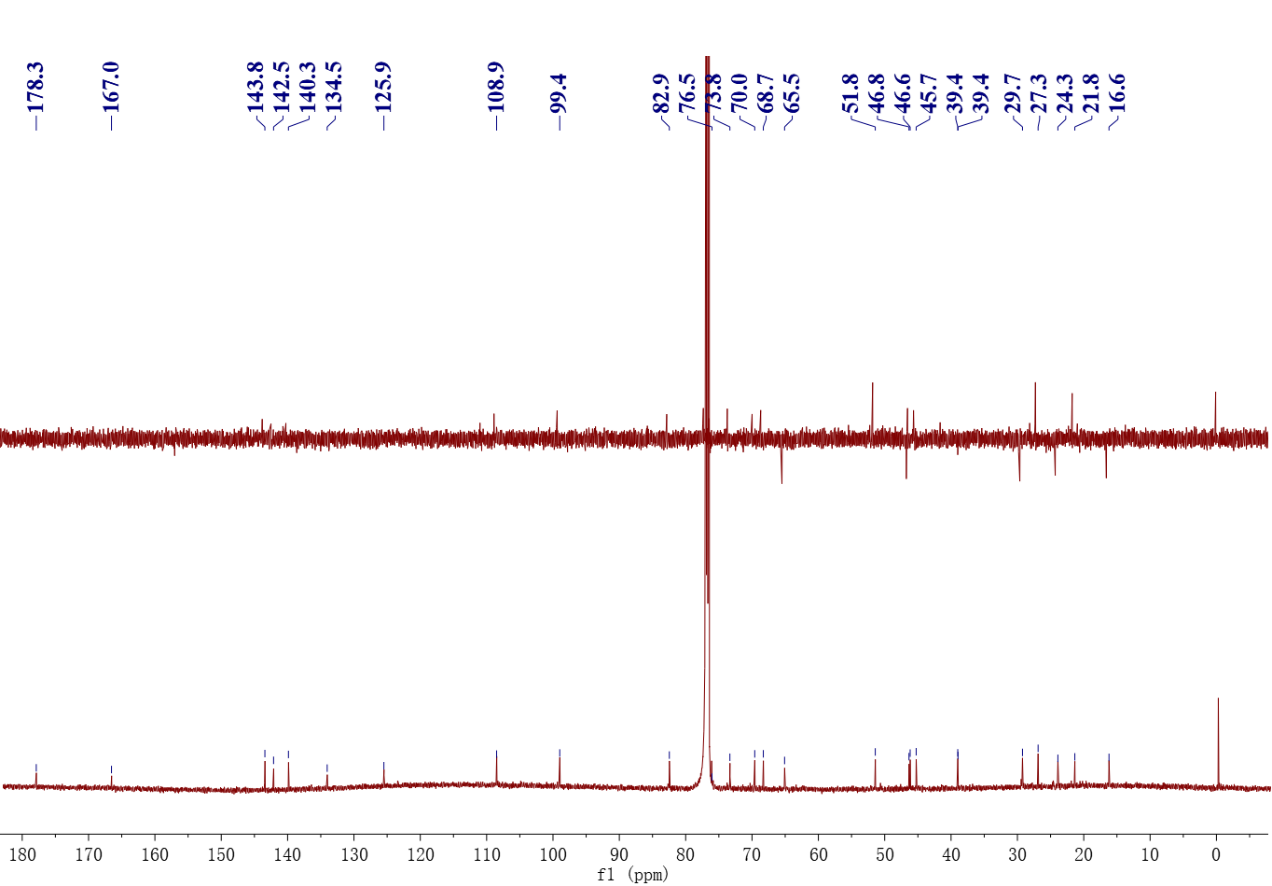


Figure S45. The ^1^H-^1^H COSY spectrum of 7 in CDCl_3_.


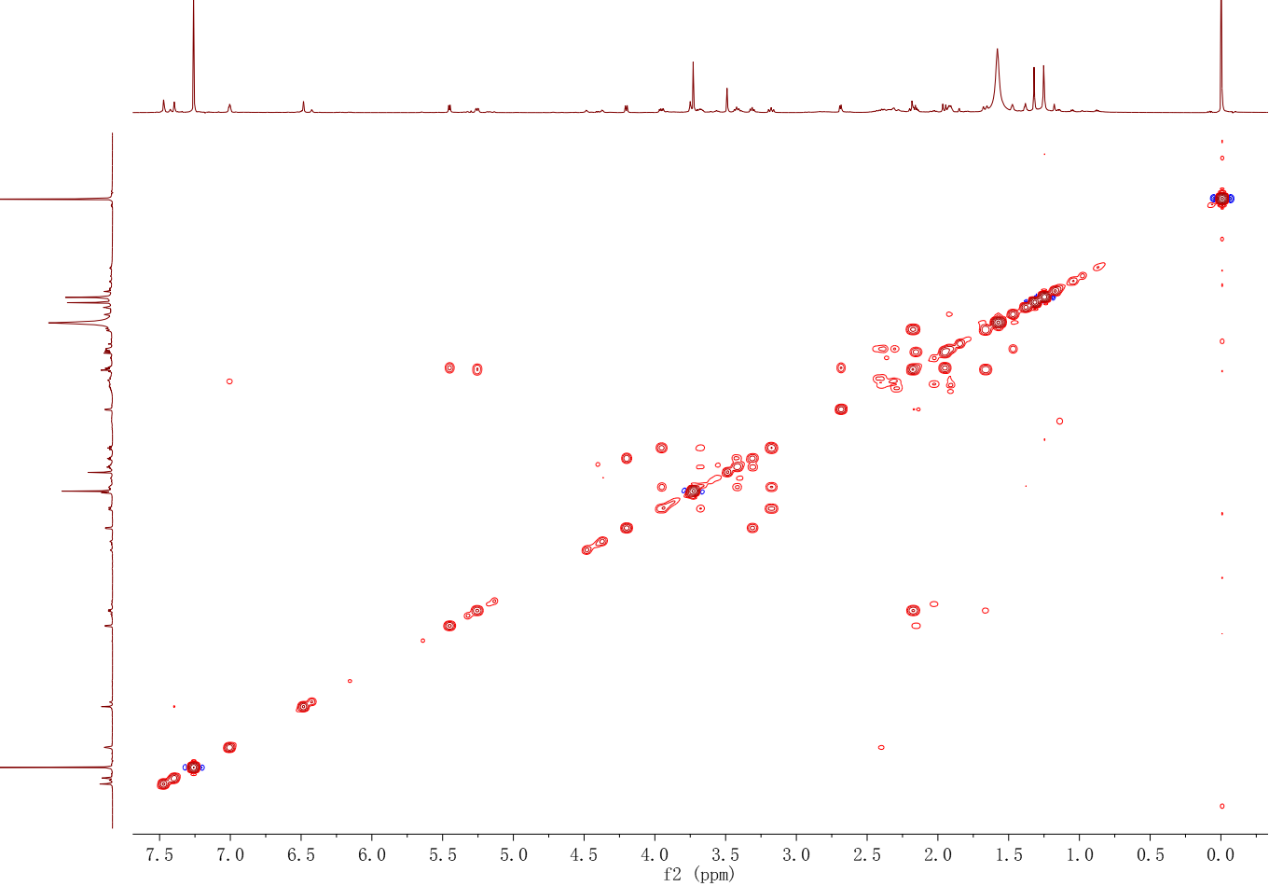


Figure S46. The HSQC spectrum of 7 in CDCl_3_.


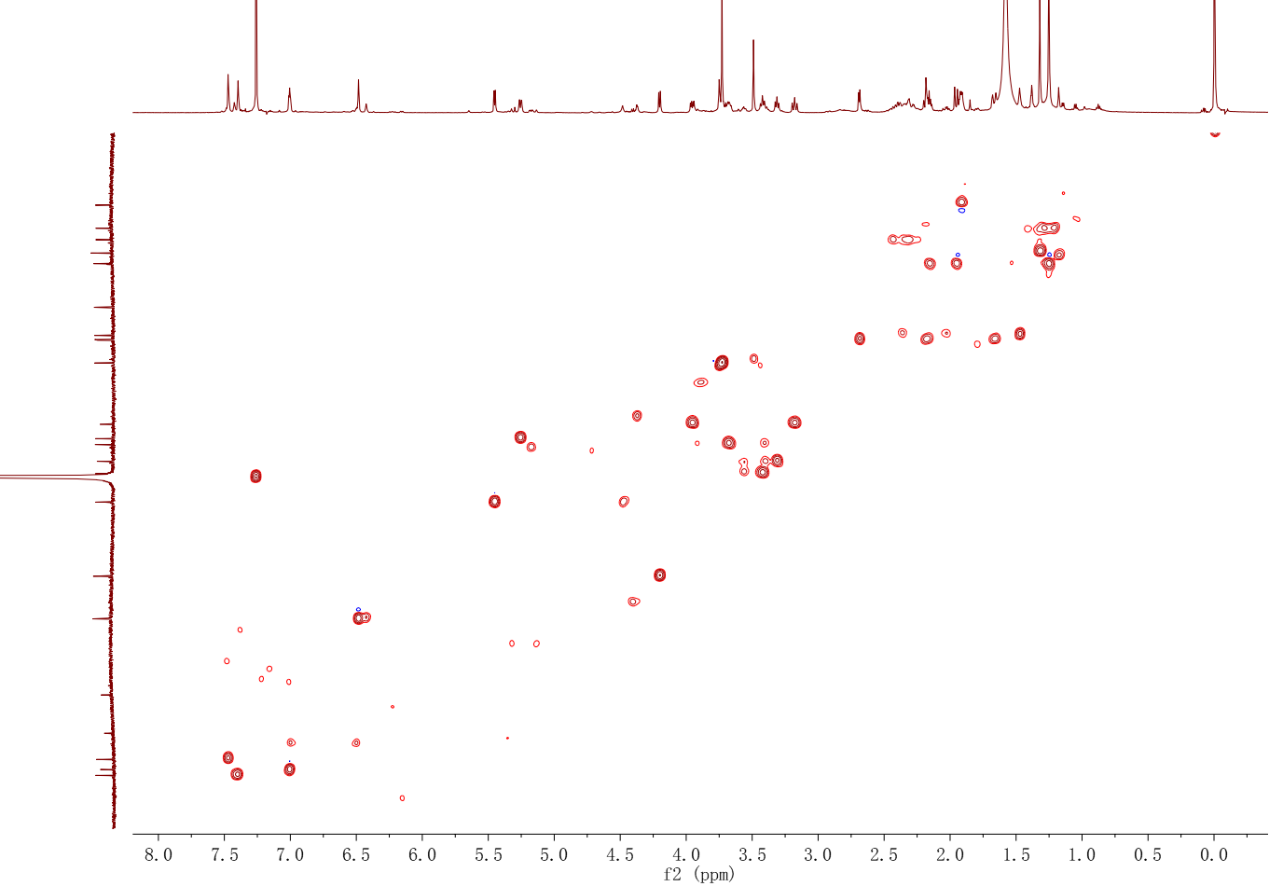


Figure S47. The HMBC spectrum 7 in CDCl_3_.


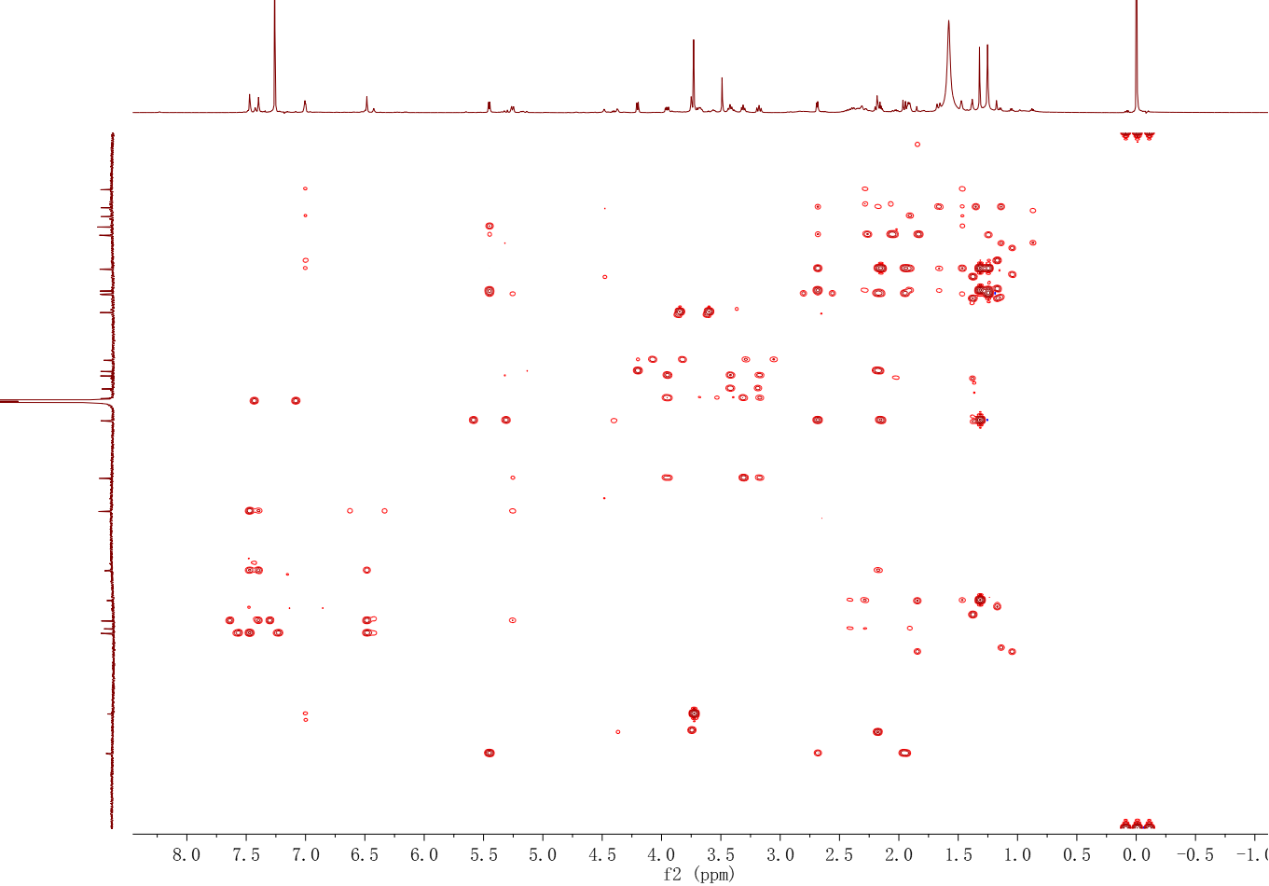


Figure S48. The NOESY spectrum of 7 in CDCl_3_.


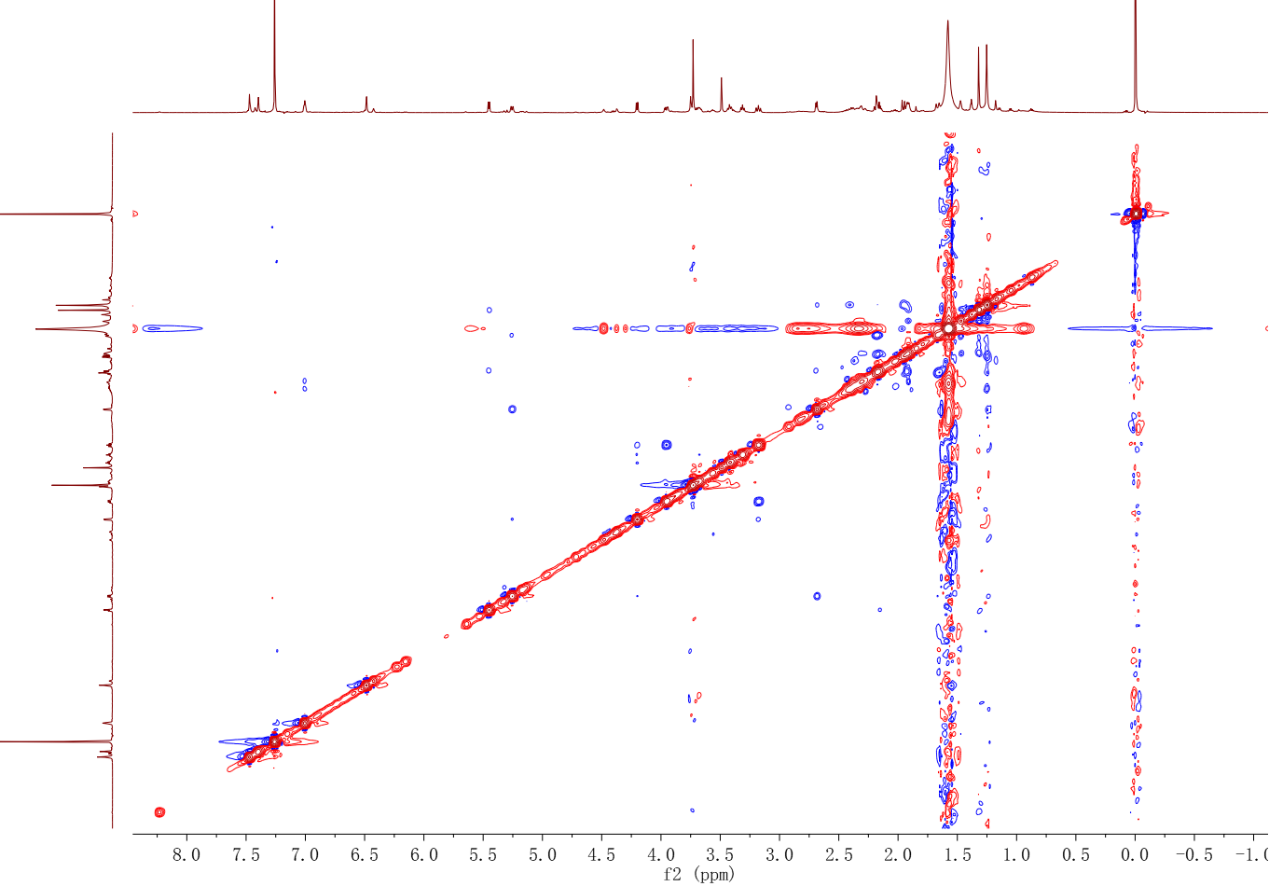


Figure S49. The (+)-HR-ESIMS spectrum of 7.

Figure S50. The ^1^H NMR spectrum of 8 in CD_3_OD.


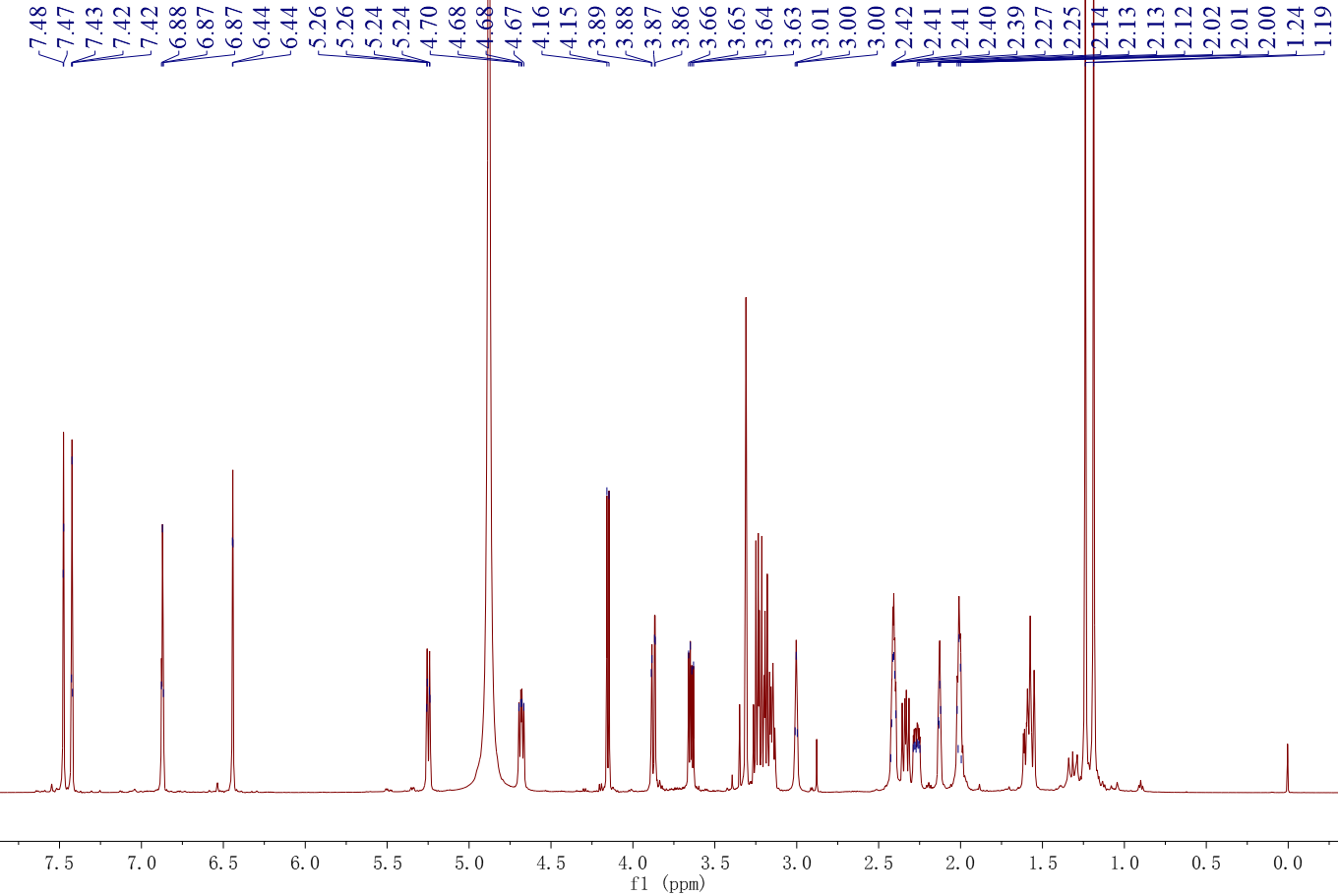


Figure S51. The ^13^C NMR spectrum of 8 in CD_3_OD.


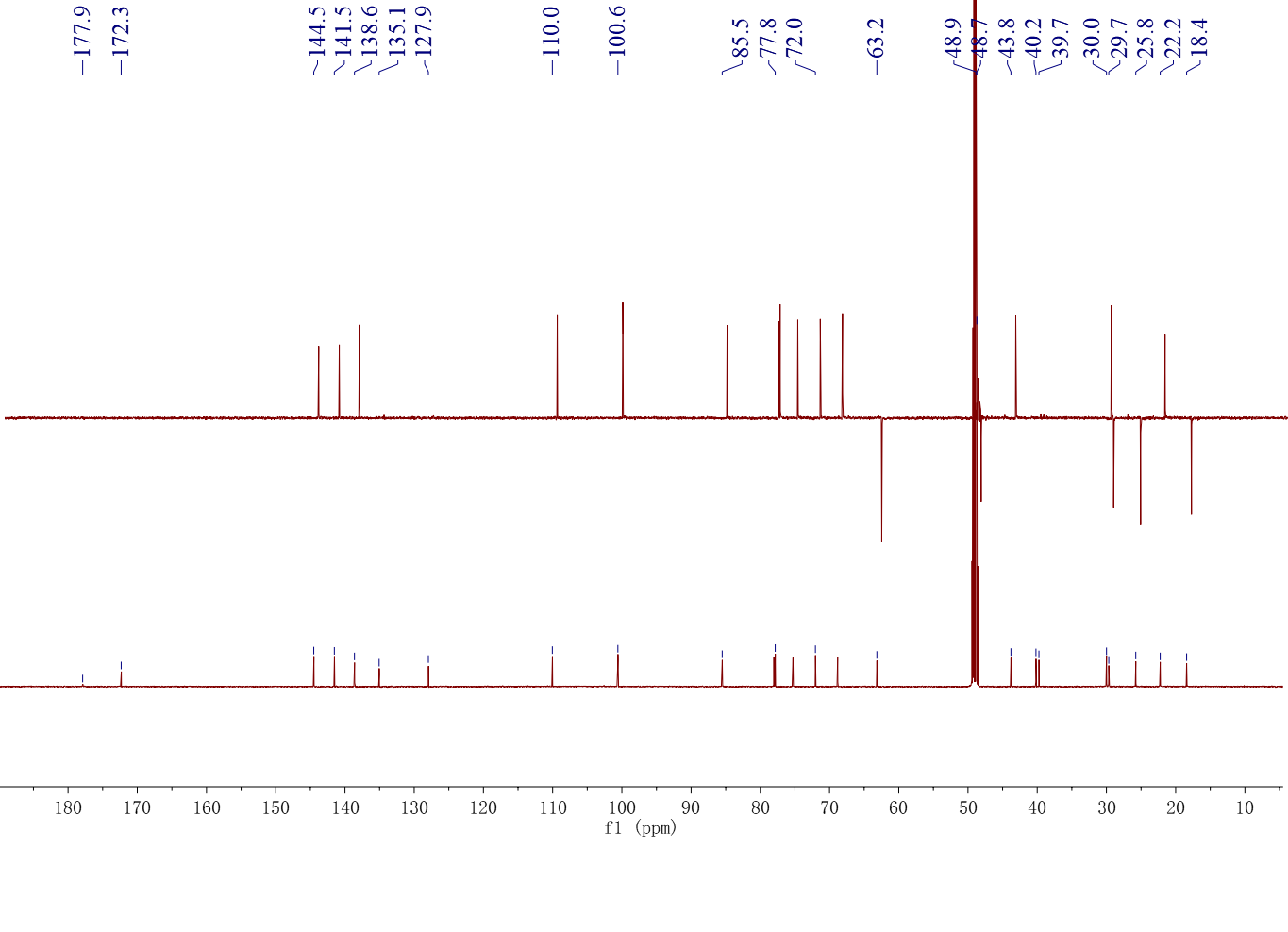


Figure S52. The ^1^H-^1^H COSY spectrum of 8 in CD_3_OD.


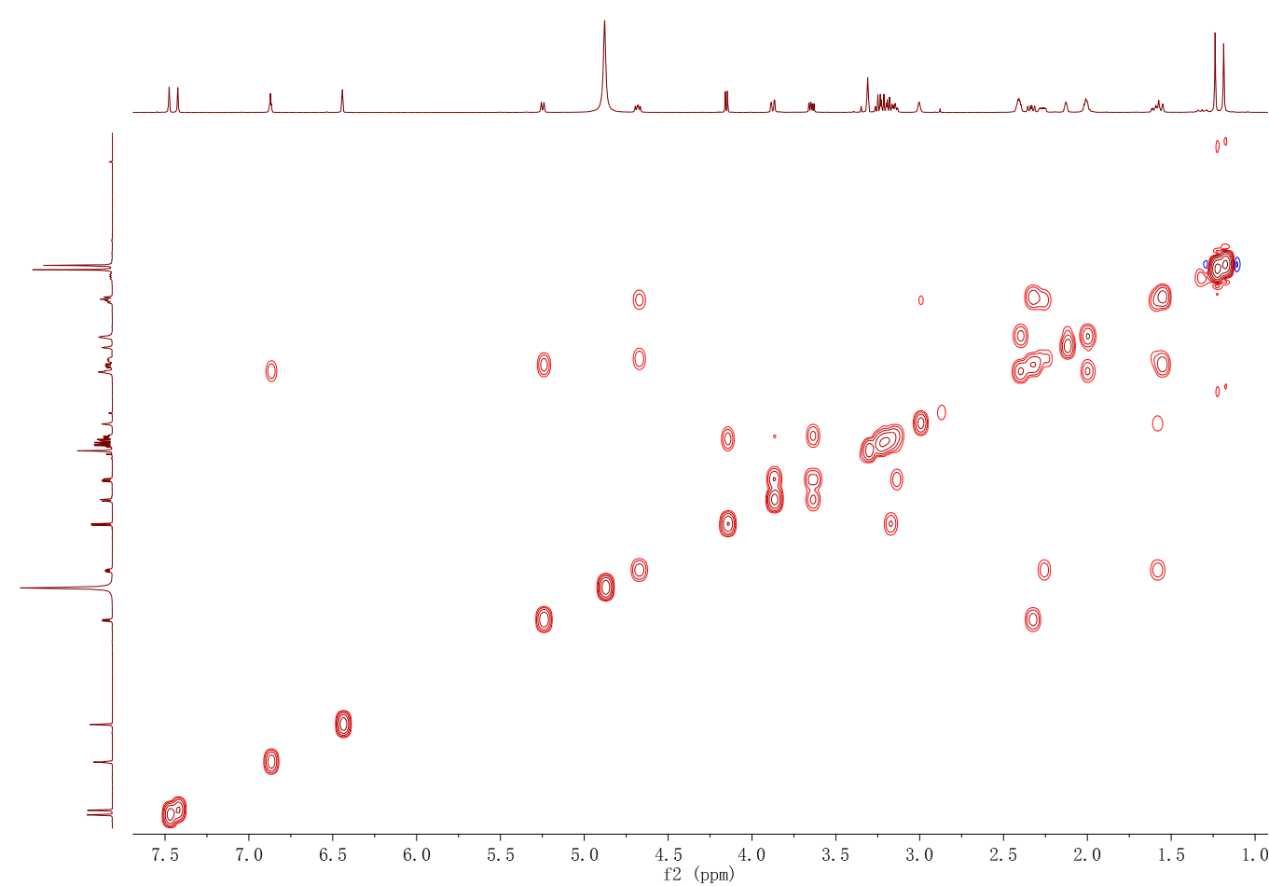


Figure S53. The HSQC spectrum of 8 in CD_3_OD.


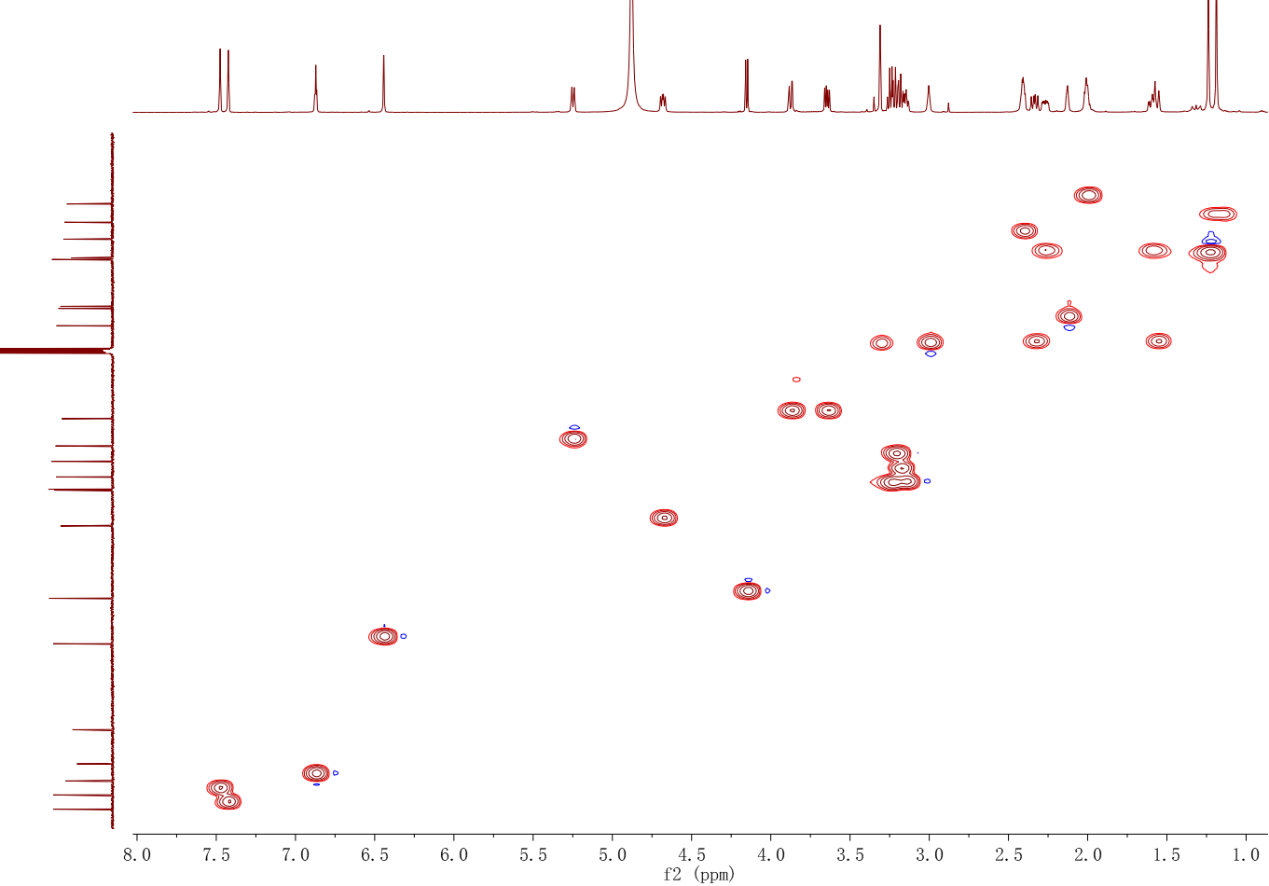


Figure S54. The HMBC spectrum 8 in CD_3_OD.


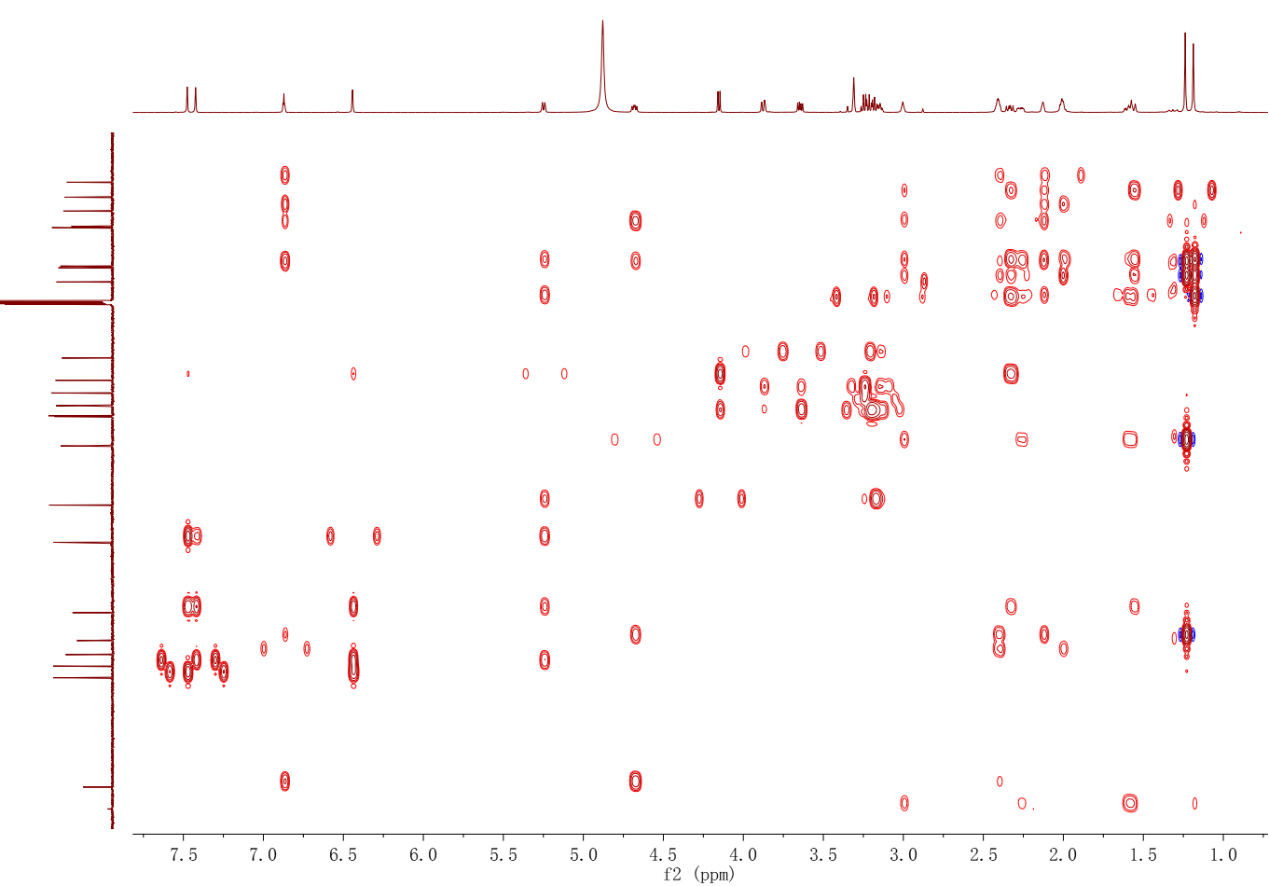


Figure S55. The NOESY spectrum of 8 in CD3OD.


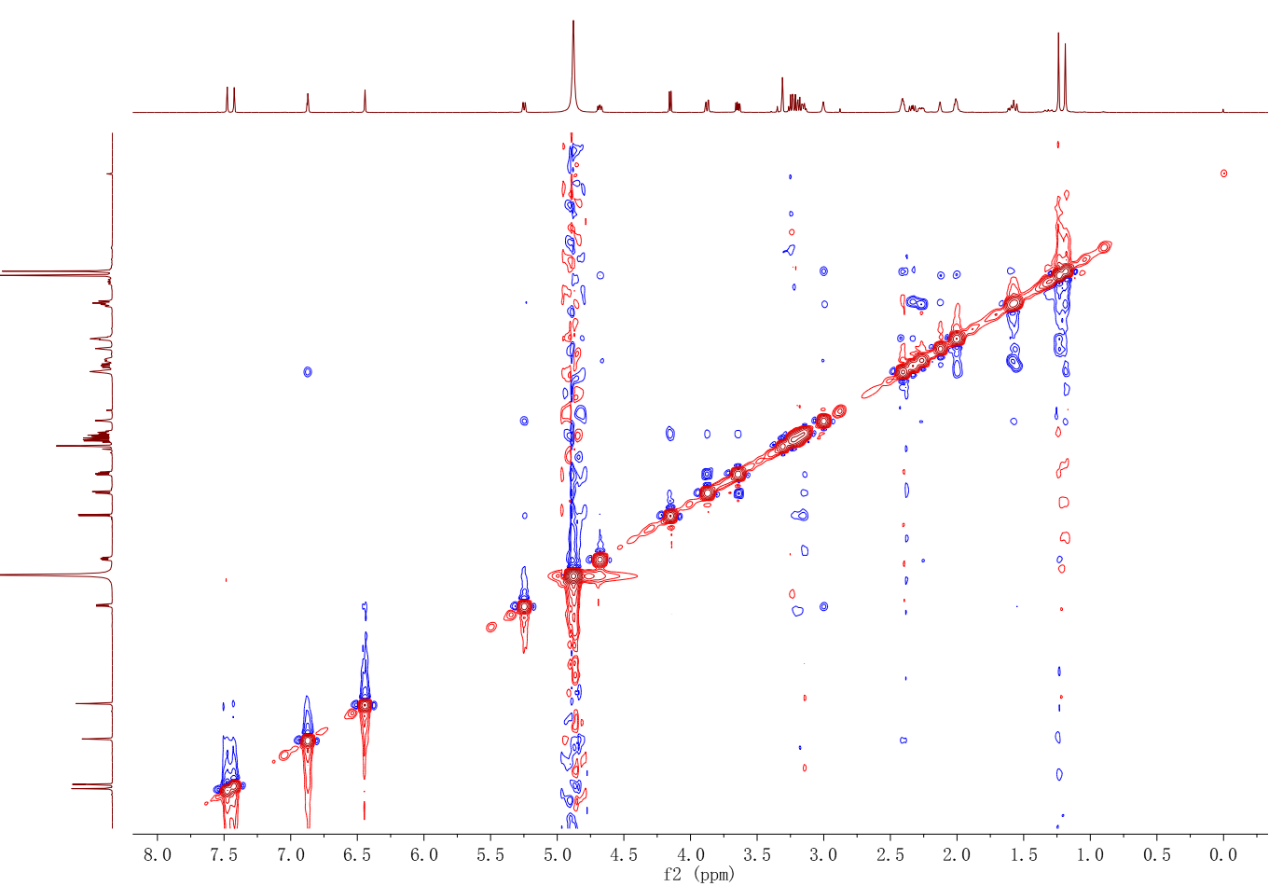


Figure S56. The (+)-HR-ESIMS spectrum of 8.

Figure S57 Subnetwork of tandem MS/MS molecular working for crude extracts of *n*-BuOH part of *T. sadittata*.


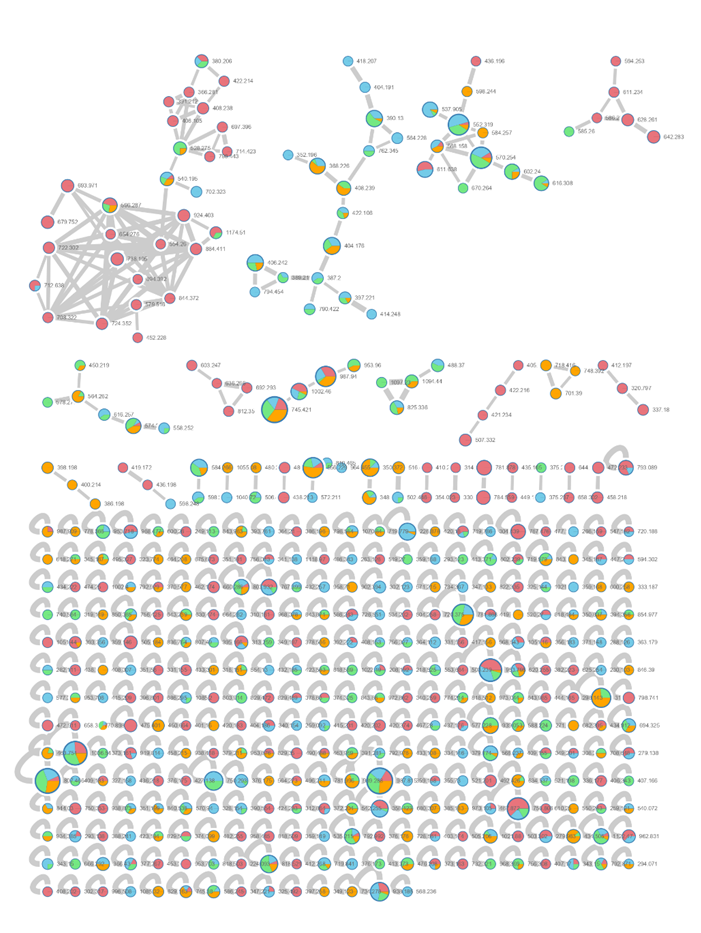


General experimental procedures

Optical rotations were measured on a Rudolph VI polarimeter (Rudolph Research Analytical, USA) using a 10 cm cell. NMR spectra were acquired on a Bruker Avance DRX600 spectrometer (Bruker BioSpin AG, Switzerland), referenced to residual solvent peaks (CD_3_OD: δ_H_ 3.31, δ_C_ 49.0; CDCl_3_: δ_H_ 7.26, δ_C_ 77.0). HR-ESIMS and ESIMS data were obtained using Agilent 6545 Q-TOF and Agilent 1260-6460 Triple Quad LC-MS instruments (Agilent Technologies, Germany), respectively. UV spectra were recorded on a Shimadzu UV-2600 spectrophotometer (Shimadzu, Japan) with a 1 cm path length cell. HPLC separations utilized an Agilent 1260 series LC (Agilent Technologies, Germany) equipped with an Agilent SB-C18 column (9.4 × 250 mm, Agilent Technologies Inc., USA). Column chromatography employed D101-macroporous resin (Sinopharm, China), reversed-phase C18 silica (Merck, Germany), Sephadex LH-20 (GE Healthcare, Sweden), and silica gel (300–400 mesh; Qingdao Marine Chemical, China), using analytical grade solvents (Tianjin Fuyu, China) for CC and HPLC grade solvents (Oceanpak Alexative, Sweden) for HPLC. TLC monitoring used pre-coated silica gel GF254 plates (Qingdao Marine Chemical, China). The mouse RAW264.7 macrophages were purchased from the Chinese Academy of Sciences (Cell Bank of Shanghai Institute of Biochemistry and Cell Biology, China). NO production and cell viability assay detected on a microplate reader (Tecan, Männedorf, Switzerland). Enzyme-linked immunosorbent assay was checked by ELISA kits (Neobioscience Technology Co, Ltd., China). Western-blots were detected by Chemi-Doc XRS system (Bio-Rad, Hercules, CA, USA).

Table S1 Details of crystal data of 12

| Identification code | cu_240802WYY12E_0m |
| --- | --- |
| Empirical formula | C_30_H_44_O_14_ |
| Formula weight | 628.65 |
| Temperature/K | 273.15 |
| Crystal system | monoclinic |
| Space group | C2 |
| a/Å | 15.5653(9) |
| b/Å | 8.8665(6) |
| c/Å | 23.6660(14) |
| α/° | 90 |
| β/° | 105.895(4) |
| γ/° | 90 |
| Volume/Å^3^ | 3141.3(3) |
| Z | 4 |
| ρ_calc_g/cm^3^ | 1.329 |
| μ/mm^‑1^ | 0.889 |
| F(000) | 1344.0 |
| Crystal size/mm^3^ | 0.2 × 0.15 × 0.1 |
| Radiation | CuKα (λ = 1.54178) |
| 2Θ range for data collection/° | 7.768 to 136.884 |
| Index ranges | -18 ≤ h ≤ 18, -8 ≤ k ≤ 10, -28 ≤ l ≤ 28 |
| Reflections collected | 11368 |
| Independent reflections | 4902 [R_int_ = 0.0359, R_sigma_ = 0.0444] |
| Data/restraints/parameters | 4902/41/409 |
| Goodness-of-fit on F^2^ | 1.065 |
| Final R indexes [I>=2σ (I)] | R_1_ = 0.0856, wR_2_ = 0.2384 |
| Final R indexes [all data] | R_1_ = 0.0944, wR_2_ = 0.2520 |
| Largest diff. peak/hole / e Å^-3^ | 0.73/-0.80 |
| Flack parameter | -0.01(10) |
